# RAG recombinase expression discriminates the development of natural killer cells

**DOI:** 10.3389/fimmu.2025.1607664

**Published:** 2025-07-25

**Authors:** Jasmin Sprissler, Ulrich Pannicke, Eva-Maria Rump, Hubert Schrezenmeier, Nicolas Casadei, Michaela Pogoda, Laurence Kuhlburger, Morgana Barroso Oquendo, Stefan Czemmel, Klaus-Michael Debatin, Miriam Erlacher, Klaus Schwarz, Kerstin Felgentreff

**Affiliations:** ^1^ Department of Pediatrics and Adolescent Medicine, University Ulm Medical Center, Ulm, Germany; ^2^ International Graduate School in Molecular Medicine, Ulm University, Ulm, Germany; ^3^ Institute for Transfusion Medicine, Ulm University, Ulm, Germany; ^4^ Institute for Clinical Transfusion Medicine and Immunogenetics Ulm, German Red Cross Blood Service Baden-Württemberg-Hessen, Ulm, Germany; ^5^ German Center for Child and Adolescent Health (DZKJ), Partner Site Ulm, Ulm, Germany; ^6^ Institute of Medical Genetics and Applied Genomics, University of Tübingen, Tübingen, Germany; ^7^ NGS Competence Center Tübingen, Tübingen, Germany; ^8^ Quantitative Biology Center (QBiC), University of Tübingen, Tübingen, Germany

**Keywords:** RAG-fate reporter, induced pluripotent stem cells, NK cell differentiation, V(D)J recombination, DNA damage response, lymphoid progenitor cells, RNA sequencing

## Abstract

**Introduction:**

V(D)J recombination, initiated by recombination-activating gene (RAG) endonucleases, is a crucial process for the generation of diversified antigen receptors of T and B lymphocytes but regarded dispensable for innate natural killer (NK) lymphocytes lacking clonotypic receptors.

**Methods:**

To explore the impact of potential rearrangements on NK cell maturation, RAG-fate mapping reporter human induced pluripotent stem cell (iPSC) lines were generated by introduction of RSS-invEGFP constructs into the AAVS1 locus using CRISPR/Cas9 and differentiated into NK cells *in vitro*.

**Results:**

GFP expression was observed in up to 14% of mature NK cells characterized by a CD45^dim^ CD56^dim^CD57^+^NKG2C^+/−^KIR^+/−^ phenotype and unproductive genetic rearrangements in the *IGH* locus. Advanced maturation was further revealed by transcriptomic studies using RNA sequencing. Despite their strong effector function, DNA damage response and survival to ionizing radiation were compromised.

**Discussion:**

These findings suggest a role of RAG expression in NK cell ontogeny supporting the development of a terminally differentiated effector population.

## Introduction

Human natural killer (NK) lymphocytes develop in the bone marrow and further mature to CD56^dim^CD16^+^ NK cells in secondary lymphoid tissues (SLT) (1, 2). As innate lymphoid cells, they express a series of non-antigen-specific inhibiting and activating receptors (3), and display cell-mediated cytotoxicity and immunomodulatory functions relevant for host defense and immune surveillance. Several concepts for the process of NK cell differentiation have been suggested. The stepwise differentiation from CD34^+^ hematopoietic stem progenitor cells (HSPCs) to mature CD56^dim^CD16^+^ NK cells, defined by surface marker expression, was introduced by Freud and Caligiuri (2). According to this model, NK lymphocytes mature in six stages from CD34^+^CD45RA^+^CD10^+^ progenitors to terminally differentiated CD56^dim^CD16^+^ killer immunoglobulin-like receptor (KIR)-expressing NK cells, which are the most abundant population in peripheral blood. Concurrently, evidence has accumulated that HSPCs are heterogeneous in terms of self-renewal and differentiation properties. Multi-lymphoid progenitors (MLPs), with the potential to differentiate into all types of lymphoid cells in addition to monocytes, macrophages, and dendritic cells (DCs), as well as progenitors with combined myeloid and lymphoid potential (MPPs), have been reported (4–6). Both lymphoid progenitor cells with common T/NK lymphocyte potential (7–10), or B/NK cell potential (4, 11, 12), respectively, have been described in several studies.

Although it is generally accepted that NK cells do not require V(D)J recombination, since they do not express surface T-cell receptors (TCR) or immunoglobulin (Ig) proteins, a large fraction (4%–40%) of human and murine NK cells derive from RAG-expressing progenitors, and have non-productive rearrangements within their Ig and TCR loci (13–15). An impact of V(D)J rearrangements on maturation and function of NK cells has been suggested by mouse and human models (16, 17).

To study the impact of RAG-induced recombination on NK cell differentiation, we generated human iPSC lines with an integrated RAG-fate mapping reporter (18) that permanently labels NK cells with RAG expression in their ontogeny. *In vitro* differentiation of RAG-fate mapping reporter iPSCs into NK cells revealed a distinctive CD45^dim^ NK cell population with a terminally differentiated phenotype in GFP^+^ NK cells. In contrast, GFP^−^ subsets mostly expressed CD45^bright^ and CD56^bright^ and were characterized by reduced maturity and cytotoxic function, but with an increased DNA damage response (DDR) capacity compared to GFP^+^CD45^dim^ NK cells.

Our findings suggest the discrimination of NK cell differentiation by RAG–endonuclease expression in the ontogeny of early NK progenitors.

## Materials and methods

### Cell lines and cell culture

Human iPSCs were generated from healthy human newborn foreskin fibroblasts (NuFFs) C.3 using the CytoTune iPS Sendai Reprogramming kit (Thermo Fisher Scientific) (19). IPSCs were cultured on Vitronectin XF™ (STEMCELL Technologies) in StemMACS™ iPSC Brew XF (Miltenyi) with daily medium change.

Murine stroma cells OP9-DL1 were used as feeders for the differentiation into NK cells. OP9-DL1 were cultured in alpha-MEM medium (without nucleosides) supplemented with 20% heat-inactivated (HI) (56°C for 30 min) fetal bovine serum (FBS) (Biowest™), 1% non-essential amino acids (NEAAs), 1% Glutamax, and 100 U of penicillin and streptomycin (P/S) (Thermo Fisher Scientific).

The murine mastocytoma FcR^+^ cell line P815 was used for functional NK cell studies. P815 was cultured in RPMI supplemented with 20% HI FBS (PAN™ serum), 1% NEAA, 1% Glutamax, and 100 U/ml of P/S.

The human K562 cell line (DMSZ, ACC Nr. 10) was used as target cells for cytotoxicity assays. K562 was cultured with RPMI supplemented with 20% HI FBS (PAN™), 1% NEAA, 1% Glutamax, and 100 U/ml of P/S.

### Generation of reporter IPS cell lines

**Figure 1:**
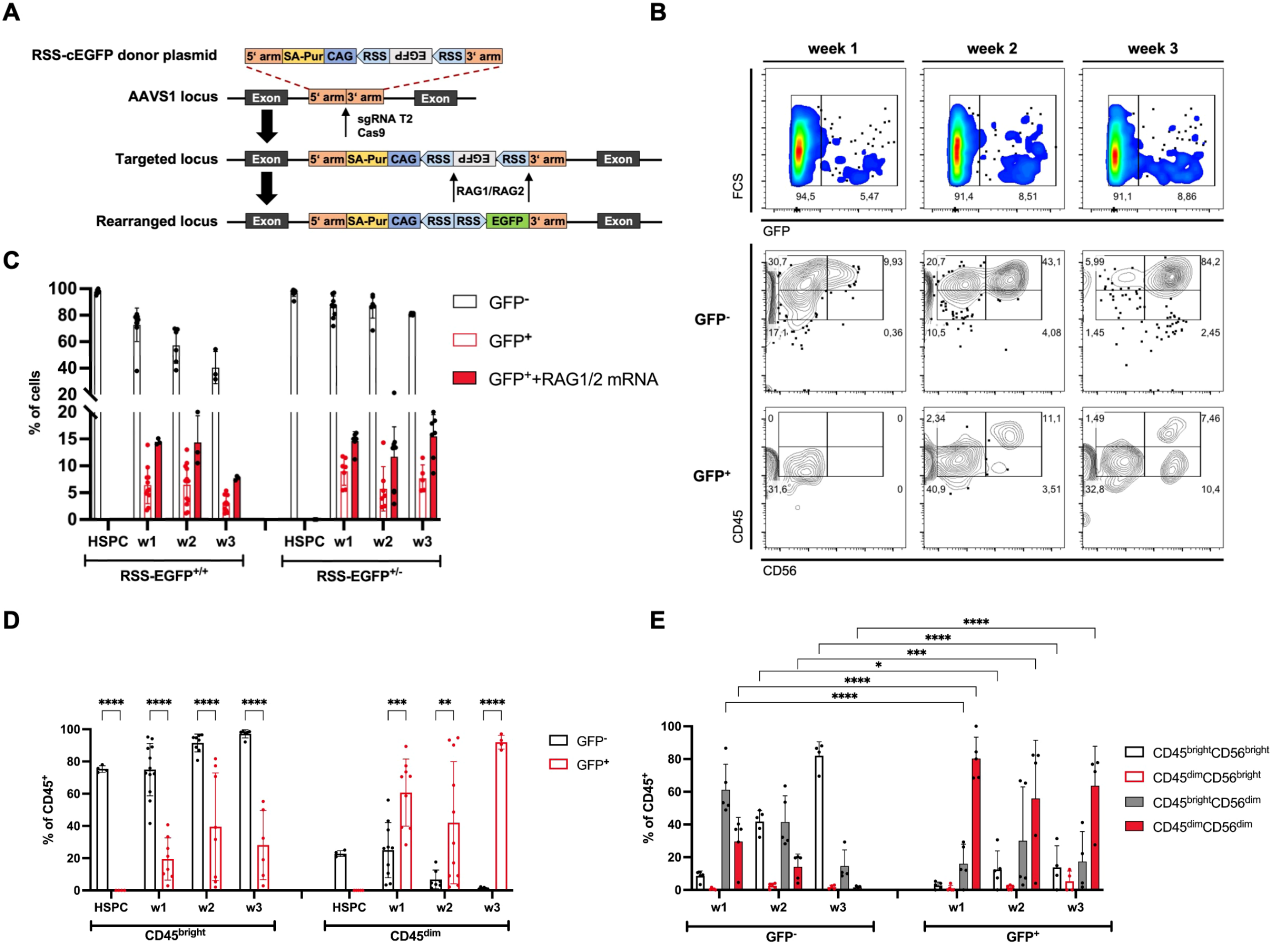
RAG expression can be detected in HSPCs and NK progenitor cells differentiated from RAG-fate-mapping reporter hiPSC lines *in vitro*. **(A)** Schematic illustration of the reporter construct and target site for the generation of RAG-fate-mapping reporter hiPSC lines. A reporter cassette consisting of an inverted EGFP sequence flanked by two RSS was inserted into the AAVS1 locus using CRISPR/Cas9. Targeting by RAG1 and RAG2 recombinases results in flipping of the EGFP cassette into sense orientation. **(B)** Expression of GFP, CD45, and CD56 was analyzed using flow cytometry as demonstrated by this representative gating strategy. GFP^+^ and GFP^−^ NK cells were discriminated by CD45^bright^/CD45^dim^ and CD56^bright^/CD56^dim^ expression, respectively. **(C)** Quantification of GFP expression in HSPCs and NK cells obtained from iPSC with biallelic (RSS–EGFP^+/+^) and monoallelic (RSS–EGFP^+/−^) reporter integration, respectively, was assessed by flow cytometry after hematopoietic differentiation (HSPC), and at weeks (w) 1–3 of the NK cell differentiation. Additional RAG1 and RAG2 mRNA was transfected into HSPCs by nucleofection to induce targeting of the reporter construct. Shown are the percentages of cell populations described in the legend and at indicated time points. **(D)** Distribution of CD45^bright^ and CD45^dim^ expression in GFP^+^ and GFP^−^ NK cell populations, respectively, is shown for NK cells obtained from RSS–EGFP^+/+^ iPSC (CD45^bright^ + CD45^dim^ = 100%). **(E)** Distribution of CD45^bright^CD56^bright^/CD56^dim^ and CD45^dim^CD56^bright^/CD56^dim^ populations is shown for GFP^−^ and GFP^+^ NK cell populations (RSS–EGFP^+/+^), respectively. Shown are means ± SEM obtained from at least three experiments. Statistical analysis was performed using two-way ANOVA. (*p<0.05, **p<0.01, ***p<0.001, ****p<0.0001).

A RAG-fate mapping reporter was introduced into the AAVS1 locus of human iPSC using CRISPR/Cas9 gene editing. Subsequently, iPSCs were investigated for bi- (RSS–EGFP^+/+^) or monoallelic (RSS–EGFP^+/−^) integrations of the reporter construct. We used the vector system developed in the Zhang laboratory (20, 21) (Addgene^®^ 80945 AAVS1-Pur_CAG-EGFP) and replaced the EGFP cassette with an inverted EGFP cassette flanked by two RSS sequences (18, 22). Cloning was performed using the Fusion™ HD Transformation Kit Eco Dry (Takara). Targeting of the inverted EGFP cassette by the nucleases RAG1 and RAG2 results in flipping of the cassette into sense orientation (**Figure 1A**). CRISPR/Cas9 protein and gRNA were transfected into iPSCs by nucleofection (Amaxa Kit V, Roche). Transfected iPSC clones were selected using 1 ng/µl of puromycin (Thermo Fisher Scientific). Stable vector integration was confirmed by PCR (**Supplementary Figure S1A**) and Sanger sequencing, and the number of integrations was determined by RT-PCR (**Supplementary Figure S1B**). Pluripotency and karyotypic integrity were confirmed in generated reporter iPSC lines (**Supplementary Figures S1C–E**) as described in the supplementary methods.

### Differentiation of iPSC into hematopoietic stem progenitor cells

iPSCs were cultured in six-well plates coated with Vitronectin XF™ (STEMCELL Technologies) to 60%–100% confluency. Colonies were cut into squares using the StemPro™ EZPassage™ Disposable Stem Cell Passaging Tool (Thermo Fisher Scientific), transferred into one well of a six-well ultra-low attachment plate, and incubated overnight. Cell clusters developed into EBs of different sizes. To induce differentiation toward HSPCs, the STEMdiff™ Hematopoietic Kit (STEMCELL Technologies) was used according to manufacturer’s instructions. A maximum of five to seven EBs were transferred into a well of a 12-well plate coated with Vitronectin XF™ and containing 1 ml of differentiation medium A (1 ml/12 well). On day 12, the suspension cells were harvested and passed through a 40-μm cell strainer. No positive selection of HSPCs was performed prior to NK cell differentiation.

### Differentiation of HSPCs into NK cells

HSPCs were differentiated into NK cells on OP9-DL1 feeder cells as described before (19). OP9-DL1 feeder cells/well (1 × 10^5^) were seeded into 12-well culture plates and treated with 10 mg/µl of mitomycin C (MMC) (STEMCELL Technologies) for 3 h on the following day. HSPCs (1 × 10^4^) were plated on feeder cells and cultured in 2 ml of NK cell differentiation medium [56.6% DMEM high glucose (Thermo Fisher Scientific), 28.3% Ham’s F-12 Nutrient Mix (Thermo Fisher Scientific), 15% heat-inactivated human AB serum (Valley Biomedical), 2 mM L-glutamine (Thermo Fisher Scientific), 25 μM β-mercaptoethanol (Thermo Fisher Scientific), 5 ng/ml of sodium selenite (Sigma-Aldrich), 50 μM ethanolamine (Sigma-Aldrich), 20 mg/l of L-ascorbic acid (Sigma-Aldrich), 1% NEAA, 1% P/S supplemented with 10 ng/ml of recombinant human stem cell factor (SCF), 10 ng/ml of recombinant human IL-7, 10 ng/ml of recombinant human IL-15, 10 ng/ml of recombinant human Flt3 ligand, and 10 ng/ml of recombinant human IL-3 (PeproTech). IL-3 was only used until day 9. Half medium changes were performed on days 3, 6, 9, 13, and 16. On days 6 and 13, progenitor cells were replated on fresh feeder cells. On days 6 [week 1 (w1)], 13 [week 2 (w2)], and 20 [week 3 (w3)], cells were analyzed for surface marker expression using flow cytometry. OP-DL1 stroma cells were separated from NK suspension cells by filtering through a 40-µm strainer.

### Flow cytometry

For flow cytometry, 1 × 10^4^–1 × 10^5^ cells were collected, washed twice with PBS, and stained with antibody mixes at concentrations of 1:100 in 100 µl of staining buffer (PBS, 1% FCS) for 15 to 30 min at room temperature (RT) in the dark. Antibodies and isotypes used are listed in **Supplementary Table S1**. For intracellular staining of perforin, granzyme B, and interferon gamma, Cytofix/Cytoperm (BD Biosciences) was used for fixation and permeabilization according to the manufacturers’ instructions. The FoxP3 Staining Buffer Set (Miltenyi Biotec) was used for permeabilization of iPSCs characterized for pluripotency and trilineage differentiation. Cells were subsequently stained with antibody mixes at concentrations up to 1:100 in 100 µl of Perm Wash or FoxP3 Staining Buffer, respectively, for 30–60 min at 4°C in the dark. After two washes, cells were resuspended in 50–100 µl of PBS or staining buffer and analyzed on a BD FACSAria I™ or BD FACSAria III™ Cell Sorter (BD Biosciences), respectively.

### Functional NK cell assays

To assess degranulation, cytokine production, and antibody-dependent cellular cytotoxicity (ADCC), NK cells generated on OP9-DL1 cells were harvested after week 3 and co-incubated with human K562 erythroleukemia tumor cells (E:T of 3:1 and 6:1), or mouse P815 mastocytoma cells (E:T of 3:1 and 6:1), respectively. The latter were either uncoated or coated with anti FCyRIIIa (a-CD16, 10 µg/µl; Biolegend) or isotype control (10 µg/µl) for 1 h. Prior to effector cell co-incubation, target cells were labeled using the CellTrace™ Far Red Cell Proliferation Kit (1 µg/ml, Thermo Fisher Scientific) for 20 min.

### DNA damage response and survival in response to ionizing radiation

DNA damage was induced in NK cells obtained at weeks 1, 2, and 3 using 2 Gy of ionizing radiation from a Cs134 source. Cells were fixed and permeabilized after 2, 4, 8, and 24 h using solution A of Fix & PERM (Thermo Fisher Scientific) mixed 1:1 (w/w) with culture medium. After 3 min of incubation at room temperature, 2 ml of ice-cold methanol was added. In addition to samples obtained at indicated time points, an unirradiated control and an isotype control obtained 2 h after radiation were prepared. Subsequently, cells were stained with APC anti-H2A.X-phosphorylated (Ser139) (Biolegend), or APC mouse IgG1, K isotype control (Biolegend) for 1 h in the dark.

### V(D)J recombination and IGH repertoire

NK cells harvested at week 3 of differentiation were screened for V(D)J recombination events at the IGH, IGK, IGL, TCRB, TCRG, and TCRD loci using a multiplex PCR reported before (23). Genomic DNA was isolated from sorted GFP^+^ and GFP^−^ NK progenitor cells using the QIAamp DNA Micro kit (Qiagen).

To study V(D)J joints in the IGH locus of NK progenitor cells by next-generation sequencing (NGS), 1,000 GFP^+^ NK cells (derived from RSS–EGFP^+/+^ iPSC) were sorted from differentiation cultures at weeks 1, 2, and 3, and gDNA was isolated. Three replicates were prepared for each sample that was obtained from three independent experiments. NGS of the IGH repertoire was performed by Adaptive Biotechnologies, and analysis tools provided by Adaptive were used. Heat maps were generated using the open-access webtool Morpheus provided by the Broad Institute, Cambridge, MA, USA (Morpheus, https://software.broadinstitute.org/morpheus).

### RNA sequencing

For RNA sequencing analysis, 500 GFP^+^ and GFP^−^ NK progenitor cells (derived from RSS–EGFP^+/+^ iPSC) were sorted on days 19 (week 1), 26 (week 2), and 33 (week 3) into 25 µl of NEBNext^®^ cell lysis buffer (New England Biolabs). Three replicates from three independent experiments were analyzed for each sample and time point.

Synthesis of cDNA, library preparation, and sequencing were performed by the c.ATG genomics core facility at the University Hospital Tuebingen, Tuebingen, Germany, and bioinformatic data analysis was performed with the bioinformatics core facility QBiC (University of Tuebingen, Tuebingen, Germany). RNA sequencing and the bioinformatic analysis are described in detail in the supplementary section.

GO enrichment analysis was performed using the Gene Ontology Resource (https://geneontology.org/) (GO Ontology database DOI: 10.5281/zenodo.12173881 Released 2024-06-17), PANTHER™ (version 19, released 2024-06-20), and Reactome (version 86, released 2023-09-07) (24–26). Results can be retrieved from **Supplementary Table S2**. Genes associated with B (GO_0030183) and NK lymphocyte (GO_0001779) development were identified using Ensembl BioMart (Ensembl 111) (https://www.ensembl.org/info/data/biomart/index.html).

### Statistical analysis and data visualization

Each experiment was repeated at least three times, and means were used for statistical evaluation. Data were analyzed using GraphPad Prism 9.0 Software. The type of statistical analysis performed and the subset of the study population are listed in the figure legends. Statistical significance is indicated by p-values (*p < 0.05, **p < 0.01, ***p < 0.001, ****p < 0.0001). The web-based platforms Galaxy (27) and SRplot (28) were used for visualization of transcriptomic data. Dot plots were generated using the MATLAB^®^ Engine API for Python^®^.

## Results

### RAG recombinase is predominantly expressed in CD45^dim^ NK cell progenitors

To study the expression of RAG recombinase in NK cell development, we introduced a reporter cassette consisting of an inverted EGFP sequence flanked by two recombination signal sequences (RSS) (18) into the AAVS1 locus of human induced pluripotent stem cells (iPSCs) using CRISPR/Cas9 (20, 21) (**Figure 1A**). Efficient targeting of RSS by RAG1 and RAG2 results in flipping of the EGFP cassette into sense orientation and permanently marks the ontogeny of RAG expression. Cells with successfully integrated reporter constructs were selected by culture with puromycin (1 ng/µl) and subsequently screened for stable reporter integration using PCR (**Supplementary Figure S1A**), Sanger sequencing, and copy number variation (CNV) determined by qPCR (**Supplementary Figure S1B**). iPSC lines were characterized for their karyotypic integrity and retained pluripotency by expression of pluripotency-associated genes and proteins (**Supplementary Figures S1C, D**). In addition, iPSCs were differentiated into all three germ layers confirmed by surface expression of SOX2 and PAX6 (ectoderm), SOX17 and CD184 (CXCR4) (endoderm), and CD144 (VE-cadherin) and CD140b (mesoderm) (**Supplementary Figure S1E**). To validate the efficiency of the reporter construct, RAG1 and RAG2 mRNAs were introduced into iPSCs and HSPCs using nucleofection. As reported before (29), iPSCs were not able to perform V(D)J recombination, but efficient rearrangement of the reporter cassette was observed in HSPCs (**Supplementary Figure S1F**). Based on these investigations, iPSC lines containing bi- (RSS-EGFP^+/+^) and monoallelic (RSS-EGFP^+/−^) integrations of the reporter construct were selected and further differentiated into HSPCs and NK cells on OP1-DL1 stroma cells *in vitro*. Expression of GFP and NK lineage markers determining the process of differentiation was monitored weekly using flow cytometry (**Supplementary Figure S1G**). For simplification, we designated cells obtained after completion of the hematopoietic differentiation protocol “HSPCs” and cells in the process of differentiation toward the NK lymphocyte lineage “NK cell progenitors” or “NK cells.”

Importantly, two populations of CD45- and CD56-expressing cells could be identified that were discriminated as CD45^bright^ and CD45^dim^, and CD56^bright^ and CD56^dim^, respectively (**Figure 1B**).

GFP expression could be detected in up to 14% of NK cell progenitors from day 3 of differentiation on OP9-DL1 stroma cells in fluctuating intensities (**Figure 1C**). Of note, no GFP expression could be observed in HSPCs. Additional transduction of RAG1 and RAG2 mRNAs at the stage of HSPCs increased the fraction of GFP^+^ cells in NK cell precursors (14%–16.1% at week 1, 10.5%–21.4% at week 2, and 7.3%–20% at week 3) (**Figure 1C**) in RSS–EGFP^+/+^ and RSS–EGFP^+/−^ reporter iPSC lines, respectively. Most NK cells obtained *in vitro* expressed GFP^−^CD45^bright^CD56^bright^, but GFP^+^ NK cells were found in up to 90% of the CD45^dim^ NK cell population at all stages of differentiation (**Figure 1D**, **Supplementary Figure S2A**). These populations could be further discriminated by CD56 expression, whereby CD45^bright^ NK cells predominantly expressed CD56^bright^, and CD45^dim^ NK cells, CD56^dim^ (**Figure 1E**, **Supplementary Figure S2B**).

### RAG expression ontogeny impacts on CD45 isotype expression in NK cell progenitors

The observation of CD45^bright^ and CD45^dim^ populations in GFP^+^ and GFP^−^ NK progenitors was further investigated by studying the CD45RA, CD45RB, CD45RC, and CD45RO isotype expression using flow cytometry (**Supplementary Figure S2C**). CD45RB and CD45RC expression was analyzed in CD45RA^+^, CD45RO^+^, CD45RA^+^RO^+^, and CD45RA^−^RO^−^ populations.

Peripheral blood NK cells isolated from three healthy buffy coat donors stained predominantly CD45^bright^ and expressed CD45RA in co-expression with CD45RB and CD45RC (CD45RA/RC/RB and CD45RA/RC) isotypes (**Supplementary Figures S2D, E**). Since different CD45 isotypes can be co-expressed (30), we analyzed all possible combinations in CD45^bright^ and CD45^dim^ HSPCs, and GFP^−^/GFP^+^ NK cell progenitors obtained at weeks 1, 2, and 3, respectively (**Supplementary Figures S3A, B**).

IPSC-derived HSPCs that were all GFP^−^ due to lack of recombination at this stage, expressed predominantly all CD45 isotypes (RA/RO/RB/RC) and CD45RA/RC/RB. On CD45^dim^ HSPCs, up to 16% CD45RB, or CD45RB/RC isotypes could be detected. No specific CD45 isotype could be identified in up to 20% of HSPCs suggesting a lack of lymphocyte commitment (**Supplementary Figures S3A–D**).

CD45^bright^ NK cells were characterized by CD45RA/RB/RC and CD45RA/RC in GFP^−^ and CD45RA/RO/RB/RC, and CD45RA/RO/RB in GFP^+^ NK cells, respectively. In contrast, CD45^dim^ NK cells obtained at weeks 1–3 expressed predominantly CD45RO, whereas co-expression of CD45RB could be identified on GFP^+^ cells (**Supplementary Figures S3A–D**).

In summary, CD45 isotype distribution differs in NK progenitors with and without RAG-fate ontogeny with a preference for CD45RO and CD45RB in GFP^+^ and CD45RA in GFP^−^ NK cells. The different CD45 isotype distribution results in the observation of CD45^dim^ and CD45^bright^ NK progenitors.

For technical reasons, the following studies were performed on CD45^bright^ and CD45^dim^ NK cells without consideration of the CD45 isotypes.

### RAG-fate-mapped NK cells are characterized by increased maturity

The maturation process of differentiating NK cells was monitored weekly for expression of NK lineage marker using flow cytometry (**Supplementary Figure S1G**). HSPCs were harvested after 12 days of hematopoietic differentiation and analyzed for CD34, CD43 (leucosialin), CD45, and CD15 surface marker expression (**Supplementary Figure S4A**). No GFP expression, but CD45^dim^ and CD45^bright^ populations, were detected at the stage of HSPCs that expressed mainly CD45^bright^ and up to 25% CD45^dim^. An increased fraction of CD34^+^CD43^−^ cells was found in CD45^dim^ compared to that in CD45^bright^ HSPCs, which predominantly stained CD34^+^CD43^+^ (**Supplementary Figures S4B, C**).

**Figure 2:**
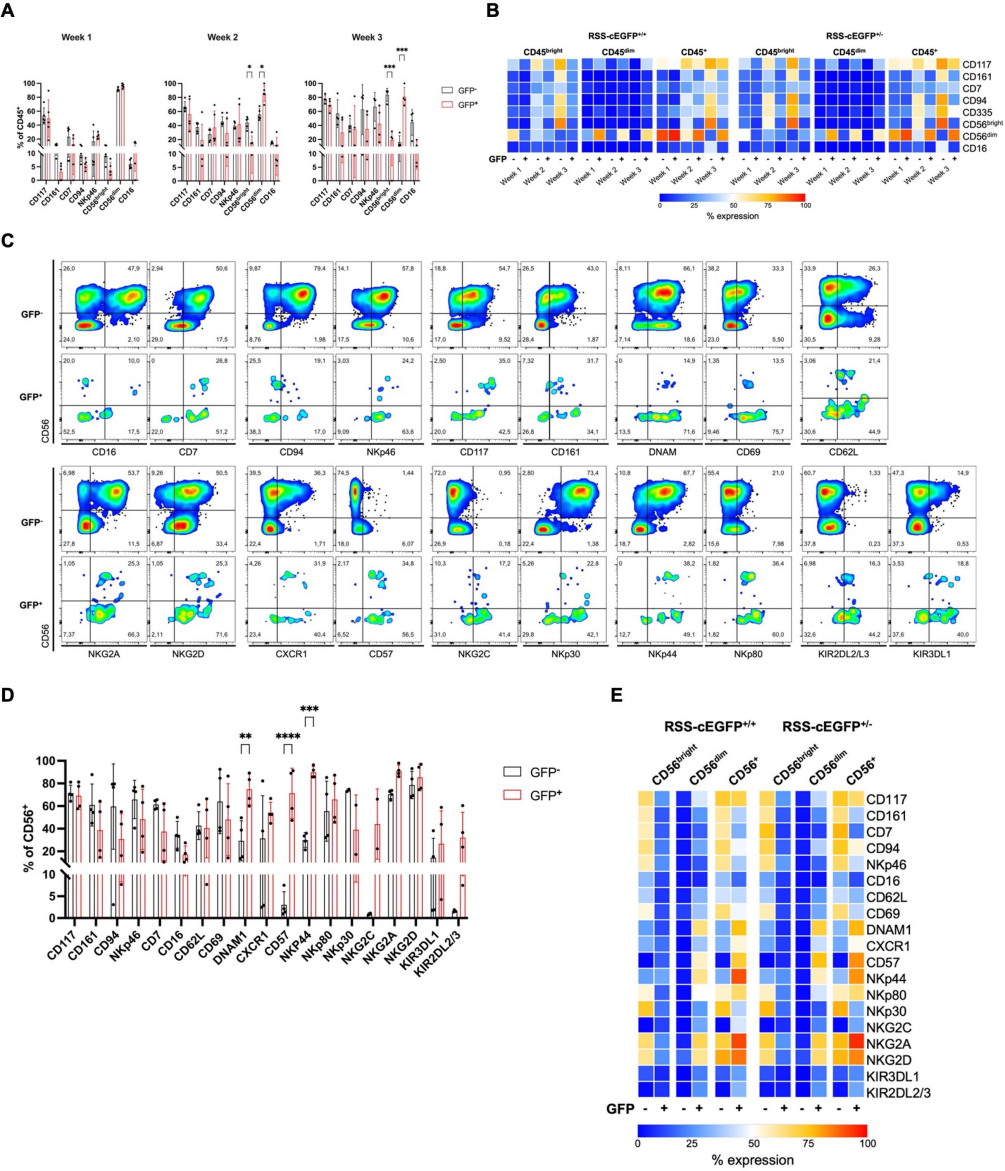
NK lineage surface marker expression indicates a more mature phenotype in RAG-fate-mapped NK cells. **(A)** RAG-fate-mapping reporter iPSCs were differentiated into NK cells and studied weekly for NK lineage marker expression using flow cytometry. Expression of indicated surface markers were analyzed in GFP^+^ versus GFP^−^ CD45^+^ NK cell precursors (RSS–EGFP^+/+^). **(B)** Mean expression profiles are shown as percentages in heatmaps for CD45^bright^, CD45^dim^, and total CD45^+^ NK lymphocytes. **(C)** Expression of indicated NK lineage markers was studied in GFP^+^ versus GFP^−^ CD56^bright^, CD56^dim^, and total CD56^+^ NK cell precursors, respectively. Shown is the gating strategy performed on one representative sample obtained at week 3 of the differentiation protocol gated on CD45^+^CD56^+^ cells. **(D)** Expression of indicated NK cell marker was studied in GFP^+^ versus GFP^−^ CD56^+^ NK cell precursors (RSS–EGFP^+/+^). **(E)** The mean expression levels of NK cell marker analyzed are shown as percentage distributions in a heatmap for both RSS–EGFP^+/+^ and RSS–EGFP^+/−^ iPSC-derived cells, respectively. Statistical analysis was performed using 2Way ANOVA (*p<0.05, **p<0.01, ***p<0.001, ****p<0.0001).

From weeks 1–3 of the NK cell differentiation, expression of CD117 (c-kit), CD7, CD161, CD94, CD335 (NKp46), CD56, and CD16 was assessed on CD45^bright^ and CD45^dim^ NK cell progenitors (**Supplementary Figure S5A**). Expression of NK cell markers was compared between RAG-fate-mapped (GFP^+^) and non-mapped (GFP^−^) CD45^bright^, CD45^dim^, and total CD45^+^ precursors (**Figures 2A, B**, **Supplementary Figures S5B–D**). CD56 expression could be observed from week 2 with a phenotype of predominantly CD56^bright^ in GFP^−^ and CD56^dim^ in GFP^+^ NK cell precursors. Although CD117 expression was similar in both GFP^−^ and GFP^+^ precursors increasing from week 1 (mean 54% ± 9.4% in GFP^−^, mean 51.5% ± 16.3% in GFP^+^) to weeks 2 and 3 (mean 78.5% ± 4.7% in GFP^−^, mean 68.7% ± 6.1% in GFP^+^), there were significant differences between GFP^-^CD45^bright^ and GFP^+^CD45^dim^ NK cells (**Supplementary Figures S5B–D**). CD161 and CD94, the latter of which participates in the heterodimeric complex of NK2G receptors, were both increasingly expressed from week 2 to 3, and to a significantly higher extent in GFP^−^ NK cells (RSS–EGFP^+/−^). The natural cytotoxicity receptor (NCR) NKp46 (CD335) was observed from week 2 and expressed by up to 78% (±13.4%) GFP^−^ and 85% (±26.9%) GFP^+^ NK cells at week 3. Expression of CD16 accelerated from week 2 to 3 and could be detected on 8%–30% (±8.5%) of GFP^−^CD45^bright^ NK cells compared to 5%–19% (±8.0%) of GFP^+^CD45^dim^ cells at week 3. Of note, GFP^−^CD45^dim^ cells stained negative for NK lineage markers investigated in this study (**Figure 2B**, **Supplementary Figure S5C, D**).

NK lineage marker were further analyzed on CD56^+^ NK cells obtained at week 3, which were additionally evaluated for markers of activation (CD69, DNAM1, CD62L), maturity (CD57, CXCR1), activating cytotoxicity receptors (NCRs) NKp46 (NCR1, CD335), NKp44 (NCR2, CD336), NKp30 (NCR3, CD337), as well as activating (NKG2C, NKG2D) and inhibiting (NKG2A) transmembrane NKG2/CD94 receptors, and killer cell immunoglobulin-like receptors (KIRs) KIRDL2/L3 and KIR3DL1 (**Figures 2C–E**, **Supplementary Figure S6A–C**).

In contrast to GFP^−^ cells, CD56^dim^ RAG-fate-mapped NK cells were characterized by predominant expression of the senescence marker CD57. In addition, NKG2C and KIRs could be found on GFP^+^ NK cells, whereas these markers of terminal differentiation were completely absent on GFP^−^ cells. The NCRs NKp30, NKp44, and NKp46 were differentially expressed, with a predominance of NKp30 and NKp46 in GFP^−^, and NKp44 in GFP^+^ cells, respectively. This suggests a higher activation status in the latter, whereas NKp30 and NKp46 are expressed on resting cells (31). In addition, GFP^+^ NK cells were characterized by an elevated fraction of DNAM1^+^ NK cells (**Figures 2D, E**, **Supplementary Figure S6A–C**). Expression of most NK lineage markers was reduced in GFP^+^CD56^bright^ compared to that in GFP^−^CD56^bright^ cells and almost absent in GFP^−^CD56^dim^ NK cells (**Figure 2E**, **Supplementary Figures S6B, C**). Similar results were obtained for both reporter cell lines, RSS–EGFP^+/−^ and RSS–EGFP^+/+^.

These results suggest accelerated differentiation and a more mature phenotype in GFP^+^ versus GFP^−^ NK precursor cells.

### Transfection of additional RAG1 and RAG2 mRNA increases the event of recombination and leads to a phenotype of increased maturity in RAG-fate-mapped NK cells

To address the question whether the phenotype observed in GFP^+^ NK cells can be attributed to targeting of RAG endonucleases, RAG1 and RAG2 mRNA was transfected into HSPCs derived from reporter iPSCs. The increased targeting rate of the reporter construct raised the percentage of GFP^+^ NK cells up to 21.4% (**Figure 1C**). As in cells mapped by endogenous RAG expression, CD45^dim^ cells were predominantly found in GFP^+^ NK cells (**Supplementary Figure S7A**). Furthermore, CD45^dim^ NK cells expressed mostly CD56^dim^, whereas CD56^bright^ expression was reduced in RAG1-/RAG2-transfected GFP^+^ NK cells (**Supplementary Figures S7B, C**).

The phenotype of NK cells with induced RAG1/RAG2 expression was investigated regarding NK lineage marker expression and maturity at weeks 1–3 of the NK cell differentiation. Interestingly, phenotypic differences between GFP^−^ and GFP^+^, and CD45^bright^ and CD45^dim^ NK cells resembled NK cells mapped by endogenous RAG1/RAG2 expression (**Supplementary Figures S7D–F**). In contrast to RAG-fate-mapped NK cells, expression of CD94, NKp46, and CD56^bright^ accelerated in GFP^−^ cells from week 1–3.

Following 3 weeks of differentiation, NK lineage surface markers were investigated on CD56^+^ cells (**Supplementary Figures S8A–G**). The expression of DNAM1, CD57, CXCR1, and NKp44 was significantly increased in GFP^+^ compared to that in GFP^−^ NK cells, whereas elevated fractions of NKp30^+^ and NKp46^+^ were detected in the GFP^−^ population (**Supplementary Figures S8E, F**). NKG2C and KIR2DL2/3 could only be detected on GFP^+^ NK cells. Though expressed at low levels on both populations, CD16 was predominantly found on GFP^−^ NK cells.

In summary, these data show that additional transfection of RAG1 and RAG2 mRNA results in increased targeting of the reporter construct, which contrasts the differential phenotype toward increased maturity observed in RAG-fate-mapped compared to non-mapped NK cells.

### RAG-fate-mapped NK cells demonstrate an increased potential for both degranulation and cytokine production over non-mapped NK cells

**Figure 3:**
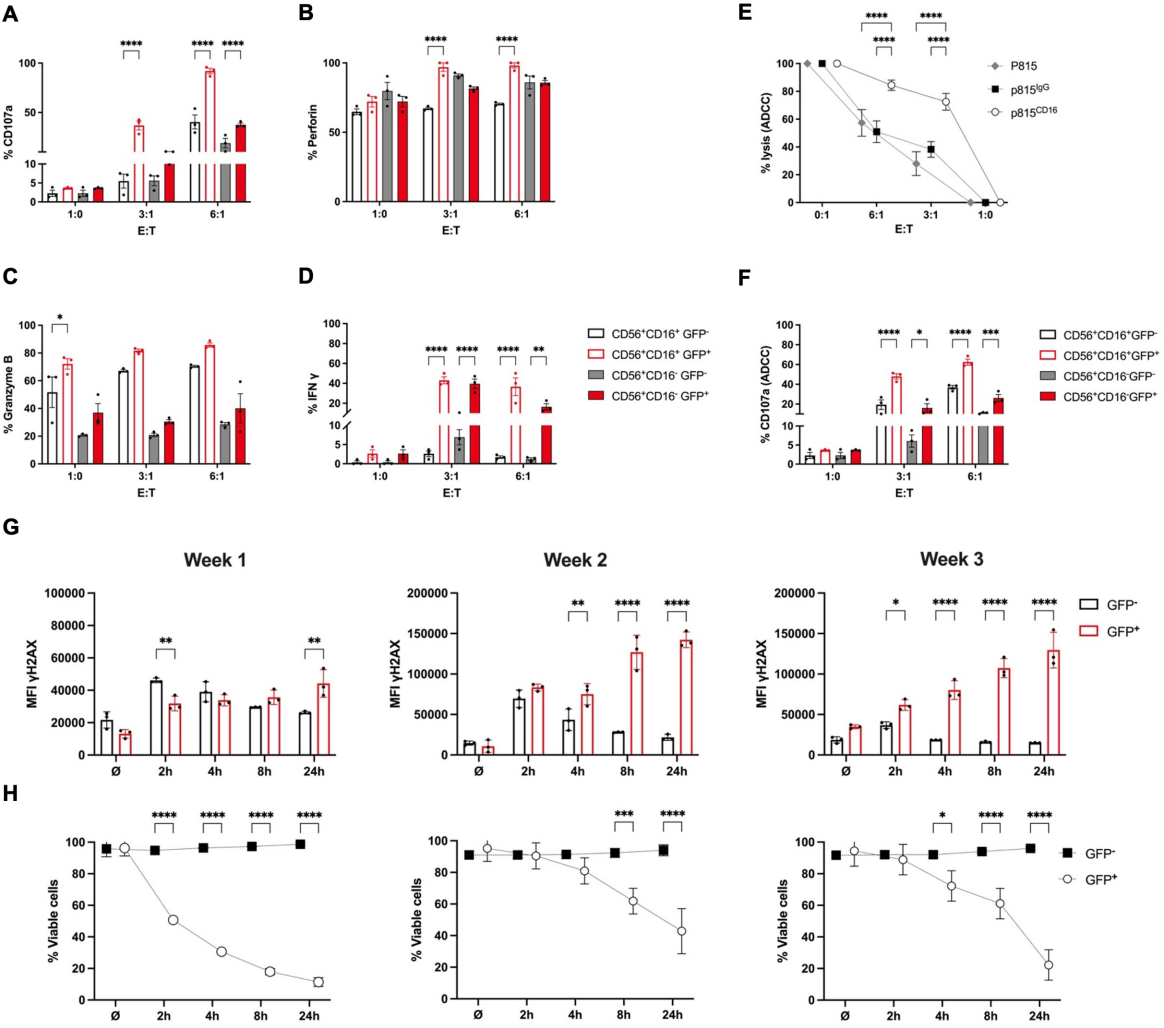
RAG-fate-mapped NK cells are characterized by increased cytokine expression and cytotoxic potential, but impaired DNA damage response. iPSCs carrying bi-allelic reporter constructs (RSS–EGFP^+/+^) were differentiated into NK cells over 3 weeks and subsequently harvested for functional studies. Cells were cocultured with K562 target cells labeled with cell tracer dye at indicated effector:target (E:T) ratios for 1 (h) Expression of CD107a **(A)**, perforin **(B)**, granzyme B **(C)**, and interferon gamma **(D)** was determined in GFP^+^ and GFP^−^ CD56^+^CD16^−^ and CD56^+^CD16^+^ NK cells, respectively, using flow cytometry. Percentual expression is shown. **(E)** The antibody-dependent cytotoxicity (ADCC) of NK cells obtained at week 3 of differentiation was quantified using the murine mastocytoma cell line P815. Murine P815 target cells were coated with human anti-CD16 or IgG isotype, respectively, labeled with CellTrace™, and cocultured with NK cells at indicated effector:target (E:T) ratios. Uncoated p815 cells served as control. **(F)** Degranulation capacity of GFP^+^/GFP^−^ and CD56^+^CD16^−^/CD56^+^CD16^+^ NK cells, respectively, was measured by CD107a expression in response to coculture with anti-CD16-coated P815 target cells at indicated effector:target (E:T) ratios. Percentage of CD107a-expressing cells is shown on a linear scale. **(G)** NK cells obtained at weeks 1–3 of differentiation were irradiated with 2 Gy and fixed at indicated time points. Geometric mean fluorescent intensities (MFIs) of γH2AX are shown for GFP^+^ and GFP^−^ NK cell populations at indicated time points after irradiation. **(H)** The survival responses were analyzed in the respective GFP^+^ and GFP^−^ populations at indicated time points after 2-Gy ionizing radiation. The percentage of vitality was calculated based on survival rates of unirradiated cells. Shown are results obtained from RSS–EGFP^+/+^ iPSC as means ± SEM from at least three experiments. Statistical analysis was performed using two-way ANOVA (*p<0.05, **p<0.01, ***p<0.001, ****p<0.0001).

Degranulation capacity quantified by expression of CD107a, as well as intracellular perforin, granzyme B, and interferon gamma (IFNγ), was investigated in mature iPSC-derived NK cells harvested at week 3 and cocultured with K562 cells at defined effector:target (ET) ratios using flow cytometry. To assess cytotoxicity and cell lysis of target cells, the latter were labeled using a cell tracer dye. Compared to GFP^−^ populations, GFP^+^CD56^+^CD16^+/−^ mature NK cells displayed increased degranulation capacity shown by CD107a expression in response to K562 coculture (**Figure 3A**). Perforin, but not granzyme B, expression differed moderately between GFP^−^ and GFP^+^ NK cells at 3:1 and 6:1 effector:target ratios (**Figures 3B, C**). Interestingly, GFP^+^ NK cells produced more IFNγ in response to stimulation with PMA/ionomycin compared to GFP^−^ NK cells (**Figure 3D**). The redirected antibody-dependent cytotoxicity (ADCC) was measured using anti-CD16-coated murine FcR^+^ P815 cells as target for iPSC-derived NK cells (32) (**Figure 3E**). Degranulation in response to coated P815 cells was increased in GFP^+^ compared to GFP^−^ CD56^+^CD16^+/−^ NK cell populations (**Figure 3F**).

These results demonstrate that iPSC-derived NK lymphocytes are functional, and RAG-fate-mapped NK cells have an increased potential for both degranulation and cytokine production.

### RAG-fate-mapped NK cells show an impaired DNA damage response capacity and diminished cellular survival in response to ionizing radiation

NK progenitor cells obtained at weeks 1, 2, and 3 were irradiated with 2 Gy and fixed after 2, 4, 8, and 24 h. Mean fluorescence intensities (MFIs) of the DDR marker γH2AX were measured using flow cytometry. Phosphorylation of the histone protein H2AX (γH2AX) occurred shortly after induction of DNA damage in GFP^−^ NK cells and was followed by subsequent downregulation of γH2AX foci upon repair. However, γH2AX level remained constantly increased in GFP^+^ populations following IR (**Figures 3G**, **Supplementary Figure S9A**). This finding suggests persistent DNA damage signaling in RAG-fate-mapped NK cells due to senescence. Accordingly, cellular survival after IR was severely reduced in GFP^+^ in contrast to that in GFP^−^ NK cells (**Figure 3H**, **Supplementary Figure S9B**).

### NK cells with RAG expression ontogeny have unproductive rearrangements in the IGH locus

To verify targeting by RAG recombinases, we searched for rearrangements in heavy chains and kappa and lambda light chains of the immunoglobulin receptor, and rearrangements in the TCR beta, gamma, and delta locus in sorted GFP^+^ NK cells obtained after 3 weeks of differentiation (23) (**Supplementary Figure S10A**). Since rearrangements were observed on the IGH, but not on the TCR locus, the genomic IGH repertoire was studied in gDNA extracted from sorted GFP^+^ NK cells at weeks 1, 2, and 3 using NGS analysis. Three independent experiments were performed, and results were calculated on three replicates of each sample.

**Figure 4:**
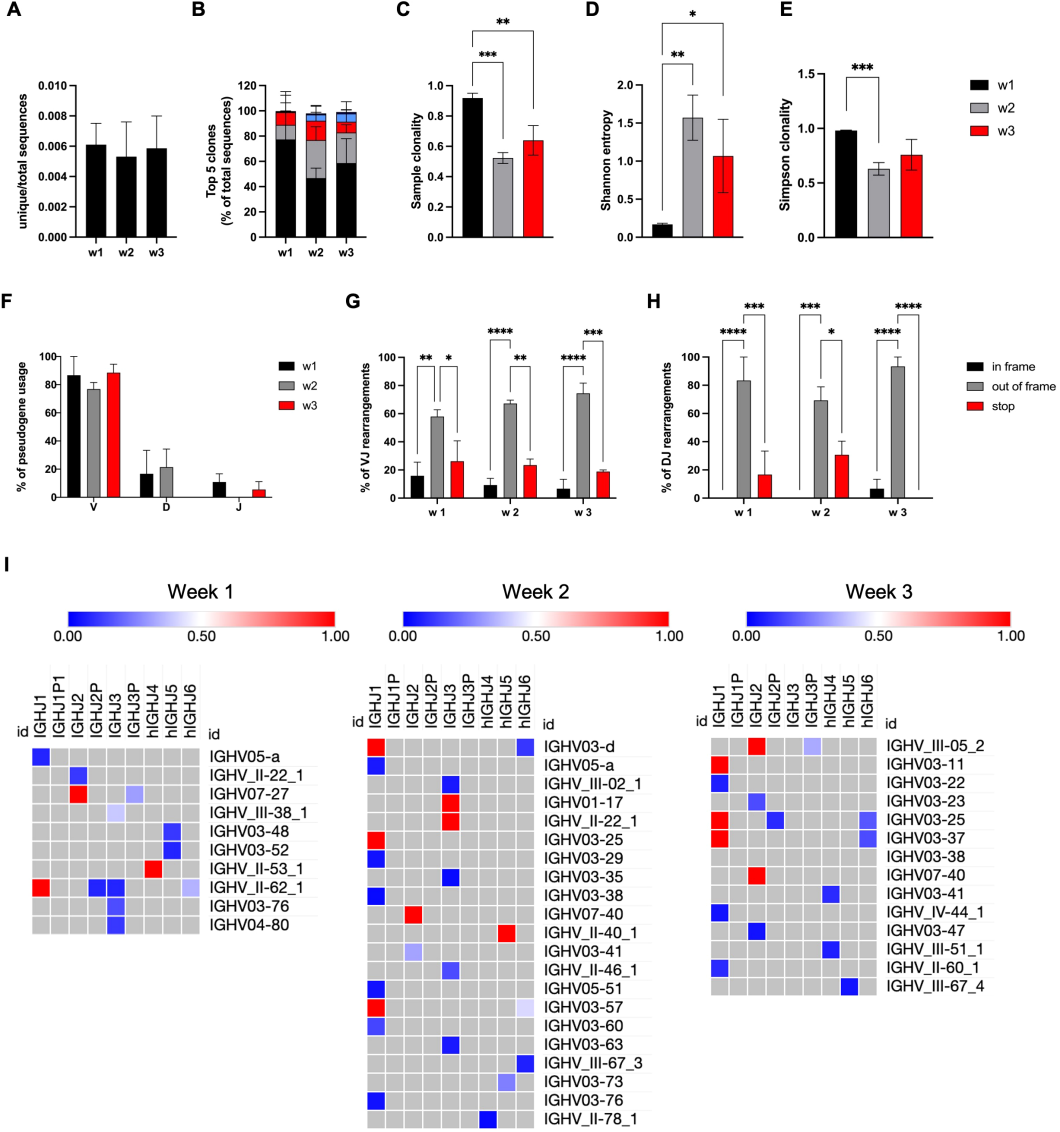
NK cells with RAG expression ontogeny have unproductive rearrangements in the IGH locus. GFP^+^ NK cells derived from RSS–EGFP^+/+^ iPSCs were sorted at weeks 1–3, and gDNA was isolated. **(A)** The ratio between the number of unique sequences and total sequences is shown for GFP^+^ NK cells obtained at weeks 1–3. **(B)** The frequency of the top five clones is shown as percentage of total sequences. The diversity of the IGH repertoire obtained from NK cells at weeks 1–3 was investigated using **(C)** sample clonality, **(D)** Shannon entropy, and **(E)** Simpson clonality. **(F)** The frequency of IGH V, D, and J pseudogene usage obtained from GFP^+^ NK cells at weeks 1–3 is shown as the percentage of total sequences. The amount of VJ **(G)** and DJ **(H)** rearrangements leading to in-frame or out-of-frame deletions or stop codons within the coding DNA is shown for all sequences obtained from NK cells at weeks 1–3. **(I)** Frequencies of VJ rearrangements in GFP^+^ NK cells obtained at weeks 1–3 are shown by color-coded heat maps. Shown are means ± SEM from three independent experiments. Statistical analysis was performed using two-way ANOVA, or unpaired t test, respectively (*p<0.05, **p<0.01, ***p<0.001, ****p<0.0001).

Up to 17 unique sequences and up to 6,825 total reads per sample were obtained for NK cells harvested at weeks 1–3 of differentiation. The mean ratios between unique and total sequences are shown for each time point in **Figure 4A**. As shown by the frequency of the top five clones (**Figure 4B**), the IGH repertoire was extremely clonal due to a few unique sequences. Further investigation of the diversity using indices, such as sample clonality, Shannon entropy, and Simpson clonality (**Figures 4C–E**), confirmed a clonal repertoire but increasing diversity in weeks 2 and 3. Importantly, none of the rearrangements found was productive. This was due to a high range of pseudogene usage (**Figure 4F**), or VJ and DJ rearrangements leading to out of frame sequences or stop codons within the open reading frame (**Figures 4G, H**). Clonal expansion is further indicated by the frequency of all VJ and DJ rearrangements as shown by heat maps (**Figure 4I**, **Supplementary Figure S10B**). The number of resolved V and D rearrangements found was only slightly higher than the number of unresolved sequences (**Supplementary Figure S10C**). There were no differences between the three time points regarding nucleotide losses at the joints (**Supplementary Figure S10D**) and N nucleotide additions (**Supplementary Figures S10E–G**). Despite low diversity, increased numbers of N1 and N2 nucleotide additions (>16 nt) were found in all samples (**Supplementary Figures S10F, G**), and a shifted distribution of the CDR3 length toward larger sequences (**Supplementary Figure S10H**) was observed.

We conclude from these findings that all IGH rearrangements found in NK cells and NK precursor cells are unproductive due to either pseudogene usage or disruption of the reading frame. Interestingly, rearrangements can be observed early (week 1) at differentiation, and the diversity of clones increases toward more mature NK cells.

### Differential gene expression between RAG-fate-mapped and non-mapped NK cells increases in the process of differentiation

**Figure 5:**
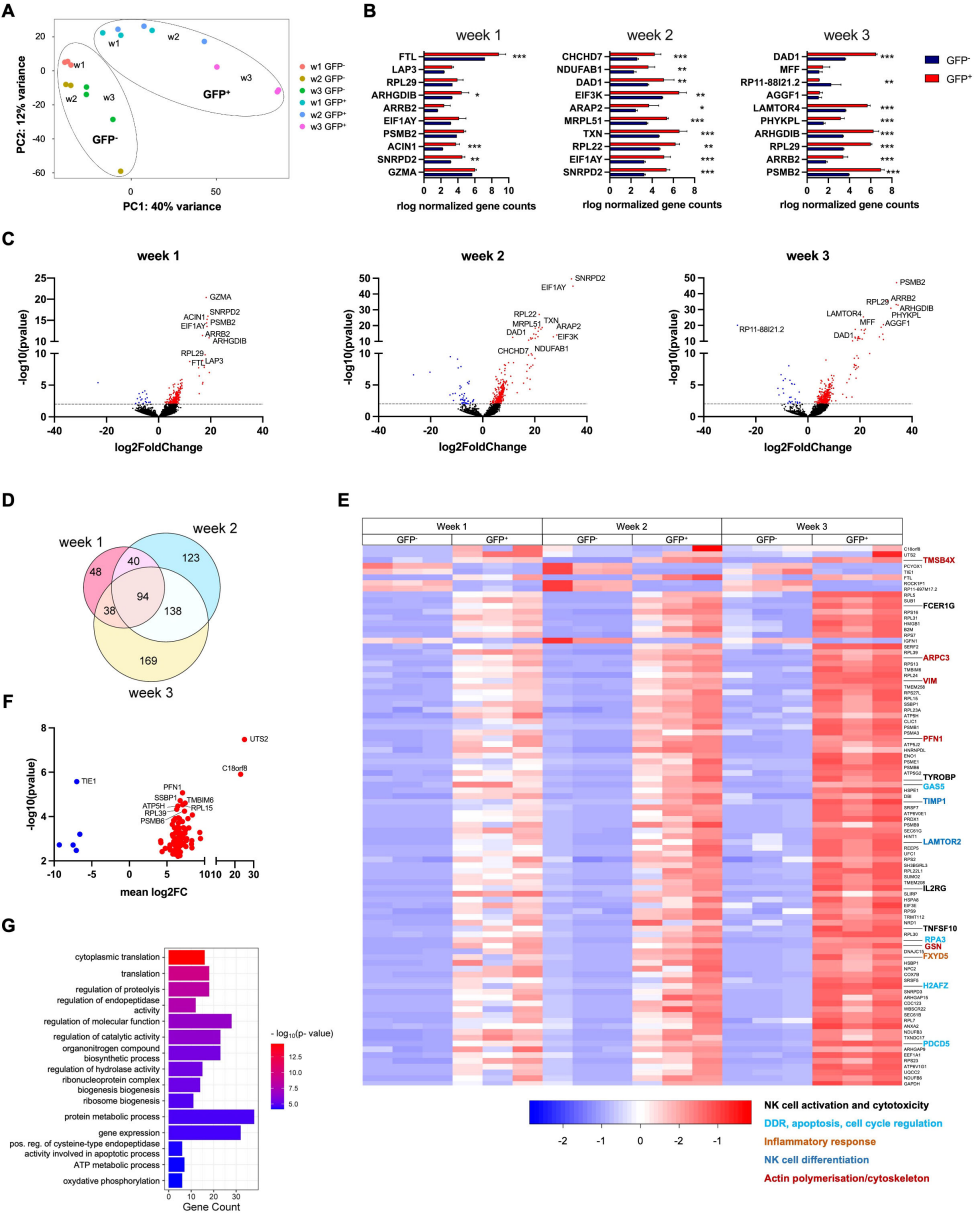
Differential gene expression observed in RAG-fate-mapped compared to non-mapped NK cells increases during the process of differentiation. RNA-Seq analysis was performed on GFP^−^ and GFP^+^ NK cells separated by fluorescence-activated cell sorting (FACS) at weeks 1–3. Three replicates were performed for each time point. **(A)** A principal component analysis was performed on all replicates obtained from GFP^−^ and GFP^+^ NK cells at weeks 1–3. Clusters of GFP^−^ and GFP^+^ NK cells are indicated by circles. **(B)** Normalized gene counts of GFP^−^ and GFP^+^ NK cells are shown for the top 10 differentially expressed genes (DEG p < 0.001). Statistics was calculated based on replicate values obtained from independent differentiation experiments using two-way ANOVA (*p<0.05, **p<0.01, ***p<0.001). **(C)** Volcano plots showing DE genes of GFP^−^ versus GFP^+^ NK cells obtained from weeks 1–3. The dotted line depicts the cutoff of differential expression (−log10[p-value] = 2, p-value <0.01). Downregulated genes are shown in blue, while upregulated genes are in red color. The 10 most differentially expressed genes are indicated by gene names. **(D)** Euler diagram showing the number of DE genes (p_adj_/DFR < 0.05) between GFP^−^ and GFP^+^ NK cells observed at weeks 1–3 and their overlap between these time points. **(E)** Heatmap visualizing the relative expression of genes differentially expressed at weeks 1–3. Shown are normalized gene counts from all replicates of GFP^−^ and GFP^+^ NK cells of each time point. Genes associated with NK cell development and function are highlighted and classified by the color-coded legend at the bottom. **(F)** Volcano plot depicting DE genes (p_adj_/DFR < 0.05) observed in GPP^+^/GFP^−^ NK cells at all three time points. Downregulated genes are shown in blue, while upregulated genes are in red color. The 10 most differentially expressed genes are indicated by gene names. **(G)** Gene enrichment analysis was performed on DE genes observed in NK cells at all time points (FDR < 0.05). Gene counts and FDR are shown for corresponding GO terms. (*p<0.05, **p<0.01, ***p<0.001).

Transcriptome analyses were performed using RNA-Seq on sorted GFP^−^ and GFP^+^ RSS–cEGFP^+/+^ NK cell precursors obtained at weeks 1, 2, and 3 of differentiation. Due to potential contamination with gDNA, mapped sequences were filtered to contain gapped alignments and exon–exon boundaries only (**Supplementary Figures S11A–C**). Principal component analysis (PCA) of the filtered RNA-Seq data on GFP^−^ versus GFP^+^ NK cells from weeks 1, 2, and 3 showed two different clusters that further separated in the process of differentiation (**Figure 5A**). In addition, a similarity analysis between all samples revealed a high distance between NK progenitors at weeks 1 and 3 (**Supplementary Figure S11D**). Accordingly, the number of differentially expressed (DE) genes increased over time from 220 at week 1, to 395 at week 2, and to 439 at week 3 (p_adj_ < 0.05). The normalized gene counts for GFP^−^ and GFP^+^ NK cells of the top 10 DE genes of each time point are shown in **Figure 5B**. The distribution of DE genes obtained from weeks 1, 2, and 3 is represented by volcano plots in **Figure 5C** indicating the top 10 DE genes.

There was a substantial overlap of DE genes at weeks 1, 2, and 3 (**Figure 5D**). In total, 94 genes that were differentially regulated at all time points could be identified. Gene expression was analyzed in this data set, and DE genes relevant for NK cell differentiation were identified (**Figure 5E**). The distribution of significantly up- and downregulated genes at all time points (p_adj_ < 0.05) is shown in **Figure 5F**, in which the top 10 DE genes are indicated. Of note, most of the DE genes were associated with transcription, translation, and protein processing.

The expression profiles of the top 100 DE genes observed at the individual time points (weeks 1–3) are shown in **Supplementary Figures S12A–C**.

Gene ontology (GO) analysis of biological processes performed on DE genes of weeks 1–3 (FDR < 0.05) revealed differential regulation of gene expression, translation, and metabolic processes (**Figure 5G**). The GO terms associated with gene expression and translation, metabolism, and cellular function found to be enriched in NK progenitors obtained from week 1 to 3 are shown in **Supplementary Figures S12D–F**.

### DE genes of RAG-fate-mapped NK progenitors are associated with NK cell maturation and cytotoxicity

**Figure 6:**
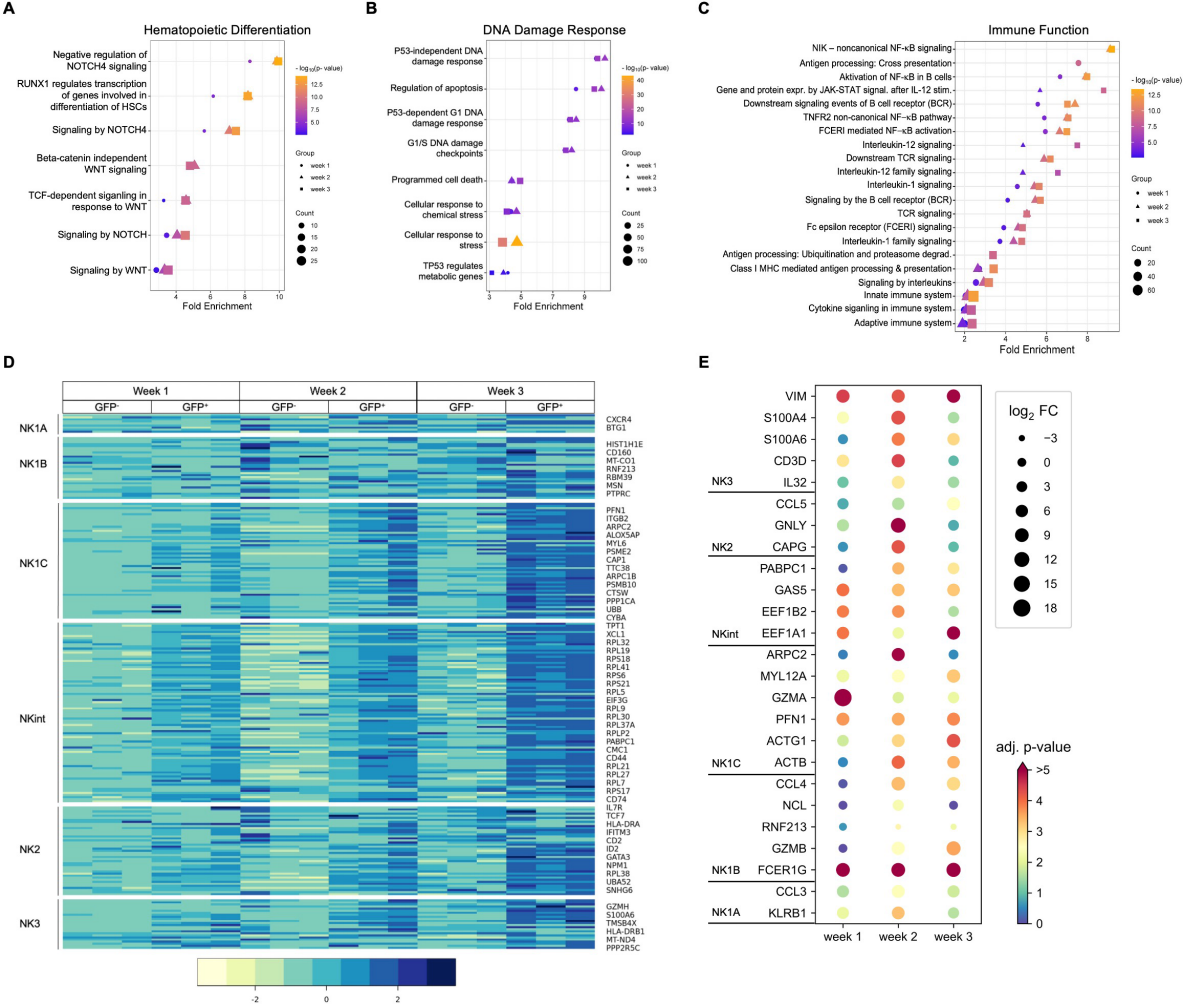
Gene expression profiles observed in RAG-fate-mapped NK progenitors indicate increased maturation and cytotoxicity. Selected pathways induced by differentially expressed genes (FDR < 0.05) associated with hematopoietic differentiation **(A)**, DNA damage response **(B)**, and immune function **(C)** are shown for NK cells from week 1–3. Corresponding fold enrichment is shown on the x-axis, whereas different time points are coded by symbols, fold change by symbol size, and the adjusted p-value (−log10[p-value]) by color as indicated. **(D)** Heatmap depicting relative expression of genes defining NK cell subgroups in GFP^+^ and GFP^−^ progenitors obtained at indicated time points. Shown are normalized gene counts for each replicate and time point color coded as indicated by the legend on the right. NK cell populations are indicated on the left. **(E)** Differential expression of genes defining relevant NK cell subgroups in GFP^+^/GFP^−^ NK cells at indicated time points are presented in a dot plot diagram. Differential expression (p_adj_/FDR < 0.05) has been observed at least at one time point (weeks 1, 2, and 3). The log2 fold change (FC) is depicted by dot size; corresponding adjusted p-values (−log10[p-value]) are color coded as indicated.

Pathway analysis using Reactome showed differences between RAG-fate-mapped and non-mapped NK cells regarding hematopoietic differentiation, DDR, and immune function (**Figures 6A–C**). Differentially regulated pathways associated with hematopoietic differentiation were concerned with NOTCH signaling and Wnt signaling (**Figure 6A**), pathways associated with DDR were predominantly related to p53 expression and apoptosis (**Figure 6B**), and pathways involved in immune function were associated with TCR, BCR, and cytokine signaling (**Figure 6C**). Pathways associated with hematopoietic differentiation and immune function in NK progenitors were increasingly upregulated in the process of differentiation from week 1 to 3 (**Figures 6A, C**, **Supplementary Table S2**).

Six NK cell populations characterized by differential maturity and functional properties were recently classified using high-dimensional single-cell RNA sequencing (33). Gene clusters assigned to specific NK cell subgroups were not differentially regulated in RAG-fate-mapped (GFP^+^) and non-mapped (GFP^−^) NK cells. However, some genes related to the NK1B population were upregulated in GFP^−^ NK cells, whereas GFP^+^ NK cells expressed genes associated with the more mature NK1C, NK intermediates (NKint), and NK2 and NK3 populations (**Figures 6D, E**).

**Figure 7:**
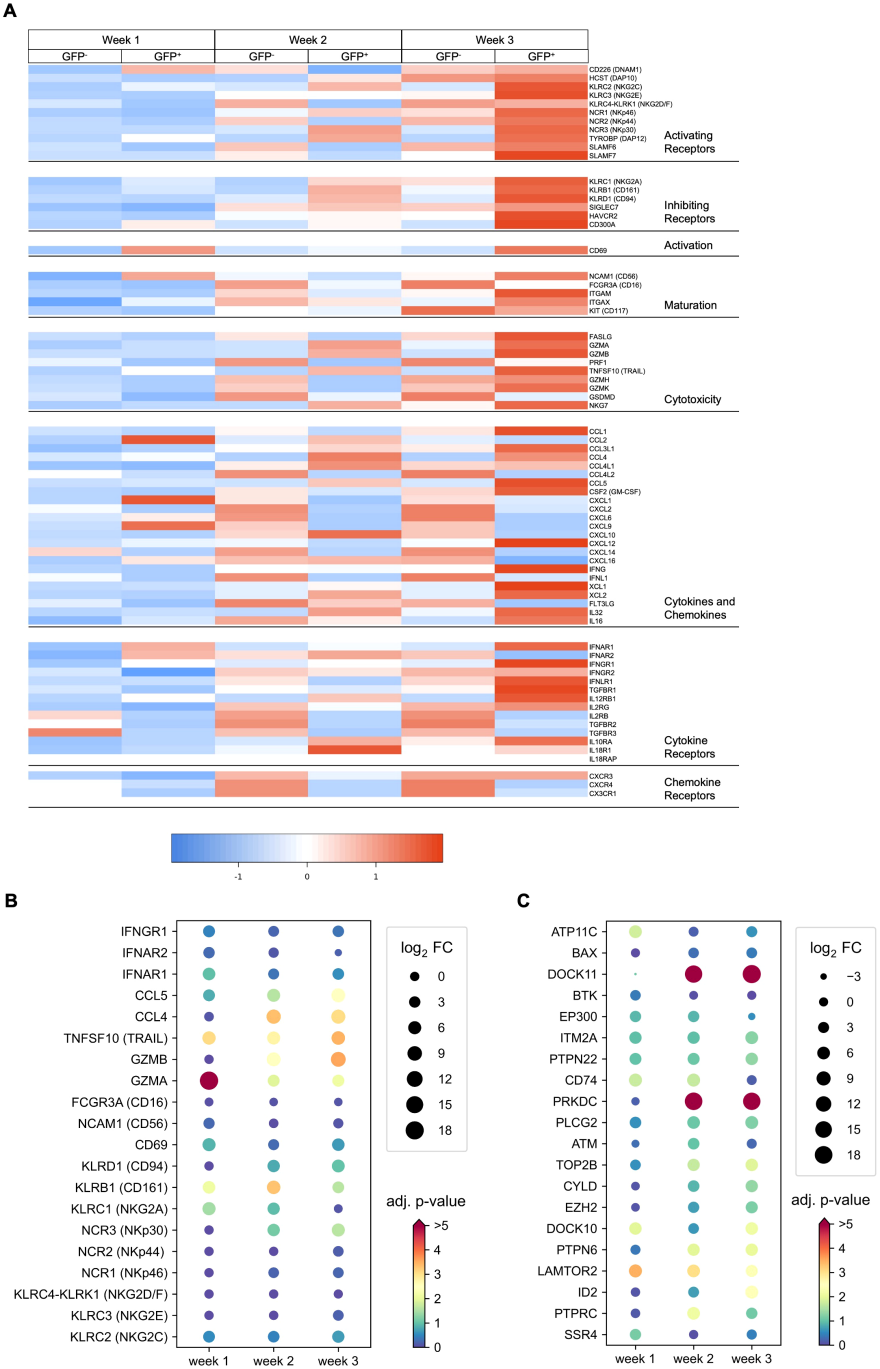
DE genes observed in RAG-fate-mapped NK cells are associated with NK cell maturation and function and have potential overlap with B lineage differentiation. **(A)** Heatmap showing the mean normalized expression of genes grouped by categories of activating and inhibiting receptors, cytotoxic molecules, cytokines, chemokines, and cytokine/chemokine receptors in GFP^−^ and GFP^+^ NK cells obtained at weeks 1–3. **(B)** Dot plot diagram depicting the log2 fold change (FC) and corresponding adjusted p-value (−log10[p-value]) of selected genes involved in NK cell maturation and function. **(C)** Dot plot diagram depicting the log2 fold change (FC) and corresponding adjusted p-value (−log10[p-value]) of the top 20 highly expressed genes associated with B and NK cell differentiation.

Expression of genes associated with NK cell differentiation and function were selectively analyzed in the dataset. Means of normalized gene counts obtained from GFP^−^ and GFP^+^ NK cells at weeks 1–3 are visualized by heatmap, and grouped into categories of genes encoding activating and inhibiting receptors, proteins related to activation, maturity, cytotoxicity, cytokines, chemokines, as well as cytokine and chemokine receptors (**Figure 7A**). Expression of most genes increased during the time of differentiation from week 1–3, which was particularly observed in genes encoding for NK lymphocyte and cytokine receptors.

Differential expression is shown for selected genes in **Figures 7B**; **Supplementary Figure S12A**. In contrast to *GZMA* (granzyme A), *GZMB* (granzyme B), and *PRF1* (perforin) that mediate cellular cytotoxicity by forming pores in the target cell membrane, *FASLG* and *TNFSF10* are involved in receptor-mediated apoptosis induced by NK cells. However, only *GZMA*, *GZMB*, and *TNFSF10* were found to be significantly upregulated in GFP^+^ NK cells (**Figures 7A, B**, **Supplementary Figure S13A**). In contrast to the expression of genes coding for several chemokines and chemokine receptors that were downregulated in RAG-fate-mapped NK cells, significantly upregulated gene expression could be observed for cytokines such as CCL4 and CCL5.

Although the differentiation potential of the NK cell subsets investigated in this study is heterogeneous, RAG-fate-mapped NK cells show a tendency toward increased maturation and cytotoxicity.

### Gene expression associated with B-cell differentiation and V(D)J recombination can be detected in RAG-fate-mapped NK cells

Genes associated with B (GO_0030183) and NK lymphocyte (GO_0001779) development were identified using Ensembl Biomart, and gene expression was analyzed in GFP^−^ and GFP^+^ NK progenitors form week 1–3 (**Supplementary Figure S13B**). In total, 134 genes could be identified in this dataset, of which 13 were associated with NK-cell and 121 with B-cell differentiation. Differential expression is shown for the top 20 highly expressed genes (**Figure 7C**), including 7 DE genes (*PTPN6*, *DOCK11*, *PRKDC*, *ID2*, *DOCK10*, *LAMTOR2*, *IL2RG).* Highly significant were the differential expressions of *PRKDC* and *DOCK11. PRKDC* encodes for DNA-dependent protein kinase, catalytic subunit (DNA-PKcs), a key regulator of NHEJ and V(D)J recombination, and DDR processes (34). *DOCK11* is associated with early B-cell development in the bone marrow and germinal center formation with some redundancy with *DOCK10* (35, 36). None of the genes have been associated with NK-cell differentiation before.

In summary, expression of genes associated with B-cell development can be detected in iPSC-derived NK cells, of which *PRKDC* is differentially expressed in RAG-fate-mapped NK cells.

## Discussion

In this study, we demonstrate that recombination of V(D)J gene elements initiated by RAG1 and RAG2 endonucleases occurs in human NK cell development and impacts maturation and function. We placed a reporter construct consisting of an inverted EGFP cassette flanked by two RSS target sites into human iPSCs. During further differentiation toward NK lymphocytes *in vitro*, NK precursors with RAG-fate ontogeny could subsequently be investigated regarding their maturity, functional properties, and DNA damage response.

GFP expression could be observed in up to 14% of NK cells as early as day 3 of differentiation. The observation that RAG1 and RAG2 expression occurs in waves at multiple time points has been reported previously (37). Interestingly, RAG-fate ontogeny was found in a distinct mature and activated NK cell population, which was further characterized by alternate CD45 isotype expression. Whereas the majority of GFP^−^ NK cells expressed CD45^bright^/CD45RA, CD45^dim^/CD45RO/RB expression was found on RAG-fate-mapped NK cells.

CD45 is a receptor-linked tyrosine phosphatase that modulates signal transduction of lymphocyte and cytokine receptors (38). Alternative splicing of exons 4–6 (A, B, C), which is highly conserved and strictly regulated among species, results in eight isotypes (RABC, RAB, RAC, RBC. RA, RB, RC, RO) that differ by extracellular glycosylation (39). The length of the extracellular molecules impacts the tendency to homodimerize and thereby modulates the signal strength of CD45 (40). The low-molecular weight isoform CD45RO is associated with the highest rate of dimerization and transduces a weaker signal. The isotype switching is necessary for the termination of a T-cell response and a differentiation from naïve (CD45RA^+^) to memory (CD45RO^+^) T cells (40). CD45RB discriminates two distinct memory populations in CD4^+^ (41) and CD8^+^ T cells (42) and defines a population of terminally differentiated B cells (43). Increased fractions of CD45^dim^ and CD45RO^+^ NK cells have been reported in patients with malignancies and severe infections (44–46), whereas CD45RO^+^RA^+^ NK cells were characterized by a higher anti-tumor activity.

RAG-fate-mapped (GFP^+^) NK cells could be characterized as CD56^dim^ compared to GFP^−^CD56^bright^ cells. CD56^bright^CD16^−^ are considered as immature precursors of CD56^dim^CD16^+^ NK cells that develop in secondary lymphoid organs and present the most abundant population in peripheral blood (2). Compared to NK cells developed *in vivo*, iPSC-derived NK cells may express CD16 at lower frequencies (19, 47). According to surface marker expression and functional studies, RAG-fate-mapped NK cells displayed a more mature and activated phenotype shown by increased expression of CD57, CXCR1, DNAM1, and NK-p44, as well as increased degranulation and IFNγ response. Although to a low extent, NKG2C and KIR2DL2/3 were only found on RAG-fate-mapped NK cells. In summary, RAG-fate-mapped NK cells share features of adaptive NK cells (1).

Transfection of additional RAG1 and RAG2 mRNA into HSPCs resulted in an extended number GFP^+^ NK cells and contrasted the differential phenotype observed between RAG-fate-mapped and non-mapped NK cells. However, RAG1 and RAG2 mRNA increased the targeting rate of the reporter cassette to no more than 21.4%. Similar observations of incomplete recombination, despite high transfection efficiency, were made in other cellular models using the same reporter cassette (22, 48). We conclude that RAG-expression ontogeny does not imply recombination on the BCR or TCR loci. The reporter cassette we are using in this study distinguishes cells with recombination rather than RAG1 or RAG2 expression in their ontogeny, although it is historically defined as a “RAG-fate mapping” reporter. Therefore, RAG-expression ontogeny without recombination cannot be excluded in GFP^−^ cells, as well as increased loss of GFP^+^ in the process of differentiation due to poor survival.

Although both Ig and TCR rearrangements have been reported in peripheral blood NK cells (13–15), TCR rearrangements could not be observed by PCR in our study. This could be related to the iPSC line used or our differentiation protocol not supporting T-cell differentiation, as well as the lower sensitivity of end-point PCR. The genomic IGH repertoire was studied by NGS and revealed unproductive rearrangements due to pseudogene usage or disruption of the reading frame. The repertoire was characterized by limited diversity, clonal expansion, and a CDR3 length shifted to larger sequences as reported in unproductive IGH repertoires (49).

Transcription profiles studied by RNA-Seq showed a more mature and activated phenotype in RAG-fate-mapped NK cells indicated by upregulation of *TYROPB* (DAP12)*, FCER1G* (FcRγ), *GZMA*, *GZMB*, and *TNSF10*. *TNFSF10*, associated with NK cell-mediated cytotoxicity, was increasingly upregulated in GFP^+^ cells in weeks 1–3. *FCER1G* and *TYROPB* both encode adaptor proteins containing immunoreceptor tyrosine-based activation motifs (ITAM) that transmit activating signals of NK cell surface receptors. DNAX-activating protein of 12 kDa (DAP12), also known as tyrosine kinase-binding protein (TYROPB), is involved in signal transduction of KIR-S and NKp44 (50, 51), whereas FcRγ (encoded by *FCER1G*) plays a role in KIR2DL4 activation (52). The differential phenotype of GFP^−^ compared to GFP^+^ NK progenitor cells became further apparent in the process of differentiation. There was a substantial overlap of DE genes at all time points and between replicates confirming the consistency of the study. In addition to genes associated with ribosomal and transcriptional proteins, mTOR-regulating genes (*FKBP3* and *LAMTOR2*) were highly enriched in RAG-fate-mapped NK cells. *LAMTOR2* plays an important role in NK cell development as regulator of the mTOR signaling pathway (53). This goes in line with an increased degranulation response and cytokine production. Furthermore, *IL2RG* codes for the signaling subunit of several cytokine receptors (common γ chain), of which IL-2, IL-7, and IL-15 are mandatory for NK cell development (54). GAS5, RPA3, H2AFZ, and PDCD5 are associated with replication, DDR, apoptosis, and cell cycle regulation (55–58). FXYD5 is a Na^+^/K^+^ ATPase involved in the regulation of inflammatory responses and production of cytokines and chemokines (59). *TIMP1* encodes for a tissue inhibitor of metalloprotease protein (TIMP) TIMP1. TIMP1 and TIMP2 counteract transforming growth factor beta (TGFβ)-induced polarization of cytolytic decidual NK cells normalizing cytotoxic function and revoking the pro-angiogenic commitment of NK cells (60). In addition, upregulation of genes associated with actin polarization and cytoskeleton (*TMSB4X*, *ARPC3*, *VIM*, *PFN1*, *GSN*) was observed in RAG-fate-mapped NK cells. Of note, these observations were not validated by functional analyses.

A PCA performed on RNA-Seq data obtained from GFP^−^ versus GFP^+^ NK cells at weeks 1–3 showed two different clusters that further separated in the process of differentiation. This correlates with the differential phenotype of NK lineage markers expressed at the cell surface and the increasing number of DEGs observed at week 1 compared to weeks 2 and 3.

GO enrichment and pathway analysis of expression profiles revealed differential regulation of stem cell differentiation, DDR, and cytokine signaling. A discrimination of definitive and primitive hematopoiesis by the Wnt signaling pathway has been described by several groups (61, 62). In contrast to adaptive lymphocytes arising from definitive precursors, NK cells can derive from both. The upregulation of apoptosis pathways may result in the reduced DDR capacity observed in RAG-fate-mapped NK cells.

Comparing bulk RNA-Seq data generated in this study with single-cell RNA-Seq analyses obtained from PBMCs (33) suggested increased maturity in RAG-fate-mapped NK cells, which could not be categorized into any cluster of defined NK cell populations. We conclude from this limited comparison that iPSC-derived NK cells are more immature than peripheral blood NK cells.

In a mouse model using RAG-fate-mapped lymphocytes, Rag-deficient NK cells presented a hyperresponsive phenotype with mature (KLRG1^hi^), activated (CD69^hi^, CD62L^lo^), and cytotoxic NK cells, but at the same time reduced cellular fitness marked by increased susceptibility to virus-driven proliferation (16). In contrast to findings in mice, an immature phenotype was reported in NK cells obtained from RAG1-, RAG2-, and ARTEMIS-deficient patients characterized by an increased proportion of CD56^bright^CD16^−^/CD56^bright^CD16^int^ NK cells, expression of NKG2A, and increased cytotoxicity, while the proportion of mature CD57^+^CD16^hi^CXCR1^+^ NK cells was reduced (17). Interestingly, NK cell immaturity correlated with clinical severity of immunodeficiency, since patients presenting with hypomorphic diseases seemed to be less affected. The maturation defect reported in this cohort could possibly be related to the inability of NK cells obtained from patients with RAG or NHEJ deficiencies to perform V(D)J recombination on their Ig or TCR loci.

In contrast to observations made in mice, where murine murine Rag1^−/−^ NK cells accumulated DNA double-strand breaks (DSBs) in response to ionizing radiation (16), DDR capacity was severely impaired in human RAG-fate-mapped NK cells generated in our study. Ionizing radiation resulted in increased levels of γH2AX suggesting unrepaired DNA damage that correlated with poor survival. This observation may be related to the different cellular metabolism in this more mature NK cell population rather than being caused by RAG expression or recombination itself. Phenotypes of exhaustion, anergy, and senescence have been described in aged NK cells and are associated with reduced effector function, downregulation of activating receptors, and increased pro-inflammatory secretions in senescent cells (63), which have not been observed in RAG-fate-mapped NK cells.

In summary, our study characterizes RAG-fate-mapped NK cells with advanced maturation and activation but reduced cellular fitness compared to NK cells lacking this ontogeny. In addition, the distinct CD45RO/CD45RB isotype expression suggests a memory-like and activated phenotype. However, the question remains whether NK cells carrying unproductive rearrangements of V(D)J elements on Ig and TCR loci are committed to a specific function, or whether they are a by-product of unsuccessful T- or B-lymphocyte development reprogrammed to NK cells.

## Data Availability

The datasets presented in this study can be found in online repositories. The names of the repository/repositories and accession number(s) can be found below: https://www.ncbi.nlm.nih.gov/geo/, GSE283274 (64) https://clients.adaptivebiotech.com/pub/sprissler-2024-s, DOI 10.21417/JS2024S.

## References

[1] Freud AG Mundy-Bosse BL Yu J Caligiuri MA. The broad spectrum of human natural killer cell diversity. Immunity. (2017) 47:820–33. doi: 10.1016/j.immuni.2017.10.008, PMID: 29166586 PMC5728700

[2] Freud AG Yu J Caligiuri MA. Human natural killer cell development in secondary lymphoid tissues. Semin Immunol. (2014) 26:132–7. doi: 10.1016/j.smim.2014.02.008, PMID: 24661538 PMC4010312

[3] Pegram HJ Andrews DM Smyth MJ Darcy PK Kershaw MH. Activating and inhibitory receptors of natural killer cells. Immunol Cell Biol. (2011) 89:216–24. doi: 10.1038/icb.2010.78, PMID: 20567250

[4] Doulatov S Notta F Eppert K Nguyen LT Ohashi PS Dick JE. Revised map of the human progenitor hierarchy shows the origin of macrophages and dendritic cells in early lymphoid development. Nat Immunol. (2010) 11:585–93. doi: 10.1038/ni.1889, PMID: 20543838

[5] Adolfsson J Mansson R Buza-Vidas N Hultquist A Liuba K Jensen CT. Identification of Flt3+ lympho-myeloid stem cells lacking erythro-megakaryocytic potential a revised road map for adult blood lineage commitment. Cell. (2005) 121:295–306. doi: 10.1016/j.cell.2005.02.013, PMID: 15851035

[6] Lai AY Kondo M. Asymmetrical lymphoid and myeloid lineage commitment in multipotent hematopoietic progenitors. J Exp Med. (2006) 203:1867–73. doi: 10.1084/jem.20060697, PMID: 16880261 PMC2118384

[7] Phillips JH Hori T Nagler A Bhat N Spits H Lanier LL. Ontogeny of human natural killer (NK) cells: fetal NK cells mediate cytolytic function and express cytoplasmic CD3 epsilon,delta proteins. J Exp Med. (1992) 175:1055–66. doi: 10.1084/jem.175.4.1055, PMID: 1372642 PMC2119193

[8] Rodewald HR Moingeon P Lucich JL Dosiou C Lopez P Reinherz EL. A population of early fetal thymocytes expressing Fc gamma RII/III contains precursors of T lymphocytes and natural killer cells. Cell. (1992) 69:139–50. doi: 10.1016/0092-8674(92)90125-v, PMID: 1532536

[9] Carlyle JR Michie AM Furlonger C Nakano T Lenardo MJ Paige CJ. Identification of a novel developmental stage marking lineage commitment of progenitor thymocytes. J Exp Med. (1997) 186:173–82. doi: 10.1084/jem.186.2.173, PMID: 9221746 PMC2198984

[10] Bezman NA Kim CC Sun JC Min-Oo G Hendricks DW Kamimura Y. Molecular definition of the identity and activation of natural killer cells. Nat Immunol. (2012) 13:1000–9. doi: 10.1038/ni.2395, PMID: 22902830 PMC3572860

[11] Notta F Doulatov S Laurenti E Poeppl A Jurisica I Dick JE. Isolation of single human hematopoietic stem cells capable of long-term multilineage engraftment. Science. (2011) 333:218–21. doi: 10.1126/science.1201219, PMID: 21737740

[12] Doulatov S Notta F Laurenti E Dick JE. Hematopoiesis: a human perspective. Cell Stem Cell. (2012) 10:120–36. doi: 10.1016/j.stem.2012.01.006, PMID: 22305562

[13] Borghesi L Hsu LY Miller JP Anderson M Herzenberg L Herzenberg L. B lineage-specific regulation of V(D)J recombinase activity is established in common lymphoid progenitors. J Exp Med. (2004) 199:491–502. doi: 10.1084/jem.20031800, PMID: 14769852 PMC2211824

[14] Fronkova E Krejci O Kalina T Horvath O Trka J Hrusak O. Lymphoid differentiation pathways can be traced by TCR delta rearrangements. J Immunol. (2005) 175:2495–500. doi: 10.4049/jimmunol.175.4.2495, PMID: 16081821

[15] Pilbeam K Basse P Brossay L Vujanovic N Gerstein R Vallejo AN. The ontogeny and fate of NK cells marked by permanent DNA rearrangements. J Immunol. (2008) 180:1432–41. doi: 10.4049/jimmunol.180.3.1432, PMID: 18209038 PMC4465768

[16] Karo JM Schatz DG Sun JC. The RAG recombinase dictates functional heterogeneity and cellular fitness in natural killer cells. Cell. (2014) 159:94–107. doi: 10.1016/j.cell.2014.08.026, PMID: 25259923 PMC4371485

[17] Dobbs K Tabellini G Calzoni E Patrizi O Martinez P Giliani SC. Natural killer cells from patients with recombinase-activating gene and non-homologous end joining gene defects comprise a higher frequency of CD56(bright) NKG2A(+++) cells, and yet display increased degranulation and higher perforin content. Front Immunol. (2017) 8:798. doi: 10.3389/fimmu.2017.00798, PMID: 28769923 PMC5511964

[18] Bredemeyer AL Sharma GG Huang CY Helmink BA Walker LM Khor KC. ATM stabilizes DNA double-strand-break complexes during V(D)J recombination. Nature. (2006) 442:466–70. doi: 10.1038/nature04866, PMID: 16799570

[19] Euchner J Sprissler J Cathomen T Furst D Schrezenmeier H Debatin KM. Natural killer cells generated from human induced pluripotent stem cells mature to CD56(bright)CD16(+)NKp80(+/-)*In-vitro* and express KIR2DL2/DL3 and KIR3DL1. Front Immunol. (2021) 12:640672. doi: 10.3389/fimmu.2021.640672, PMID: 34017328 PMC8129508

[20] Chen Y Cao J Xiong M Petersen AJ Dong Y Tao Y. Engineering human stem cell lines with inducible gene knockout using CRISPR/cas9. Cell Stem Cell. (2015) 17:233–44. doi: 10.1016/j.stem.2015.06.001, PMID: 26145478 PMC4530040

[21] Chen Y Xiong M Dong Y Haberman A Cao J Liu H. Chemical control of grafted human PSC-derived neurons in a mouse model of parkinson’s disease. Cell Stem Cell. (2016) 18:817–26. doi: 10.1016/j.stem.2016.03.014, PMID: 27133795 PMC4892985

[22] Felgentreff K Lee YN Frugoni F Du L van der Burg M Giliani S. Functional analysis of naturally occurring DCLRE1C mutations and correlation with the clinical phenotype of ARTEMIS deficiency. J Allergy Clin Immunol. (2015) 136:140–50 e7. doi: 10.1016/j.jaci.2015.03.005, PMID: 25917813 PMC4494888

[23] van Dongen JJ Langerak AW Bruggemann M Evans PA Hummel M Lavender FL. Design and standardization of PCR primers and protocols for detection of clonal immunoglobulin and T-cell receptor gene recombinations in suspect lymphoproliferations: report of the BIOMED-2 Concerted Action BMH4-CT98-3936. Leukemia. (2003) 17:2257–317. doi: 10.1038/sj.leu.2403202, PMID: 14671650

[24] Gene Ontology C Aleksander SA Balhoff J Carbon S Cherry JM Drabkin HJ. The gene ontology knowledgebase in 2023. Genetics. (2023) 224(1):iyad031. doi: 10.1093/genetics/iyad031doi: 10.1093/genetics/iyad031, PMID: 36866529 PMC10158837

[25] Thomas PD Ebert D Muruganujan A Mushayahama T Albou LP Mi H. PANTHER: Making genome-scale phylogenetics accessible to all. Protein Sci. (2022) 31:8–22. doi: 10.1002/pro.4218, PMID: 34717010 PMC8740835

[26] Ashburner M Ball CA Blake JA Botstein D Butler H Cherry JM. Gene ontology: tool for the unification of biology. The Gene Ontology Consortium. Nat Genet. (2000) 25:25–9. doi: 10.1038/75556, PMID: 10802651 PMC3037419

[27] Galaxy C. The Galaxy platform for accessible, reproducible and collaborative biomedical analyses: 2022 update. Nucleic Acids Res. (2022) 50:W345–W51. doi: 10.1093/nar/gkac610, PMID: 35446428 PMC9252830

[28] Tang D Chen M Huang X Zhang G Zeng L Zhang G. SRplot: A free online platform for data visualization and graphing. PloS One. (2023) 18:e0294236. doi: 10.1371/journal.pone.0294236, PMID: 37943830 PMC10635526

[29] Saito Y Sugimoto C Mituyama T Wakao H. Epigenetic silencing of V(D)J recombination is a major determinant for selective differentiation of mucosal-associated invariant t cells from induced pluripotent stem cells. PloS One. (2017) 12:e0174699. doi: 10.1371/journal.pone.0174699, PMID: 28346544 PMC5367832

[30] Dawes R Petrova S Liu Z Wraith D Beverley PC Tchilian EZ. Combinations of CD45 isoforms are crucial for immune function and disease. J Immunol. (2006) 176:3417–25. doi: 10.4049/jimmunol.176.6.3417, PMID: 16517710 PMC2619577

[31] Barrow AD Martin CJ Colonna M. The natural cytotoxicity receptors in health and disease. Front Immunol. (2019) 10:909. doi: 10.3389/fimmu.2019.00909, PMID: 31134055 PMC6514059

[32] Bryceson YT March ME Ljunggren HG Long EO. Synergy among receptors on resting NK cells for the activation of natural cytotoxicity and cytokine secretion. Blood. (2006) 107:159–66. doi: 10.1182/blood-2005-04-1351, PMID: 16150947 PMC1895346

[33] Rebuffet L Melsen JE Escaliere B Basurto-Lozada D Bhandoola A Bjorkstrom NK. High-dimensional single-cell analysis of human natural killer cell heterogeneity. Nat Immunol. (2024) 25(8):1474–88. doi: 10.1038/s41590-024-01883-0, PMID: 38956378 PMC11291291

[34] van der Burg M van Dongen JJ van Gent DC. DNA-PKcs deficiency in human: long predicted, finally found. Curr Opin Allergy Clin Immunol. (2009) 9:503–9. doi: 10.1097/ACI.0b013e3283327e41, PMID: 19823081

[35] Matsuda T Yanase S Takaoka A Maruyama M. The immunosenescence-related gene Zizimin2 is associated with early bone marrow B cell development and marginal zone B cell formation. Immun Ageing. (2015) 12:1. doi: 10.1186/s12979-015-0028-x, PMID: 25729399 PMC4343071

[36] Block J Rashkova C Castanon I Zoghi S Platon J Ardy RC. Systemic inflammation and normocytic anemia in DOCK11 deficiency. N Engl J Med. (2023) 389:527–39. doi: 10.1056/NEJMoa2210054, PMID: 37342957

[37] Grawunder U Schatz DG Leu TM Rolink A Melchers F. The half-life of RAG-1 protein in precursor B cells is increased in the absence of RAG-2 expression. J Exp Med. (1996) 183:1731–7. doi: 10.1084/jem.183.4.1731, PMID: 8666930 PMC2192496

[38] Penninger JM Irie-Sasaki J Sasaki T Oliveira-dos-Santos AJ. CD45: new jobs for an old acquaintance. Nat Immunol. (2001) 2:389–96. doi: 10.1038/87687, PMID: 11323691

[39] Rogers PR Pilapil S Hayakawa K Romain PL Parker DC. CD45 alternative exon expression in murine and human CD4+ T cell subsets. J Immunol. (1992) 148:4054–65. doi: 10.4049/jimmunol.148.12.4054, PMID: 1351092

[40] Xu Z Weiss A. Negative regulation of CD45 by differential homodimerization of the alternatively spliced isoforms. Nat Immunol. (2002) 3:764–71. doi: 10.1038/ni822, PMID: 12134145

[41] Horgan KJ Tanaka Y Luce GE van Seventer GA Nutman TB Shaw S. CD45RB expression defines two interconvertible subsets of human CD4+ T cells with memory function. Eur J Immunol. (1994) 24:1240–3. doi: 10.1002/eji.1830240536, PMID: 7910140

[42] Krummey SM Morris AB Jacobs JR McGuire DJ Ando S Tong KP. CD45RB status of CD8(+) T cell memory defines T cell receptor affinity and persistence. Cell Rep. (2020) 30:1282–91 e5. doi: 10.1016/j.celrep.2020.01.016, PMID: 32023448 PMC7155808

[43] Koers J Pollastro S Tol S Pico-Knijnenburg I Derksen NIL van Schouwenburg PA. CD45RB glycosylation and ig isotype define maturation of functionally distinct B cell subsets in human peripheral blood. Front Immunol. (2022) 13:891316. doi: 10.3389/fimmu.2022.891316, PMID: 35572548 PMC9095956

[44] Fu X Liu Y Li L Li Q Qiao D Wang H. Human natural killer cells expressing the memory-associated marker CD45RO from tuberculous pleurisy respond more strongly and rapidly than CD45RO- natural killer cells following stimulation with interleukin-12. Immunology. (2011) 134:41–9. doi: 10.1111/j.1365-2567.2011.03464.x, PMID: 21711347 PMC3173693

[45] Krzywinska E Allende-Vega N Cornillon A Vo DN Cayrefourcq L Panabieres C. Identification of anti-tumor cells carrying natural killer (NK) cell antigens in patients with hematological cancers. EBioMedicine. (2015) 2:1364–76. doi: 10.1016/j.ebiom.2015.08.021, PMID: 26629531 PMC4634619

[46] Krzywinska E Cornillon A Allende-Vega N Vo DN Rene C Lu ZY. CD45 isoform profile identifies natural killer (NK) subsets with differential activity. PloS One. (2016) 11:e0150434. doi: 10.1371/journal.pone.0150434, PMID: 27100180 PMC4839597

[47] Cichocki F Bjordahl R Gaidarova S Mahmood S Abujarour R Wang H. iPSC-derived NK cells maintain high cytotoxicity and enhance *in vivo* tumor control in concert with T cells and anti-PD-1 therapy. Sci Transl Med. (2020) 12(568):eaaz5618. doi: 10.1126/scitranslmed.aaz5618, PMID: 33148626 PMC8861807

[48] Lee YN Frugoni F Dobbs K Walter JE Giliani S Gennery AR. A systematic analysis of recombination activity and genotype-phenotype correlation in human recombination-activating gene 1 deficiency. J Allergy Clin Immunol. (2014) 133:1099–108. doi: 10.1016/j.jaci.2013.10.007, PMID: 24290284 PMC4005599

[49] Larimore K McCormick MW Robins HS Greenberg PD. Shaping of human germline IgH repertoires revealed by deep sequencing. J Immunol. (2012) 189:3221–30. doi: 10.4049/jimmunol.1201303, PMID: 22865917

[50] Tomasello E Vivier E. KARAP/DAP12/TYROBP: three names and a multiplicity of biological functions. Eur J Immunol. (2005) 35:1670–7. doi: 10.1002/eji.200425932, PMID: 15884055

[51] Medjouel Khlifi H Guia S Vivier E Narni-Mancinelli E. Role of the ITAM-bearing receptors expressed by natural killer cells in cancer. Front Immunol. (2022) 13:898745. doi: 10.3389/fimmu.2022.898745, PMID: 35757695 PMC9231431

[52] Kikuchi-Maki A Catina TL Campbell KS. Cutting edge: KIR2DL4 transduces signals into human NK cells through association with the Fc receptor gamma protein. J Immunol. (2005) 174:3859–63. doi: 10.4049/jimmunol.174.7.3859, PMID: 15778339

[53] Li D Wang Y Yang M Dong Z. mTORC1 and mTORC2 coordinate early NK cell development by differentially inducing E4BP4 and T-bet. Cell Death Differ. (2021) 28:1900–9. doi: 10.1038/s41418-020-00715-6, PMID: 33462410 PMC8184794

[54] Abel AM Yang C Thakar MS Malarkannan S. Natural killer cells: development, maturation, and clinical utilization. Front Immunol. (2018) 9:1869. doi: 10.3389/fimmu.2018.01869, PMID: 30150991 PMC6099181

[55] Zhou Y Chen B. GAS5−mediated regulation of cell signaling (Review). Mol Med Rep. (2020) 22:3049–56. doi: 10.3892/mmr.2020.11435, PMID: 32945519 PMC7453608

[56] Liu S Chu J Yucer N Leng M Wang SY Chen BP. RING finger and WD repeat domain 3 (RFWD3) associates with replication protein A (RPA) and facilitates RPA-mediated DNA damage response. J Biol Chem. (2011) 286:22314–22. doi: 10.1074/jbc.M111.222802, PMID: 21558276 PMC3121378

[57] Tsai CH Chen YJ Yu CJ Tzeng SR Wu IC Kuo WH. SMYD3-mediated H2A.Z.1 methylation promotes cell cycle and cancer proliferation. Cancer Res. (2016) 76:6043–53. doi: 10.1158/0008-5472.CAN-16-0500, PMID: 27569210

[58] Zhuge C Chang Y Li Y Chen Y Lei J. PDCD5-regulated cell fate decision after ultraviolet-irradiation-induced DNA damage. Biophys J. (2011) 101:2582–91. doi: 10.1016/j.bpj.2011.10.044, PMID: 22261045 PMC3297794

[59] Brazee PL Soni PN Tokhtaeva E Magnani N Yemelyanov A Perlman HR. FXYD5 is an essential mediator of the inflammatory response during lung injury. Front Immunol. (2017) 8:623. doi: 10.3389/fimmu.2017.00623, PMID: 28620381 PMC5451504

[60] Albini A Gallazzi M Palano MT Carlini V Ricotta R Bruno A. TIMP1 and TIMP2 downregulate TGFbeta induced decidual-like phenotype in natural killer cells. Cancers (Basel). (2021) 13(19):4955. doi: 10.3390/cancers13194955, PMID: 34638439 PMC8507839

[61] Kennedy M Awong G Sturgeon CM Ditadi A LaMotte-Mohs R Zuniga-Pflucker JC. T lymphocyte potential marks the emergence of definitive hematopoietic progenitors in human pluripotent stem cell differentiation cultures. Cell Rep. (2012) 2:1722–35. doi: 10.1016/j.celrep.2012.11.003, PMID: 23219550

[62] Dege C Fegan KH Creamer JP Berrien-Elliott MM Luff SA Kim D. Potently cytotoxic natural killer cells initially emerge from erythro-myeloid progenitors during mammalian development. Dev Cell. (2020) 53:229–39 e7. doi: 10.1016/j.devcel.2020.02.016, PMID: 32197069 PMC7185477

[63] Judge SJ Murphy WJ Canter RJ. Characterizing the dysfunctional NK cell: assessing the clinical relevance of exhaustion, anergy, and senescence. Front Cell Infect Microbiol. (2020) 10:49. doi: 10.3389/fcimb.2020.00049, PMID: 32117816 PMC7031155

[64] Edgar R Domrachev M Lash AE. Gene Expression Omnibus: NCBI gene expression and hybridization array data repository. Nucleic Acids Res. (2002) 30:207–10. doi: 10.1093/nar/30.1.207, PMID: 11752295 PMC99122

