# Supplementary material for: RAG recombinase expression discriminates the development of natural killer cells
Source: Front Immunol. 2025 Jul 25;16:1607664. doi: 10.3389/fimmu.2025.1607664 (PMC12331628; doi:10.3389/fimmu.2025.1607664)
Supplement: Supplementary file 1 [file DataSheet1.docx]

**RAG recombinase expression discriminates the development of natural killer cells**

**Running Title: RAG expression in natural killer cells**

**Table S1: Reagents and Resources**

| REAGENT or RESOURCE | SOURCE | IDENTIFIER |  |
| --- | --- | --- | --- |
| Antibodies |  |  |  |
| Alexa Fluor 488 Mouse IgG1 REA Control I Antibody | Miltenyi Biotec | 130-113-199 |  |
| Anti SSEA4 PerCP Vio700 | Miltenyi Biotec | 130-105-083 |  |
| Anti-Nanog-PE | Miltenyi Biotec | 130-105-080 |  |
| Anti-Oct3/4-APC | Miltenyi Biotec | 130-109-764 |  |
| APC anti-H2A.X-Phosphorylated (Ser139) Antibody | Biolegend | 613415 |  |
| APC Anti-Human CD140b Antibody | Miltenyi Biotec | 130-121-052 |  |
| APC Anti-Human CD158e1 (KIR3DL1, NKB1) Antibody | Biolegend | 312715 |  |
| APC Anti-Human CD161 Antibody | Biolegend | 339912 |  |
| APC Anti-Human CD181 (CXCR1) Antibody | Miltenyi Biotec | 130-105-394 |  |
| APC Anti-Human CD184 (CXCR4) Antibody | Miltenyi Biotec | 130-120-778 |  |
| APC Anti-Human CD3 Antibody | Biolegend | 34482 |  |
| APC Anti-Human CD314 (NKG2D) Antibody | Miltenyi Biotec | 130-111-999 |  |
| APC Anti-Human CD336 (NKp44) Antibody | Miltenyi Biotec | 130-120-623 |  |
| APC Anti-Human CD337 (NKp30) Antibody | Miltenyi Biotec | 130-112-503 |  |
| APC Anti-Human CD34 Antibody | Biolegend | 343509 |  |
| APC Anti-Human CD62L Antibody | Biolegend | 304809 |  |
| APC Anti-Human CD69 Antibody, Human, APC | Miltenyi Biotec | 130-114-046 |  |
| APC Anti-Human CD94 Antibody | Biolegend | 305508 |  |
| APC Anti-human IFN-γ Antibody | Biolegend | 502511 |  |
| APC Mouse IgG1 REA Control I Antibody | Miltenyi Biotec | 130-120-709 |  |
| APC mouse IgG1, K Isotype Control Antibody | Biolegend | 400120 |  |
| APC mouse IgG1, K Isotype Control Antibody | Biolegend | 400120 |  |
| APC-Cy7 Anti-Human CD15 (SSEA-1) Antibody | Biolegend | 323047 |  |
| APC-Cy7 Anti-Human CD45 Antibody | Biolegend | 368516 |  |
| APC-Cy7 Anti-Human CD56 Antibody | Biolegend | 318332 |  |
| APC-Cy7 Mouse IgG1, K Isotype Control Antibody | Biolegend | 400128 |  |
| FITC Anti- Human SOX2 FITC Antibody | Miltenyi Biotec | 130-120-790 |  |
| FITC Anti-Human CD144 FITC Antibody | Miltenyi Biotec | 130-126-010 |  |
| FITC Mouse IgG1 REA Control I Antibody | Miltenyi Biotec | 130-118-354 |  |
| PE Anti-Human CD107a (LAMP-1) Antibody | Biolegend | 328607 |  |
| PE Anti-Human CD158b (KIR2DL2/L3, NKAT2) Antibody | Biolegend | 312605 |  |
| PE Anti-Human CD159a (NKG2A) Antibody | Miltenyi Biotec | 130-114-092 |  |
| PE Anti-Human CD159c (NKG2C) Antibody | Miltenyi Biotec | 130-119-814 |  |
| PE Anti-Human CD226 (DNAM-1) Antibody | Miltenyi Biotec | 130-100-461 |  |
| PE Anti-Human CD335 (NKp46) Antibody | Biolegend | 331908 |  |
| PE Anti-Human CD43 Antibody | Biolegend | 343204 |  |
| PE Anti-Human CD57 Antibody | Biolegend | 322311 |  |
| PE Anti-Human CD7 Antibody | Biolegend | 343105 |  |
| PE Anti-Human Granzyme B Antibody, Human, PE | Miltenyi Biotec | 130-120-773 |  |
| PE Anti-Human NKp80 Antibody, Human, PE | Miltenyi Biotec | 130-112-779 |  |
| PE Anti-Human PAX6 Antibody | Miltenyi Biotec | 130-123-311 |  |
| PE Anti-Human Perforin Antibody | Miltenyi Biotec | 130-118-190 |  |
| PE Anti-Human SOX17 Antibody | Miltenyi Biotec | 130-111-148 |  |
| PE Mouse IgG1 REA Control I Antibody | Miltenyi Biotec | 130-118-347 |  |
| PE Mouse IgG1, K Isotype Control Antibody | Biolegend | 400112 |  |
| PE Mouse IgG1, K, MOPC-21 | Biolegend | 400112 |  |
| PE-Cy7 Anti-Human CD117 (C-Kit) Antibody | Biolegend | 313212 |  |
| PE-Cy7 Anti-Human CD16 Antibody | Biolegend | 560918 |  |
| PE-Cy7 mouse IgG1, K Isotype Control Antibody | Biolegend | 400125 |  |
| PerCP mouse IgG1, K Isotype Control Antibody | Biolegend | 400147 |  |
| PerCP-Cy5.5 Anti-Human CD45 Antibody | Biolegend | 368503 |  |
| PerCP-Cy5.5 Anti-Human CD56 Antibody | Biolegend | 318342 |  |
| Purified Anti-Mouse CD16 Antibody | Biolegend | 158002 |  |
| Purified Mouse IgG2, K Isotype Control Antibody | Biolegend | 402202 |  |
| Tra-1-60 Alexa Fluor 488 | Miltenyi Biotec | 330616 |  |
| Bacterial and virus strains | | |  |
| *E.coli* strain Top10™ | Provided by the TOPO TA™ Cloning Kits | K4575J10 |  |
| Stellar competent cells | Included in the In-Fusion™ HD Transformation Kit | 639689 |  |
| Chemicals, peptides, and recombinant proteins | | |  |
| 100 bp DNA Ladder | Thermo Fisher Scientific | 15628019 |  |
| 2-Mercaptoethanol (50 mM) | Gibco/ Thermo Fisher Scientific | 31350010 | |
| Accutase | Thermo Fisher Scientific | 00-4555-56 |  |
| Ampicillin Sodium Salt | Sigma Aldrich | A9518 |  |
| BamHI | NEB | R3136 |  |
| BD GolgiPlug™ | BD Biosciences | 555029 |  |
| BD Perm/Wash^TM^ | BD Biosciences | 554723 |  |
| Bovine Albumin Fraction V (7,5%) | Gibco/ Thermo Fisher Scientific | 15260037 | |
| Bromphenol blue | Merck Millipore | B0126 |  |
| BsmAI | NEB | R0529 |  |
| Cell Adhere™ Dilution Buffer | STEMCELL Technologies | 7183 | |
| Chloramphenicol | Sigma Aldrich | C0378 | |
| Colchicin | Eurobio | CCHCLC00-JA | |
| CutSmart Buffer | NEB | B6004 |  |
| Desoxyribonuclease I from Bovine Pancreas | Sigma Aldrich | DN24 |  |
| Dimethyl sulfoxide (DMSO) | Carl Roth | A994.1 | |
| Dimethylsulfoxid | Roth | 7029.1 |  |
| Dithiothreitol | Sigma Aldrich | 3819 |  |
| DMEM high glucose | Gibco/ Thermo Fisher Scientific | 11965092 | |
| dNTP Set, 100 mM Solutions | Thermo Fisher Scientific | R0181 |  |
| DPBS (no Ca^2+^ no Mg^2+^) | Gibco/ Thermo Fisher Scientific | 14190250 | |
| DPBS with Ca²^+^ and Mg²^+^ | Gibco/ Thermo Fisher Scientific | 14040133 | |
| Ethanolamide | Sigma Aldrich | 900131E |  |
| Ethidium bromide solution | Carl Roth | H341 |  |
| Fetal bovine serum (FBS) | PAN-Biotech | P30-3302 | |
| Fetal bovine serum (FBS) | Biowest | S1620 | |
| GlycoBlue^TM^ Coprecipitant (15 mg/mL) | Thermo Fisher Scientific | AM9516 |  |
| Ham´s F12 Nut Mix | Gibco/ Thermo Fisher Scientific | 21765-029 | |
| Heat Inactivated Human AB Serum | Valley Biomedical | HP1022 HI | |
| HindIII | NEB | R3104 |  |
| HotStar Taq DNA Polymerase | Qiagen | 203203 |  |
| Human bFGF | Thermo Fisher Scientific | PHG0369 |  |
| KnockOutTM Serum Replacement  (KOSR™) | Thermo Fisher Scientific | 10828-028 | |
| L-Ascorbic Acid | Sigma Aldrich | A-5960 |  |
| L-Glutamin 200 mM | Gibco/ Thermo Fisher Scientific | 25030081 |  |
| LB Agar | Sigma-Aldrich | L3022 |  |
| LB Broth | Roth | X969.1 |  |
| MEM Non-Essential Amino Acid Solution 100x | Gibco/ Thermo Fisher Scientific | 11140-035 |  |
| MEM-alpha | Gibco/ Thermo Fisher Scientific | 22561-021 |  |
| Mitomycin C | STEMCELL Technologies | 73272 |  |
| MluI | NEB | R3198 |  |
| Nonident^TM^ P40-Ersatz | Thermo Fisher Scientific | J19628.K2 |  |
| NotI | NEB | R3189 |  |
| Penicillin Streptomycin 1000 U | Gibco/ Thermo Fisher Scientific | 15140122 |  |
| peqGOLD 1 kb DNA-Ladder | VWR | 25-2030 |  |
| Phusion High Fidelity Polymerase | Thermo Fisher Scientific | F530S |  |
| Proteinase K | Sigma Aldrich | 107393001 |  |
| Puromycin | STEMCELL Technologies | 73342 |  |
| Random Hexamer Primers | Thermo Fisher Scientific | N8080127 |  |
| Recombinant Human Flt3 Ligand | Prepotech | 300-19 |  |
| Recombinant Human IL-15 | Prepotech | 200-15 |  |
| Recombinant human IL-2 | R&D Systems | 202-IL |  |
| Recombinant Human IL-3 | Prepotech | 200-03 |  |
| Recombinant Human IL-7 | Prepotech | 200-07 |  |
| Recombinant Human SCF | Prepotech | 300-07 |  |
| ReLeSR™ | STEMCELL Technologies | 5872 |  |
| RiboRuler Low Range RNA Ladder | Thermo Fischer Scientific | SM1831 |  |
| RNase solution | Promega | A7973 |  |
| RNAseP™ Detection Reagent | Thermo Fisher Scientific | 4316831 |  |
| RPMI 1640 Medium | Gibco/ Thermo Fisher Scientific | 11875093 |  |
| S.O.C. medium | Invitrogen / Thermo Fisher Scientific | 15544034 |  |
| SalI | NEB | R3138 |  |
| Sodium Selenite | Sigma Aldrich | S5261 |  |
| StemMACS™ iPSC brew XF, human | Miltenyi | 130-104-368 |  |
| TRI Reagent | Sigma Aldrich | 93289 |  |
| UltraPure™ 0.5M EDTA, pH 8.0 | Thermo Fisher Scientific | 15575020 | |
| Vitronectin™ XF | STEMCELL Technologies | 7180 |  |
| XhoI | NEB | R0146 |  |
| Y-27632 | STEMCELL Technologies | 72304 |  |
| ZymoTaq | Zymo Research | E2003 |  |
| Commercial Assays | |  |  |
| Amaxa® Cell Line Nucleofector® Kit V | Lonza | VCA-1003 |  |
| Amaxa® Human Stem Cell Nucleofector® Kit 2 | Lonza | VPH-5022 |  |
| BD Cytofix/Cytoperm^TM^ | BD Biosciences | 554714 |  |
| BioSprint 15 DNA Blood Kit | Qiagen | 940014 |  |
| Cell Signalling Buffer Set A | Miltenyi Biotec | 130-100-827 |  |
| Cell Trace Far Red Cell Proliferation Kit | Invitrogen | C34564 |  |
| DNA Polymerization Mix dNTP Set | Thermo Fisher Scientific | AB0196 |  |
| ExoSAP-IT™ PCR Product Clean-up | Applied Biosystems/fisher scientific | 15869896 |  |
| Fix and Perm Cell Permeabilization Kit | Invitrogen/Thermo Fisher Scientific | GAS003 |  |
| FOXP3 Staining Buffer Set | Miltenyi Biotec | 130-093-142 |  |
| Fragment Analyzer DNA HS NGS fragment kit | Agilent Technologies | DNF-474-0500 |  |
| Fusion™ HD TransformationKit Eco Dry | Takara Bio | 639689 |  |
| High Speed Plasmid Maxi Kit (25) | Qiagen | 126662 |  |
| KAPA Mouse Genotyping Kit | Biosystems/ Sigma-Aldrich | KK730 |  |
| MEGAClear Kit | Thermo Fisher Scientific | AM1908 |  |
| MEGAshortscript Kit | Thermo Fisher Scientific | AM1354 |  |
| mMACHINE™ T7 Transcription Kit | Thermo Fisher Scientific | AM1344 |  |
| NEBNext Single Cell/Low Input RNA Library Prep Kit | NEB | E6420 |  |
| NEBnext® single cell lysis module | NEB | E5530 |  |
| Poly(A) Tailing Kit | Thermo Fisher Scientific | AM1350 |  |
| QIAGEN Plasmid Mini Kit (100) | Qiagen | 12125 |  |
| QIAquick Gel Extraction Kit (50) | Qiagen | 28704 |  |
| QIAquick PCR Purification Kit (50) | Qiagen | 28104 |  |
| Qubit Fluorometric Quantitation and dsDNA High sensitivity assay | Thermo Fisher Scientific | Q32851 |  |
| Rotor-Gene SYBR® Green PCR Kit | Qiagen | 204001 |  |
| STEMdiff™ Hematopoitic Kit | STEMCELL Technologies | 05310 |  |
| StemMACS™ Trilineage Differentiation Kit | Miltenyi | 130-115-660 |  |
| SuperScript™IV Reverse Transcriptase | Thermo Fisher Scientific | 18090200 |  |
| TOPO TA™ Cloning Kits | Thermo Fisher Scientific | K4575J10 |  |
| WizardR™ Genomic DNA Purification Kit | Promega | A1120 |  |
| Cell lines | |  |  |
| Human BJ1 Fibroblasts | Kindly gifted by Prof. Dr. Luigi Notarangelo, NIH, Washington, USA | |  |
| Human iPSC | Generated from human foreskin fibroblasts NUFF-1 (Global Stem Cells, GSC-3002, Lot: 61500063) in the laboratory of Prof. Dr. Toni Cathomen, University Medical Center Freiburg, Freiburg, Germany (1) | |  |
| RSS-EGFP+/+ iPSC | This study | |  |
| RSS-EGFP+/- iPSC | This study | |  |
| Human lymphoblast K562 | DMSZ – German Collection of Microorganisms and Cell Cultures (ACC Nr. 10) | |  |
| Human NHDF Fibroblasts | Kindly provided by Dr. Klaus Schwarz, Ulm, Germany | |  |
| Murine mastocytoma P815 | Kindly gifted by Prof. Dr. Stefan Ehl, University Medical Center Freiburg, Freiburg, Germany (2) | |  |
| Murine stroma cells OP9-DL1 | Kindly gifted from Prof. Dr. Jean Plum laboratory, Ghent, Belgium | |  |
| Primers and oligonucleotides | | |  |
| Cloning pmx RSS cGFP_F | TATCGAATTCACGCGTGTAGGCTGCACAGTGGTA | |  |
| Cloning pmx RSS cGFP_R | TTGGCAAAGAATTCGAGTCGAGGTTTTTGTTCCA | |  |
| RSS cGFP AAVS1-insert_F | CATTTTGGCAAAGAATTCG | |  |
| RSS cGFP AAVS1-insert_R | AGCTTGATATCGAATTCACG | |  |
| sgRNA AAVS1 | GAAATTAATACGACTCACTATAGGGGCCACTAGGGACAGGATGTTTTAGAGCTAGAAATAGCA | |  |
| sgRNA scaffold template *S.pyogenes* | AAAAGCACCGACTCGGTGCCACTTTTTCAAGTTGATAACGGACTAGCCTTATTTTAACTTGCTATTTCTAGCTCTAAAAC | |  |
| Chimeric_Intron_WPRE_F | CTTCTCCCTCTCCAGCCTC | |  |
| Chimeric_Intron_WPRE_R | CGACAACACCACGGAATTGTCAGT | |  |
| Chimeric_Intron_F | CTCTGACTGACCGCGTTACTCC | |  |
| Chimeric_Intron_R | ATGAGACAGCACAATAACCAGCACG | |  |
| CMV_F | CGACTGTGCCTTCTAGTTGCCA | |  |
| CMV_R | CCGCTCACCTGTGGGAGTA | |  |
| Puro_F | CCTCGAGAGATCTGGCAGCG | |  |
| Puro_R | AGGCTGATCAGCGGGTTTAAACG | |  |
| Puro_HA-L_R | GTGGGCTTGTACTCGGTCATC | |  |
| PPP1R12C_HA-L_F | GCTTAGCCACTCTGTGCTGAC | |  |
| PPP1R12C_HA-R_R | CGTAACCTGGAGGGAATCCC | |  |
| WPRE_HA-R_F | CGTTGTCAGGCAACGTGGC | |  |
| DNMT3B_F | ATAAGTCGAAGGTGCGTCGT | |  |
| DNMT3B_R | GGCAACATCTGAAGCCATTT | |  |
| GDR2_F | AAATGTTTGTGTTGCGGTCA | |  |
| GDR2_R | TCTGGCACAGGTGTCTTCAG | |  |
| hTERT_F | TGTGCACCAACATCTACAAG | |  |
| hTERT_R | GCGTTCTTGGCTTTCAGGAT | |  |
| NANOG_F | TGAACCTCAGCTACAAACAG | |  |
| NANOG_R | TGGTGGTAGGAAGAGTAAAG | |  |
| OCT4_F | CCTCACTTCACTGCACTGTA | |  |
| OCT4_R | CAGGTTTTCTTTCCCTAGCT | |  |
| SOX2_F | AGCTACAGCATGATGCAGGA | |  |
| SOX2_R | GGTCATGGAGTTGTACTGCA | |  |
| KLF4_F | TCTCAAGGCACACCTGCGAA | |  |
| KLF4_R | TAGTGCCTGGTCAGTTCATC | |  |
| MYCC_F | ACTCTGAGGAGGAACAAGAA | |  |
| MYCC_R | TGGAGACGTGGCACCTCTT | |  |
| ACTB_F | CCAACACAGTGCTGTCTG | |  |
| ACTB_R | CAACTAAGTCATAGTCCGCC | |  |
| POL2_F | ACGCTGCTCTTCAACATCCA | |  |
| POL2_R | GGCAGACACACCAGCATAGT | |  |
| RPL13A_F | CGGACCGTGCGAGG | |  |
| RPL13A_R | CACCATCCGCTTTT | |  |
| UBC_F | CTAGTTCCGTCGCAGCCGGGA | |  |
| UBC_R | TGGTGTCACTGGGCTCAACCTCG | |  |
| Recombinant DNA | | |  |
| AAVS1-Pur-CAG-EGFP | EGFP #80945 was purchased from Addgene, originally gifted from Su-Chun Zhang (3) | |  |
| pMX-RSS-GFP/IRES-hCD4  (pMX-INV) | Kindly gifted by Prof. Dr. Luigi Notarangelo, NIH, Washington, USA (4) | |  |
| AAVS1-Pur-CAG RSS-EGFP | This study | |  |
| pcDNA6-WTRAG1 | Kindly provided by Dr. Klaus Schwarz, Ulm, Germany (5, 6) | |  |
| pcDNA6-WTRAG2 | Kindly provided by Dr. Klaus Schwarz, Ulm, Germany (5, 6) | |  |
| Software and algorithms | | |  |
| BD FACSDiva^TM^ | BD Biosciences | https://www.bdbiosciences.com/en-us/products/software/instrument-software/bd-facsdiva-software |  |
| CLC Genomics Workbench | Qiagen | https://digitalinsights.qiagen.com/ |  |
| EndNote^TM^ V20 | Clarivate | https://endnote.com/de/ |  |
| ENSEMBL BioMart | ENSEMBL | https://www.ensembl.org/info/data/biomart/index.html |  |
| FastQC v0.11.8 (7) | https://www.bioinformatics.babraham.ac.uk/projects/fastqc/ | |  |
| featureCounts v1.6.4 (8). | https://subread.sourceforge.net/featureCounts.html | |  |
| FlowJo V10 | BD Bioscience | https://www.flowjo.com/ |  |
| GALAXY v24.2.2 (9) | https://usegalaxy.org/ | |  |
| GO Ontology database (2024-06-17), PANTHER^TM^ v19, Reactome^TM^ v86 (10, 11, 12) | Gene Ontology Resource | https://geneontology.org/ |  |
| ImmunoSEQ Analyzer | Adaptive Biotechnologies | https://clients.adaptivebiotech.com/ |  |
| MATLAB | MathWorks | https://www.mathworks.com/products/matlab.html |  |
| Microsoft office 2019 | Microsoft | https://www.microsoft.com |  |
| Morpheus | Broad Institute, Cambridge, MA, USA | https://software.broadinstitute.org/morpheus |  |
| MultiQC v1.74 (13) | Seqera | https://seqera.io/multiqc/ |  |
| nf-core/bamtofastq v 2.0.0 | nf-core | https://nf-co.re/bamtofastq/2.0.0/ |  |
| **nf-core/rnaseq** (14) | nf-core | https://nf-co.re/rnaseq/1.4.2 |  |
| Prism V9 and V10 | GraphPad | https://www.graphpad.com/ |  |
| Python 3.12 | The Python Software Foundation | https://www.python.org/ |  |
| R packages: DESeq2 v1.40.2 (15), ggplot2 v3.4.2 (R v4.3.1), rmarkdown v2.23, knitr v1.43, DT v0.28 | R | https://www.r-project.org/ |  |
| RSeQC v3.0.1 (16) | https://rseqc.sourceforge.net/ | |  |
| SRplot | SRplot: A free online platform for data visualization and graphing (17) | http://www.bioinformatics.com.cn/en |  |
| STAR v2.6.1d (18) | https://github.com/alexdobin/STAR | |  |
| Trim Galore v0.6.43 (19) | https://www.bioinformatics.babraham.ac.uk/projects/trim_galore/ | |  |
| Other | | |  |
| Cell strainers 40µm | Corning® | CLS431750 |  |
| QIAshredder spin columns | Qiagen | 79656 |  |
| StemPro EZPassage Disposable Passaging Tool | Thermo Fisher Scientific | 23181010 |  |
| Ultra-low attachment plates (6-well) | Corning® Costar® | CLS3471 |  |

**Supplementary Methods**

**CRISPR/Cas9 gene-editing**

The sequence of the sgRNA was published before (3, 20). The sgRNA was synthesized *in vitro* using the MEGAshortscript^TM^ Kit (Ambion). A ribonucleoprotein (RNP) complex was formed using 250 pmol Streptococcus pyogenes wild-type Cas9 protein (NEB) and 500 pmol of the synthesized sgRNA at a molar ratio of 1:2 at 37°C for 15min. IPSC were pretreated with StemMACS™ iPSC brew XF (Milteniy) supplemented with 10µM ROCK inhibitor (STEMCELL Technologies) for 1h prior nucleofection. IPSC were harvested using Accutase™ (Thermo Fisher Scientific) to prevent cell clumping and to ensure single cell suspension. 1x10^6^ iPSCs were targeted with 750pmol ribonucleoprotein (RNP) complex and 30µg AAVS1-PUR-CAG-RSS-cEGFP plasmid DNA by nucleofection using the Human Stem Cell Nucleofector™ Kit 2 (Amaxa, Lonza) with the program B06. After 2 days, medium was supplemented with 1µg/ml puromycin (Thermo Fisher Scientific). Single cell clones were selected and characterized for target integration, pluripotency and karyotypic integrity.

**Copy number variation (CNV) calculation**

The copy number of reporter constructs integrated into the iPSC genome was determined by quantitate PCR (**Figure S1B**). Genomic DNA was isolated from selected iPSC clones that were successfully transfected with the reporter construct and *EGFP* was amplified using 20ng DNA. To calculate the construct copies within one sample standard curves for the target gene *EGFP* and the reference gene *RNAseP*™ were generated. To establish the standard curve for *EGFP*, the plasmid AAVS1-PUR-CAG-RSS-EGFP was digested using the restriction enzyme BsmAI and NotI followed by PCR purification. Copies per reaction were calculated for the serial dilution of 10^8^ to 10^1^ and mixed with 20µg of genomic DNA from healthy control iPSC to serve as background. For the standard curve of *RNAseP™*, serial dilutions from 5ng to 120ng gDNA were generated. All reactions were performed in triplicates. The relative transgene copy number per genome was calculated using the 2^-Δct^ method (21, 22). The calculation of the concentration assumes that 1 cell contains 3,3pg of haploid DNA.

**Confirmation of pluripotency and karyotypic integrity**

Karyotyping was performed on healthy control iPSC, and generated reporter iPSC lines RSS-EGFP^+/+^ and RSS-EGFP^+/-^, respectively, in the Department of Human Genetics of Ulm University, Ulm, Germany. No chromosomal abnormalities were observed in any iPSC line.

Expression of pluripotency related genes (*DNMT3B, GDF, hTERT, NANOG, OCT4, SOX*) and reprogramming-associated genes (*MYCC, KLF4*) was assessed by quantitative PCR using the Rotor-Gene SYBR^®^ Green PCR Kit (Qiagen) on a Rotor Gene Q device (Qiagen) (**Figure S1C**). *HPRT1*, *RPL13A*, and *UBC* were used as reference genes and data were normalized to NHDF fibroblasts as described before (1).

In addition, expression of *SSEA-4, TRA-1-60, OCT43/4*, and *NANOG* was assessed by flow cytometry using fluorochrome-labeled antibodies and respective isotypes (Miltenyi Biotec) listed in the KRT. Cells were fixed and permeabilized using the FOXP3 staining buffer set (Miltenyi Biotec) (**Figure S1D**).

Differentiation potential into the three germ layers was investigated using the human StemMACS™ Trilineage Differentiation Kit (Miltenyi Biotec) and verified by flow cytometry (mesoderm: CD140b^+^, CD144^+^, endoderm: SOX17^+^/CD184^+^, ectoderm: SOX2^+^/PAX6^+^) (**Figure S1E**).

**V(D)J recombination capacity of RSS-EGFP reporter iPSC**

V(D)J mediated transposition of unidirectional RSS12 and RSS23 flanking the complement *EGFP* gene was investigated in iPSC reporter lines RSScEGFP^+/+^, and RSScEGFP^+/-^, and iPSC-derived HSPC, respectively. 10µg of target vector AAVS1-PUR-CAG-RSS-cEGFP and plasmids encoding for RAG1 (pcDNA6-WTRAG1) and RAG2 (pcDNA6-WTRAG1) under the control of the CMV promotor (5, 6) were transiently transfected into 1x10^6^ cells at a molar ratio of 1:1 using the Amaxa® human Stem Cell Nucleofection Kit 2 (Lonza, program B06). After 48h cells were harvested using Accutase™ and analyzed for GFP expression by flow cytometry (**Figure S1F**).

**Transfection of RAG1 and RAG2 mRNA into HSPC**

HSPC harvested at d12 of the hematopoietic differentiation were transfected with 2µg RAG1 and 4µg RAG2 mRNA per 1x10^6^ cells, respectively, using the Amaxa® Human Stem Cell Nucleofector Kit 2 (Lonza, program B16). Transfected HSPC were further differentiated into NK cells and analyzed for NK lineage marker expression at week 1, week 2, and week 3 (**Figure S7 and S8**).

**RNA Sequencing**

For each sample and time point, 500 GFP^+^ and GFP^-^ (RSS-EGFP^+/+^) NK progenitor cells were sorted on day 19 (week 1), 26 (week 2) and 33 (week 3) into 25µl of NEBnext® cell lysis buffer (New England Biolabs) and stored at -80°C. Samples were used for cDNA synthesis and library preparation using the NEBNext Single Cell/Low Input RNA Library Prep Kit (New England Biolabs) following the protocol for Cell instruction and 17 PCR cycles. The library molarity was determined by measuring the library size (approximately 350 bp) using the Fragment Analyzer 5300 and the Fragment Analyzer DNA HS NGS fragment kit (Agilent Technologies) and the library concentration (>0.75 ng/µl) using Qubit Fluorometric Quantitation and dsDNA High sensitivity assay (Thermo Fisher Scientific). The libraries were denaturated according to the manufacturer’s instructions, diluted to 126 pM and sequenced as paired-end 100bp reads on an Illumina NovaSeq 6000 (Illumina). The sequencing aimed to achieve a depth of approximately >20 million clusters per sample. Read quality of RNA-seq data in raw fastq files was assessed using nf-core/rnaseq v 1.4.2 (https://nf-co.re/rnaseq/1.4.2) to identify sequencing cycles with low average quality, contaminations and/or repetitive/duplicate sequences from PCR amplification.

**Bioinformatic analysis of RNA-Seq data**

The Nextflow-based nf-core pipeline rnaseq v1.4.2 was used for the RNA-Seq Bioinformatics analysis. As part of this workflow, FastQC (version v0.11.8) (7) was used to determine the quality of the FASTQ files. Subsequently, adapter trimming was conducted with Trim Galore v0.6.43 (19). STAR v2.6.1d (18) aligner was used to map the reads that passed the quality control to the human reference genome (GRCh37). The RNA-Seq data quality control was performed with RSeQC v3.0.1 (16) and read counting of the features (e.g. genes) with featureCounts v1.6.4 (8). An aggregation of the quality control for the RNA-Seq analysis was performed with MultiQC v1.74 (13).

In this report, many QC values pointed to significant DNA contamination, which also varied between sample groups (higher in GFP- compared to GFP+ samples). These values included the non-strandedness of reads, high portion of intronic and intergenic compared to exonic reads and low number of spliced reads/ junction sites), as previously reported (23). To avoid systematic bias in downstream processing of the data, DNA contamination was reduced using a customized Snakemake workflow integrating a filtering step to retain reads based on gapped alignments only (N in CIGAR string of SAM/BAM files), beyond standard QC filtering (e.g. removal of duplicates). Further filtering was applied to maintain only the gapped mapping reads that matched with an exon at the start and at the end of the read, excluding potential DNA derived reads covering exon-intron, intron-exon, and intron-intron regions. Those filtered reads were extracted from the BAM files, re-converted to fastq files using nf-core/bamtofastq v 2.0.0 and re-run then with nf-core/rnaseq v1.4.2 to obtain a more DNA cleaned RNA-Seq count table for downstream DE analysis, which represented ~1.4 % of the data (~40k reads per samples, **Figure S11A**). Filtered data showed an improvement of read mapping events towards exonic regions while mapping to intergenic and intronic regions was slightly reduced (**Figure S11B**), as previously reported (23). There was no improvement detected on strandedness of the reads, which can be attributed to the NEBNext Single Cell /Low Input RNA Library Prep Kit used, which makes non-directional libraries (**Figure S11C**). This filtered data was subsequently analyzed for differential expression between GFP^-^ and GFP^+^ NK cells at week 1, 2 and 3.

Differential gene expression analysis was then performed using the rnadeseq pipeline (<https://github.com/qbic-pipelines/rnadeseq> version 2.2). For differential expression analysis, the DNA cleaned RNA-Seq count table resulting from featureCounts was processed with the R package DESeq2 v1.40.2 (15). Graphs were produced in RStudio with R version 4.3.1 (2018-07-02) mainly using the R package ggplot2 v3.4.2. Final reports were produced using the R package rmarkdown v2.23 with the knitr v1.43 and DT v0.28 R packages. The sample similarity heatmap was created using the edgeR v3.26.5 R package.

GO enrichment analysis was performed for DEG shared by all time points (week1, week2, week3), as well as for each time point separately using the Gene Ontology Resource (<https://geneontology.org/>) (GO Ontology database DOI: 10.5281/zenodo.12173881 Released 2024-06-17), PANTHER^TM^ (version 19, released 2024-06-20), and Reactome (version 86, released 2023-09-07) (10, 11, 12). Results are presented in **Table S2**. GO terms with a false discovery rate (FDR) < 5% (FDR<0.05) were categorized into the groups 1) Gene Expression (GE) and Translation, 2) Metabolism, and 3) Cellular Function avoiding overlapping GO terms. Results are shown in **Figure 5G** and **S12D-F**. Differentially regulated pathways identified by Reactome, were categorized into three functional groups 1) Hematopoietic Differentiation, 2) DNA Damage Response, and 3) Immune Function. Fold induction, p-value and gene counts are visualized for each time point (w1, w2, w3) in **Figure 6A-C**.

**Supplementary figure legends**

**Figure S1: Generation of RAG-fate mapped reporter iPSC lines and characterization of pluripotency and recombination potential.**

A) Integration of the reporter construct into the AAVS1 locus was confirmed by PCR. Genomic DNA was isolated from targeted iPSC clones selected with puromycin. Integration sites with and without construct integration were amplified as indicated in the top graphic. Images of representative agarose gels are shown at the bottom. Wild type iPSC (wt), and wild type iPSC transfected with the donor plasmid (wt*) were used as controls. Size markers are indicated on the left. Cell lines used in this study are highlighted by red squares (#6 corresponds to EGFP-RSS^+/+^, #26 corresponds to EGFP-RSS^+/-^).

B) The number of transgene copies expressed in targeted iPSC lines was assessed by quantitative PCR and calculated based on the expression of the EGFP cassette of the reporter construct and *RNAseP*™ using the 2^-∆CT^ method. Shown is the relative copy number expression per genome obtained from 4 iPSC lines (#6 corresponds to EGFP-RSS^+/+^, #26 corresponds to EGFP-RSS^+/-^) in 3 replicate measurements. An integration of the reporter cassette on both alleles results in a copy number of 2, whereas a monoallelic integration leads to a copy number of 1.

C) Pluripotency was investigated in untargeted (wt), and targeted iPSCs with bi- (RSS-EGFP^+/-^) and monoallelic (RSS-EGFP^+/+^) integrations of the reporter cassette, respectively, using RT-PCR. Shown are relative expression levels of pluripotency associated genes *DNMT3B, GDR3, HTERT, NANOG, OCT4*, and *SOX2* normalized on BJ1 fibroblasts. Expression of the genes *CMYC* and *KLF4* was investigated to confirm termination of the reprogramming process. Results were normalized on reference genes *RPI13A, UBC* and *POL2*.

D) Expression of pluripotency associated proteins SSEA4, TRA1-60, OCT3/4, and NANOG was investigated in untargeted (WT), and targeted iPSC lines with bi- (RSS-EGFP^+/-^) and monoallelic (RSS-EGFP^+/+^) integrations of the reporter cassette, respectively, using flow cytometry. Expression levels of indicated markers are shown (red) compared to isotypes (black).

E) Differentiation potential into mesodermal, ectodermal and endodermal germ layers was investigated in wild type and reporter cell lines (wt, RSS-EGFP^+/+^, and RSS-EGFP^+/-^). IPSC treated with differentiation media (black), or non-treated (grey), respectively, were investigated for expression of representative markers using flow cytometry. Mesodermal cells were identified by expression of CD144 (VE-Cadherin) and CD140b, cells differentiated into the endodermal lineage were characterized as SOX17^+^ and CD184^+^ (CXCR4), and ectodermal cells as SOX2^+^ and PAX6^+^.

F) The performance of the RAG-fate mapping reporter cassette was investigated *in vitro* in HSPC by additional transfection of RAG1 and RAG2 mRNA to induce V(D)J recombination. The recombination efficiency was calculated by GFP expression resulting from flipping of the targeted reporter cassette in HSPCs obtained from wild type (wt), RSS-EGFP^+/+^, and RSS-EGFP^+/-^ iPSC lines. Shown are mean percentages of GFP^+^ cells observed in non-transfected HSPCs (Ø), and after RAG1/RAG2 mRNA transfection, respectively, obtained from 3 independent experiments. Statistical analysis was performed using 2Way ANOVA with Bonferroni posttest (*p<0.05, **p<0.01, ***p<0.001, ****p<0.0001).

G) Graphical timeline of the differentiation process. HSPC and NK cell differentiation was monitored for NK lineage marker expression at indicated time points using flow cytometry. The phenotype observed in GFP^-^ and GFP^+^ iPSC-derived NK cell progenitors is shown, and major differences are highlighted.

**Figure S2: RAG-fate mapped NK cells are characterized by CD45^dim^ expression.**

A) Distribution of CD45^bright^ and CD45^dim^ expression in GFP^+^ and GFP^-^ NK cell populations, respectively, is shown for NK cells obtained from RSS-EGFP^+/-^ iPSC (CD45^bright^ + CD45^dim^ = 100%).

B) Distribution of CD45^bright^CD56^bright^/CD56^dim^, and CD45^dim^CD56^bright^/CD56^dim^ populations is shown for GFP^-^ and GFP^+^ NK cells (RSS-EGFP^+/-^), respectively.

C) Expression of CD45RA, CD45RO, CD45RB, and CD45RC isotypes was analyzed in GFP^+^ and GFP^-^ cells using flow cytometry. The representative gating strategy is shown on mature NK cells obtained at week 3 of differentiation.

In addition, CD45 isotypes were investigated in peripheral blood NK cells obtained from healthy buffy coat donors (n=3). Shown are the mean percentage distributions of CD45RA, RB, RC, and RO isotypes within CD45^bright^ and CD45^dim^ populations in a bar diagram (B) and in donut charts (C).

**Figure S3: The phenotype of GFP^-^CD45^bright^ and GFP^+^CD45^dim^ NK cells results from differential CD45 isotype expression.**

The CD45 isotype distribution was evaluated in hematopoietic stem progenitor cells (HSPC), and NK progenitor cells obtained from RSS-EGFP^+/+^(A), and RSS-EGFP^+/-^ (B) iPSCs, respectively, using flow cytometry. The percentage distribution of CD45 isotypes in CD45^bright^ and CD45^dim^ HSPCs, and NK progenitors obtained at weeks 1, 2, and 3 are shown in donut charts.

Expression of CD45 isotypes was evaluated in HSPCs and NK cell precursors derived from RSS-EGFP^+/+^ (C), and RSS-EGFP^+/-^ (D) iPSC lines, respectively, at indicated time points. The mean percentages +/- SEM (n=3) of each isotype combination are shown in GFP^+^ and GFP^-^ NK cell populations.

Statistical analysis was performed using 2Way ANOVA with Bonferroni posttest (*p<0.05, **p<0.01, ***p<0.001, ****p<0.0001).

**Figure S4: GFP^-^CD45^bright^ and GFP^+^CD45^dim^ HSPCs differ in hematopoietic potential and maturity.**

A) The hematopoietic differentiation potential was characterized in HSPCs by expression of CD34, CD43, and CD15 using flow cytometry. The representative gating strategy for these surface markers within CD45^bright^ and CD45^dim^ HSPC populations is shown. GFP expression could not be detected at this stage of differentiation.

The percentage of CD34^+^CD43^-^, CD34^+^CD43^-^, CD34^-^CD43^+^, and CD15^+^ expressing populations is shown for CD45^bright^ and CD45^dim^ HSPCs obtained from RSS-EGFP^+/+^ (B), and RSS-EGFP^+/-^ (C) iPSC lines, respectively. Shown are means +/- SEM from at least 3 experiments (n=3). Statistical analysis was performed using 2Way ANOVA with Bonferroni posttest (*p<0.05, **p<0.01, ***p<0.001, ****p<0.0001).

**Figure S5: NK lineage surface marker expression shows differential maturation of RAG-fate mapped and non-mapped NK cells at week 1, 2, and 3.**

IPSC were differentiated into NK cells and studied weekly for NK lineage marker expression using flow cytometry.

A) Gating strategy for the evaluation of CD56, CD117, CD161, CD94, CD335 (NKp46), CD7, and CD16 expression on GFP^-^ and GFP^+^, CD45^+^ (isotype shown in blue) progenitor cells obtained at week 1, week 2, and week 3. Gates were adjusted according to isotype and unstained controls.

B) Expression of indicated surface markers were analyzed in GFP^+^ versus GFP^-^ CD45^+^ NK cell precursors (RSS-EGFP^+/-^ PSCs).

Expression of indicated surface markers were analyzed in GFP^-^ versus GFP^+^ CD45^bright^ and CD45^dim^ NK cell precursors derived from RSS-EGFP^+/+^ (C), and RSS-EGFP^+/-^ iPSC (D), respectively.

**Figure S6: NK lineage surface marker expression indicates a more mature phenotype in RAG-fate mapped NK cells.**

A) Expression of indicated NK cell marker were studied in GFP^+^ versus GFP^-^ CD56^+^ NK cell precursors (RSS-EGFP^+/-^).

At week 3 of the differentiation protocol, indicated surface marker were analyzed on CD56^+^ GFP^-^ versus GFP^+^ NK cells. Shown are results for CD56^bright^ (top) and CD56^dim^ (bottom) RSS-EGFP^+/+^ (B), and CD56^bright^ (top) and CD56^dim^ (bottom) RSS-EGFP^+/-^ iPSC-derived NK cells (C), respectively.

Shown are means +/- SEM from 4-6 experiments. Statistical analysis was performed using 2Way ANOVA with Bonferroni posttest (*p<0.05, **p<0.01, ***p<0.001, ****p<0.0001).

**Figure S7: Additional transfection of RAG1 and RAG2 mRNA contrasts phenotypical differences between GFP^+^ and GFP^-^ NK cells.**

Additional RAG1/2 mRNA was transduced into HSPCs by nucleofection to induce targeting of the reporter construct. A) Distribution of CD45^bright^ and CD45^dim^ expression in GFP^-^ and GFP^+^ NK cell populations, respectively, is shown for both reporter cell lines. Distribution of GFP^-/+^CD56^bright^ and GFP^-/+^CD56^dim^ expression is shown for CD45^bright^ (B) and CD45^dim^ (C) NK cell populations, respectively, is derived from both reporter cell lines as indicated.

Transfected HSPCs were subsequentially differentiated into NK cells and surface expression of CD7, CD117, CD161, NKp46, CD94 and CD16 was analyzed in GFP^-^ versus GFP^+^ CD45^bright^, CD45^dim^, and total CD45^+^ NK cell precursors obtained from RSS-EGFP^+/+^ (D), and RSS-EGFP^+/-^ iPSC (E), respectively.

F) The heatmap visualizes the mean expression of these markers in CD45^bright^, CD45^dim^, and total CD45^+^ NK cells derived from both reporter cell lines (RSS-EGFP^+/+^, RSS-EGFP^+/-^) as indicated.

Shown are means +/- SEM obtained from at least 3 experiments. Statistical analysis was performed using 2Way ANOVA.

**Figure S8: Induced RAG1/RAG2 expression leads to terminal differentiation in RAG-fate mapped NK cells.**

Expression of indicated surface marker were studied in GFP^+^ versus GFP^-^ CD56^bright^ (A, B), CD56^dim^ (C, D), and total CD56^+^ NK cell precursors (E, F) obtained from RAG1/RAG2-transfected RSS-EGFP^+/+^ and RSS-EGFP^+/-^ HSPCs, respectively.

G) Heatmap summarizing the mean expression of indicated surface markers detected in GFP^-^ and GFP^+^ CD56^bright^, CD56^dim^, and CD56^+^ NK cells after 3 weeks of differentiation analyzed by flow cytometry. Results obtained from both reporter cell lines (RSS-EGFP^+/+^, RSS-EGFP^+/-^) are shown as indicated.

Shown are results as means +/- SEM from 3 experiments (n=3). Statistical analysis was performed using 2Way ANOVA with Bonferroni posttest (*p<0.05, **p<0.01, ***p<0.001, ****p<0.0001).

**Figure S9: DNA damage response and survival is diminished in RAG-fate mapped NK cells.**

A) NK cells obtained at week 1, 2 and 3 of differentiation were irradiated with 2Gy and fixed at indicated time points. Geometric mean fluorescent intensities (MFI) of γH2AX are shown for GFP^+^ and GFP^-^ NK cell populations at indicated time points after irradiation.

B) The survival responses were analyzed in GFP^-^ and GFP^+^ populations at indicated time points after irradiation. Percentage of vitality was assessed by normalization on unirradiated cells. Shown are results obtained from RSS-EGFP^+/-^ iPSC as means +/- SEM from at least 3 experiments (n=3). Statistical analysis was performed using 2Way ANOVA with Bonferroni posttest (*p<0.05, **p<0.01, ***p<0.001, ****p<0.0001).

**Figure S10: RAG-fate mapped NK cells have unproductive rearrangements on the IGH locus.**

A) GFP^+^ and GFP^-^ NK cells were sorted at week 3 and gDNA was isolated. Genomic rearrangements in heavy chains, and kappa and lambda light chains of the immunoglobulin receptor, as well as rearrangements in the TCR beta, gamma and delta loci were investigated by end-point PCR. Additionally, aberrant t(14;18) recombination in the BCL gene segments were investigated.

B) Heatmaps showing frequencies of DJ rearrangements in GFP^+^ NK cells at week 1, 2, and 3 of differentiation.

C) Total number of resolved and unresolved V and D rearrangements obtained at week 1, 2, and 3.

D) Mean number of nucleotide (nt) deletions at V, J, and 5’ and 3’ D ends at week 1, 2, and 3.

E) Average number of N1 and N2 nucleotide (nt) additions observed at week 1, 2, and 3.

F) Percentage of sequences with 0-16 and more (16+) N1 nucleotide additions observed in NK cells obtained at week 1, 2, and 3.

G) Percentage of sequences with 0-16 and more (16+) N2 nucleotide additions observed in NK cells obtained at week 1, 2, and 3.

H) Percentage of IGH sequences with indicated CDR3 length in nucleotides (nt) observed in sequences containing in frame (red) or out of frame (blue) rearrangements, or rearrangements leading to a stop codon (black). Results are shown for total NK cells obtained at week 1, 2, and 3.

Three independent experiments were performed, and results were calculated on three replicates of each sample. Statistical analysis was performed using 2Way ANOVA, or unpaired t test, respectively (*p<0.05, **p<0.01, ***p<0.001, ****p<0.0001).

**Figure S11: Quality of RNA-Seq data can be partially improved by filtering for gapped alignments.**

A) By filtering of data for gapped alignments (N tag based in CIGAR strings of mapping BAM files), ~2 % of the data (~300k reads per samples) were retained with similar results for samples of GFP^-^ (left) and GFP^+^ (right) NK cells.

B) Filtered reads show an improvement of read mapping events towards exonic regions while mapping to intergenic and intronic regions is slightly reduced. Similar observations were made in samples obtained from GFP^-^ and GFP^+^ NK cells, however more exonic read mapping events were observed in GFP^+^ versus GFP^-^ samples indicating a higher DNA contamination in GFP^-^ samples.

C) The strandedness of the reads did not change, because the NEBNext Single Cell /Low Input RNA Library Prep Kit used results in non-directional libraries.

D) Diversification of transcriptomic profiles obtained for GFP^-^ and GFP^+^ NK cells is displayed in a heatmap of sample distance. Three replicate samples were analyzed per time point from NK cell precursors at week 1, 2, and 3 of differentiation.

**Figure S12: RAG-fate mapped NK cells express differential transcription profiles at week 1, week 2, and week 3.**

A-C) Heatmaps showing the mean normalized gene counts of the top 100 differentially expressed genes in GFP^-^ and GFP^+^ NK cells obtained at week 1, 2, and 3. Relative expression is color coded as indicated by the legend underneath.

D-F) GO enrichment analysis was performed for NK cells obtained at week 1, 2, and 3 (FDR<0.05). GO terms related to gene expression (GE) and translation, metabolism, and cellular function are shown on the left. Enrichment is depicted on the X-axis; gene counts are shown by size, and adjusted p-values (-log10[p-value]) are color coded as indicated.

**Figure S13: Expression of genes involved in NK and B cell development and function.**

A) Expression of selected genes coding for NK cell markers that were differentially expressed between GFP^-^ and GFP^+^ cells are presented as box plots. Normalized gene counts obtained from GFP^-^ and GFP^+^ NK cells at week 1, 2, and 3, respectively, are shown by color coded symbols as indicated by the legend at the bottom right. Shown are means +/- SEM from 3 replicate values (n=3). Statistical analysis was performed using ordinary one-way ANOVA (*p<0.05, **p<0.01, ***p<0.001, ****p<0.0001).

B) Heatmap showing the mean normalized expression of selected genes involved in NK and B cell differentiation in GFP^-^ and GFP^+^ NK cells obtained at week 1, 2, and 3.

**Figure S14: Source Figures**

A) Uncropped source image of S1A. B) Uncropped, inverted source image of S1A.

C) Uncropped source image of S9A. D) Uncropped, inverted source image of S9A.

**Supplementary References**

1.Euchner J, Sprissler J, Cathomen T, Furst D, Schrezenmeier H, Debatin KM, et al. Natural Killer Cells Generated From Human Induced Pluripotent Stem Cells Mature to CD56(bright)CD16(+)NKp80(+/-)In-Vitro and Express KIR2DL2/DL3 and KIR3DL1. Front Immunol. 2021;12:640672.

2. Bryceson YT, March ME, Ljunggren HG, Long EO. Synergy among receptors on resting NK cells for the activation of natural cytotoxicity and cytokine secretion. Blood. 2006;107(1):159-66.

3. Chen Y, Cao J, Xiong M, Petersen AJ, Dong Y, Tao Y, et al. Engineering Human Stem Cell Lines with Inducible Gene Knockout using CRISPR/Cas9. Cell Stem Cell. 2015;17(2):233-44.

4. Bredemeyer AL, Helmink BA, Innes CL, Calderon B, McGinnis LM, Mahowald GK, et al. DNA double-strand breaks activate a multi-functional genetic program in developing lymphocytes. Nature. 2008;456(7223):819-23.

5. Ma Y, Pannicke U, Schwarz K, Lieber MR. Hairpin opening and overhang processing by an Artemis/DNA-dependent protein kinase complex in nonhomologous end joining and V(D)J recombination. Cell. 2002;108(6):781-94.

6. Pannicke U, Ma Y, Hopfner KP, Niewolik D, Lieber MR, Schwarz K. Functional and biochemical dissection of the structure-specific nuclease ARTEMIS. EMBO J. 2004;23(9):1987-97.

7. FastQC. 2015.

8. Liao Y, Smyth GK, Shi W. featureCounts: an efficient general purpose program for assigning sequence reads to genomic features. Bioinformatics. 2014;30(7):923-30.

9. Galaxy C. The Galaxy platform for accessible, reproducible and collaborative biomedical analyses: 2022 update. Nucleic Acids Res. 2022;50(W1):W345-W51.

10. Gene Ontology C, Aleksander SA, Balhoff J, Carbon S, Cherry JM, Drabkin HJ, et al. The Gene Ontology knowledgebase in 2023. Genetics. 2023;224(1).

11. Thomas PD, Ebert D, Muruganujan A, Mushayahama T, Albou LP, Mi H. PANTHER: Making genome-scale phylogenetics accessible to all. Protein Sci. 2022;31(1):8-22.

12. Ashburner M, Ball CA, Blake JA, Botstein D, Butler H, Cherry JM, et al. Gene ontology: tool for the unification of biology. The Gene Ontology Consortium. Nat Genet. 2000;25(1):25-9.

13. Ewels P, Magnusson M, Lundin S, Kaller M. MultiQC: summarize analysis results for multiple tools and samples in a single report. Bioinformatics. 2016;32(19):3047-8.

14. Ewels PA, Peltzer A, Fillinger S, Patel H, Alneberg J, Wilm A, et al. The nf-core framework for community-curated bioinformatics pipelines. Nat Biotechnol. 2020;38(3):276-8.

15. Love MI, Huber W, Anders S. Moderated estimation of fold change and dispersion for RNA-seq data with DESeq2. Genome Biol. 2014;15(12):550.

16. Wang L, Wang S, Li W. RSeQC: quality control of RNA-seq experiments. Bioinformatics. 2012;28(16):2184-5.

17. Tang D, Chen M, Huang X, Zhang G, Zeng L, Zhang G, et al. SRplot: A free online platform for data visualization and graphing. PLoS One. 2023;18(11):e0294236.

18. Dobin A, Davis CA, Schlesinger F, Drenkow J, Zaleski C, Jha S, et al. STAR: ultrafast universal RNA-seq aligner. Bioinformatics. 2013;29(1):15-21.

19. Krueger F. Trim Galore: a wrapper tool around Cutadapt and FastQC to consistently apply quality and adapter trimming to FastQ files, with some extra functionality for MspI-digested RRBS-type (Reduced Representation Bisufite-Seq) libraries. URL http://www bioinformatics babraham ac uk/projects/trim_galore/(Date of access: 28/04/2016). 2012:71.

20. Mali P, Yang L, Esvelt KM, Aach J, Guell M, DiCarlo JE, et al. RNA-guided human genome engineering via Cas9. Science. 2013;339(6121):823-6.

21. Sommeregger W, Prewein B, Reinhart D, Mader A, Kunert R. Transgene copy number comparison in recombinant mammalian cell lines: critical reflection of quantitative real-time PCR evaluation. Cytotechnology. 2013;65(5):811-8.

22. Kunz A, Gern U, Schmitt A, Neuber B, Wang L, Huckelhoven-Krauss A, et al. Optimized Assessment of qPCR-Based Vector Copy Numbers as a Safety Parameter for GMP-Grade CAR T Cells and Monitoring of Frequency in Patients. Mol Ther Methods Clin Dev. 2020;17:448-54.

23. Verwilt J, Trypsteen W, Van Paemel R, De Preter K, Giraldez MD, Mestdagh P, et al. When DNA gets in the way: A cautionary note for DNA contamination in extracellular RNA-seq studies. Proc Natl Acad Sci U S A. 2020;117(32):18934-6.
