# Supplementary material for: RAG recombinase expression discriminates the development of natural killer cells
Source: Front Immunol. 2025 Jul 25;16:1607664. doi: 10.3389/fimmu.2025.1607664 (PMC12331628; doi:10.3389/fimmu.2025.1607664)
Supplement: Supplementary file 2 [file Table2.pdf]

Analysis Type:	PANTHER Overrepresentation Test (Released 20240807)						
Annotation Version and Release Date:	GO Ontology database DOI: 10.5281/zenodo.12173881 Released 2024-06-17						
Analyzed List:	DEG all weeks (Homo sapiens)						
Reference List:	Background gene list.txt (Homo sapiens)						
Test Type:	FISHER						
Correction:	FDR		DEG all weeks	all weeks	DEG all weeks	DEG all weeks	DEG all weeks
GO biological process complete	Background gene list	DEG all weeks (89)	(expected)	(over/ under)	(fold Enrichment)	(raw P-value)	(FDR)
rRNA (guanine-N7)-methylation (GO:0070476)	2	2	0.02	+	> 100	6.76E-05	4.37E-02
cytoplasmic translation (GO:0002181)	123	16	1.02	+	15.74	2.72E-15	3.70E-11
positive regulation of cysteine-type endopeptidase activity involved in apoptotic process (GO:0043280)	81	6	0.67	+	8.96	5.31E-05	4.01E-02
oxidative phosphorylation (GO:0006119)	84	6	0.69	+	8.64	6.52E-05	4.43E-02
regulation of endopeptidase activity (GO:0052548)	172	12	1.42	+	8.44	1.54E-08	5.22E-05
positive regulation of endopeptidase activity (GO:0010950)	103	7	0.85	+	8.22	2.16E-05	2.10E-02
regulation of peptidase activity (GO:0052547)	191	12	1.58	+	7.6	4.95E-08	1.35E-04
positive regulation of peptidase activity (GO:0010952)	115	7	0.95	+	7.36	4.42E-05	3.53E-02
ATP metabolic process (GO:0046034)	121	7	1	+	7	6.12E-05	4.38E-02
translation (GO:0006412)	348	18	2.88	+	6.26	3.58E-10	2.44E-06
positive regulation of proteolysis (GO:0045862)	240	12	1.98	+	6.05	5.95E-07	1.01E-03
regulation of proteolysis (GO:0030162)	413	18	3.41	+	5.27	5.52E-09	2.50E-05
ribosome biogenesis (GO:0042254)	272	11	2.25	+	4.89	1.39E-05	1.58E-02
ribonucleoprotein complex biogenesis (GO:0022613)	400	14	3.31	+	4.23	4.57E-06	5.64E-03
regulation of hydrolase activity (GO:0051336)	445	15	3.68	+	4.08	3.18E-06	4.32E-03
regulation of catalytic activity (GO:0050790)	879	23	7.27	+	3.17	4.53E-07	8.80E-04
positive regulation of gene expression (GO:0010628)	748	18	6.18	+	2.91	3.24E-05	2.75E-02
organonitrogen compound biosynthetic process (GO:1901566)	970	23	8.02	+	2.87	2.52E-06	3.81E-03
regulation of molecular function (GO:0065009)	1220	28	10.08	+	2.78	2.70E-07	6.12E-04
gene expression (GO:0010467)	1843	32	15.23	+	2.1	2.42E-05	2.19E-02
protein metabolic process (GO:0019538)	2428	38	20.07	+	1.89	1.97E-05	2.06E-02

Analysis Type:	PANTHER Overrepresentation Test (Released 20240807)
Annotation Version and Release Date:	GO Ontology database DOI: 10.5281/zenodo.12173881 Released 2024-06-17
Analyzed List:	DEG week 1 (Homo sapiens)
Reference List:	Background gene list.txt (Homo sapiens)
Test Type:	FISHER
Correction:	FDR

GO biological process complete	Background gene list.txt	DEG week 1 (212)	DEG week 1 (expected)	DEG week 1 (over/under)	DEG week 1 (fold Enrichment)	DEG week 1 (raw P-value)	DEG week 1 (FDR)
formation of cytoplasmic translation initiation complex (GO:0001732)	15	5	0.3	+	16.93	7.22E-06	2.45E-03
cytoplasmic translation (GO:0002181)	123	27	2.42	+	11.15	3.62E-21	4.91E-17
proton motive force-driven mitochondrial ATP synthesis (GO:0042776)	47	10	0.93	+	10.81	1.94E-08	2.64E-05
proton motive force-driven ATP synthesis (GO:0015986)	51	10	1	+	9.96	4.48E-08	5.53E-05
cytoplasmic translational initiation (GO:0002183)	32	6	0.63	+	9.52	3.21E-05	9.27E-03
oxidative phosphorylation (GO:0006119)	84	15	1.65	+	9.07	7.06E-11	1.92E-07
ATP biosynthetic process (GO:0006754)	60	10	1.18	+	8.46	2.26E-07	1.92E-04
mitochondrial electron transport, NADH to ubiquinone (GO:0006120)	37	6	0.73	+	8.24	7.58E-05	2.06E-02
mitochondrial ATP synthesis coupled electron transport (GO:0042775)	66	10	1.3	+	7.7	5.71E-07	3.38E-04
ATP synthesis coupled electron transport (GO:0042773)	66	10	1.3	+	7.7	5.71E-07	3.24E-04
purine ribonucleoside triphosphate biosynthetic process (GO:0009206)	66	10	1.3	+	7.7	5.71E-07	3.11E-04
purine nucleoside triphosphate biosynthetic process (GO:0009145)	67	10	1.32	+	7.58	6.60E-07	3.45E-04
aerobic electron transport chain (GO:0019646)	62	9	1.22	+	7.37	3.08E-06	1.20E-03
ribonucleoside triphosphate biosynthetic process (GO:0009201)	71	10	1.4	+	7.15	1.15E-06	5.20E-04
nucleoside triphosphate biosynthetic process (GO:0009142)	77	10	1.52	+	6.6	2.46E-06	1.01E-03
proton transmembrane transport (GO:1902600)	85	11	1.67	+	6.57	7.93E-07	3.85E-04
translational initiation (GO:0006413)	62	8	1.22	+	6.55	2.69E-05	7.93E-03
aerobic respiration (GO:0009060)	124	16	2.44	+	6.55	2.42E-09	4.69E-06
respiratory electron transport chain (GO:0022904)	87	11	1.71	+	6.42	1.01E-06	4.71E-04
electron transport chain (GO:0022900)	93	11	1.83	+	6.01	1.97E-06	8.38E-04

cellular respiration (GO:0045333)	148	17	2.91	+	5.83	4.62E-09	7.86E-06
ATP metabolic process (GO:0046034)	121	13	2.38	+	5.46	6.99E-07	3.52E-04
translation (GO:0006412)	348	36	6.85	+	5.25	1.43E-16	9.69E-13
purine ribonucleoside triphosphate metabolic process (GO:0009205)	140	13	2.76	+	4.72	3.68E-06	1.39E-03
ribosomal small subunit biogenesis (GO:0042274)	97	9	1.91	+	4.71	1.20E-04	3.19E-02
energy derivation by oxidation of organic compounds (GO:0015980)	201	18	3.96	+	4.55	8.24E-08	8.00E-05
ribonucleoside triphosphate metabolic process (GO:0009199)	146	13	2.87	+	4.52	5.87E-06	2.16E-03
purine nucleoside triphosphate metabolic process (GO:0009144)	146	13	2.87	+	4.52	5.87E-06	2.10E-03
protein-RNA complex assembly (GO:0022618)	173	15	3.41	+	4.4	1.55E-06	6.80E-04
protein-RNA complex organization (GO:0071826)	181	15	3.56	+	4.21	2.74E-06	1.09E-03
nucleoside triphosphate metabolic process (GO:0009141)	159	13	3.13	+	4.15	1.49E-05	4.59E-03
regulation of endopeptidase activity (GO:0052548)	172	14	3.39	+	4.13	7.31E-06	2.42E-03
regulation of peptidase activity (GO:0052547)	191	14	3.76	+	3.72	2.41E-05	7.29E-03
generation of precursor metabolites and energy (GO:0006091)	274	20	5.4	+	3.71	4.44E-07	3.18E-04
ribonucleoprotein complex biogenesis (GO:0022613)	400	26	7.88	+	3.3	7.82E-08	8.17E-05
regulation of proteolysis (GO:0030162)	413	23	8.13	+	2.83	6.51E-06	2.27E-03
organonitrogen compound biosynthetic process (GO:1901566)	970	51	19.1	+	2.67	3.93E-11	1.34E-07
regulation of hydrolase activity (GO:0051336)	445	22	8.76	+	2.51	6.47E-05	1.79E-02
regulation of catalytic activity (GO:0050790)	879	37	17.31	+	2.14	9.87E-06	3.19E-03
gene expression (GO:0010467)	1843	77	36.29	+	2.12	1.54E-11	6.98E-08
protein-containing complex assembly (GO:0065003)	894	36	17.6	+	2.05	3.89E-05	1.10E-02
regulation of molecular function (GO:0065009)	1220	46	24.02	+	1.91	1.39E-05	4.39E-03
macromolecule biosynthetic process (GO:0009059)	2119	79	41.72	+	1.89	2.37E-09	5.37E-06
protein-containing complex organization (GO:0043933)	1462	49	28.79	+	1.7	1.51E-04	3.74E-02
biosynthetic process (GO:0009058)	2844	95	56	+	1.7	5.70E-09	8.61E-06
protein metabolic process (GO:0019538)	2428	80	47.81	+	1.67	4.67E-07	3.17E-04
cellular biosynthetic process (GO:0044249)	2536	83	49.93	+	1.66	3.02E-07	2.41E-04
organonitrogen compound metabolic process (GO:1901564)	3156	97	62.14	+	1.56	3.67E-07	2.77E-04
cellular metabolic process (GO:0044237)	3960	117	77.97	+	1.5	4.81E-08	5.45E-05
macromolecule metabolic process (GO:0043170)	3880	112	76.4	+	1.47	5.00E-07	3.24E-04

primary metabolic process (GO:0044238)	4762	130	93.76	+	1.39	5.64E-07	3.48E-04
metabolic process (GO:0008152)	5305	142	104.45	+	1.36	2.16E-07	1.96E-04
cellular process (GO:0009987)	8581	190	168.96	+	1.12	1.34E-04	3.51E-02
biological_process (GO:0008150)	9983	209	196.56	+	1.06	1.51E-04	3.87E-02
Unclassified (UNCLASSIFIED)	784	3	15.44	-	0.19	1.51E-04	3.79E-02

Analysis Type:	PANTHER Overrepresentation Test (Released 20240807)						
Annotation Version and Release Date:	GO Ontology database DOI: 10.5281/zenodo.12173881 Released 2024-06-17						
Analyzed List:	DEG week 2 (Homo sapiens)						
Reference List:	Background gene list.txt (Homo sapiens)						
Test Type:	FISHER						
Correction:	FDR		DEG week 2	DEG week 2	DEG week 2	DEG week 2	DEG week 2
GO biological process complete	Background gene list.txt	DEG week 2 (379)	(expected)	(over/ under)	(fold Enrichment)	(raw P-value)	(FDR)
7-methylguanosine cap hypermethylation (GO:0036261)	8	6	0.28	+	21.31	4.83E-08	1.13E-05
post-translational protein targeting to membrane, translocation (GO:0031204)	7	4	0.25	+	16.23	4.86E-05	8.06E-03
mitochondrial electron transport, cytochrome c to oxygen (GO:0006123)	13	7	0.46	+	15.3	9.06E-08	2.02E-05
cytoplasmic translation (GO:0002181)	123	60	4.33	+	13.86	5.91E-55	8.04E-51
ribosomal small subunit assembly (GO:0000028)	17	8	0.6	+	13.37	4.03E-08	9.61E-06
RNA capping (GO:0036260)	14	6	0.49	+	12.18	4.32E-06	8.51E-04
U2-type prespliceosome assembly (GO:1903241)	24	9	0.84	+	10.65	6.18E-08	1.42E-05
proton motive force-driven ATP synthesis (GO:0015986)	51	16	1.8	+	8.91	9.45E-12	3.78E-09
aerobic electron transport chain (GO:0019646)	62	19	2.18	+	8.71	1.66E-13	1.07E-10
mitochondrial ATP synthesis coupled electron transport (GO:0042775)	66	20	2.32	+	8.61	4.79E-14	3.83E-11
ATP synthesis coupled electron transport (GO:0042773)	66	20	2.32	+	8.61	4.79E-14	3.62E-11
proton motive force-driven mitochondrial ATP synthesis (GO:0042776)	47	14	1.65	+	8.46	4.19E-10	1.27E-07
oxidative phosphorylation (GO:0006119)	84	25	2.96	+	8.46	4.85E-17	5.50E-14
mitochondrial electron transport, NADH to ubiquinone (GO:0006120)	37	10	1.3	+	7.68	3.86E-07	8.20E-05
ATP biosynthetic process (GO:0006754)	60	16	2.11	+	7.58	1.47E-10	5.01E-08
ribosome assembly (GO:0042255)	53	14	1.87	+	7.5	2.43E-09	6.48E-07
respiratory electron transport chain (GO:0022904)	87	22	3.06	+	7.18	1.61E-13	1.10E-10
purine ribonucleoside triphosphate biosynthetic process (GO:0009206)	66	16	2.32	+	6.89	6.94E-10	2.05E-07
purine nucleoside triphosphate biosynthetic process (GO:0009145)	67	16	2.36	+	6.78	8.83E-10	2.50E-07
ribosomal small subunit biogenesis (GO:0042274)	97	23	3.41	+	6.74	1.99E-13	1.23E-10
electron transport chain (GO:0022900)	93	22	3.27	+	6.72	7.07E-13	4.01E-10
ribonucleoside triphosphate biosynthetic process (GO:0009201)	71	16	2.5	+	6.4	2.21E-09	6.01E-07

antimicrobial humoral immune response mediated by antimicrobial peptide (GO:0061844)	40	9	1.41	+	6.39	7.83E-06	1.48E-03
RNA 5'-end processing (GO:0000966)	32	7	1.13	+	6.21	9.95E-05	1.47E-02
aerobic respiration (GO:0009060)	124	26	4.36	+	5.96	1.17E-13	8.38E-11
nucleoside triphosphate biosynthetic process (GO:0009142)	77	16	2.71	+	5.9	7.79E-09	2.00E-06
translation (GO:0006412)	348	70	12.25	+	5.71	2.92E-34	1.99E-30
spliceosomal snRNP assembly (GO:0000387)	38	7	1.34	+	5.23	3.11E-04	3.99E-02
cellular respiration (GO:0045333)	148	27	5.21	+	5.18	1.35E-12	6.78E-10
spliceosomal complex assembly (GO:0000245)	66	12	2.32	+	5.17	2.68E-06	5.37E-04
antimicrobial humoral response (GO:0019730)	55	10	1.94	+	5.17	1.84E-05	3.38E-03
NADH dehydrogenase complex assembly (GO:0010257)	52	9	1.83	+	4.92	7.25E-05	1.13E-02
mitochondrial respiratory chain complex I assembly (GO:0032981)	52	9	1.83	+	4.92	7.25E-05	1.12E-02
protein-RNA complex assembly (GO:0022618)	173	29	6.09	+	4.76	1.80E-12	8.75E-10
cellular response to type II interferon (GO:0071346)	66	11	2.32	+	4.73	1.67E-05	3.12E-03
ribosomal large subunit biogenesis (GO:0042273)	60	10	2.11	+	4.73	4.06E-05	6.89E-03
protein-RNA complex organization (GO:0071826)	181	30	6.37	+	4.71	9.97E-13	5.21E-10
ATP metabolic process (GO:0046034)	121	20	4.26	+	4.7	7.02E-09	1.83E-06
maturation of SSU-rRNA (GO:0030490)	49	8	1.72	+	4.64	2.79E-04	3.64E-02
response to type II interferon (GO:0034341)	83	13	2.92	+	4.45	5.87E-06	1.14E-03
rRNA processing (GO:0006364)	187	29	6.58	+	4.41	1.33E-11	5.04E-09
ribosome biogenesis (GO:0042254)	272	42	9.57	+	4.39	3.01E-16	2.92E-13
proton transmembrane transport (GO:1902600)	85	13	2.99	+	4.34	7.69E-06	1.47E-03
mitochondrial respiratory chain complex assembly (GO:0033108)	80	12	2.82	+	4.26	2.11E-05	3.77E-03
mRNA splicing, via spliceosome (GO:0000398)	215	32	7.57	+	4.23	3.49E-12	1.53E-09
RNA splicing, via transesterification reactions with bulged adenosine as nucleophile (GO:0000377)	215	32	7.57	+	4.23	3.49E-12	1.48E-09
RNA splicing, via transesterification reactions (GO:0000375)	219	32	7.71	+	4.15	5.81E-12	2.39E-09
ribonucleoprotein complex biogenesis (GO:0022613)	400	58	14.08	+	4.12	8.37E-21	1.90E-17
RNA methylation (GO:0001510)	63	9	2.22	+	4.06	3.32E-04	4.21E-02
purine ribonucleoside triphosphate metabolic process (GO:0009205)	140	20	4.93	+	4.06	8.90E-08	2.02E-05
energy derivation by oxidation of organic compounds (GO:0015980)	201	28	7.08	+	3.96	4.02E-10	1.24E-07
rRNA metabolic process (GO:0016072)	211	29	7.43	+	3.9	2.68E-10	8.68E-08
ribonucleoside triphosphate metabolic process (GO:0009199)	146	20	5.14	+	3.89	1.80E-07	3.95E-05

purine nucleoside triphosphate metabolic process (GO:0009144)	146	20	5.14	+	3.89	1.80E-07	3.89E-05
nucleoside triphosphate metabolic process (GO:0009141)	159	20	5.6	+	3.57	7.33E-07	1.53E-04
RNA splicing (GO:0008380)	319	40	11.23	+	3.56	1.85E-12	8.65E-10
generation of precursor metabolites and energy (GO:0006091)	274	32	9.64	+	3.32	2.11E-09	5.86E-07
purine ribonucleotide biosynthetic process (GO:0009152)	137	16	4.82	+	3.32	2.45E-05	4.32E-03
positive regulation of endopeptidase activity (GO:0010950)	103	12	3.63	+	3.31	2.58E-04	3.44E-02
mRNA processing (GO:0006397)	367	40	12.92	+	3.1	1.53E-10	5.08E-08
ribonucleotide biosynthetic process (GO:0009260)	150	16	5.28	+	3.03	7.47E-05	1.14E-02
regulation of endopeptidase activity (GO:0052548)	172	18	6.05	+	2.97	3.47E-05	6.04E-03
purine nucleotide biosynthetic process (GO:0006164)	163	17	5.74	+	2.96	5.96E-05	9.42E-03
RNA processing (GO:0006396)	696	72	24.5	+	2.94	3.54E-17	4.38E-14
ribose phosphate biosynthetic process (GO:0046390)	157	16	5.53	+	2.9	1.29E-04	1.86E-02
purine-containing compound biosynthetic process (GO:0072522)	169	17	5.95	+	2.86	9.38E-05	1.40E-02
organonitrogen compound biosynthetic process (GO:1901566)	970	96	34.14	+	2.81	1.07E-21	2.91E-18
mRNA metabolic process (GO:0016071)	495	47	17.42	+	2.7	3.97E-10	1.25E-07
regulation of peptidase activity (GO:0052547)	191	18	6.72	+	2.68	1.36E-04	1.95E-02
positive regulation of proteolysis (GO:0045862)	240	22	8.45	+	2.6	3.84E-05	6.61E-03
non-membrane-bounded organelle assembly (GO:0140694)	271	23	9.54	+	2.41	1.39E-04	1.96E-02
gene expression (GO:0010467)	1843	150	64.87	+	2.31	3.75E-26	1.70E-22
regulation of translation (GO:0006417)	310	25	10.91	+	2.29	1.09E-04	1.60E-02
RNA biosynthetic process (GO:0032774)	1053	83	37.07	+	2.24	8.42E-13	4.58E-10
nucleobase-containing compound biosynthetic process (GO:0034654)	1322	100	46.53	+	2.15	2.63E-14	2.23E-11
post-transcriptional regulation of gene expression (GO:0010608)	411	31	14.47	+	2.14	5.63E-05	9.11E-03
RNA metabolic process (GO:0016070)	1178	88	41.47	+	2.12	3.39E-12	1.54E-09
nucleic acid biosynthetic process (GO:0141187)	1114	83	39.21	+	2.12	2.19E-11	8.06E-09
macromolecule biosynthetic process (GO:0009059)	2119	155	74.59	+	2.08	5.69E-22	1.93E-18
regulation of proteolysis (GO:0030162)	413	30	14.54	+	2.06	1.73E-04	2.42E-02
protein-containing complex assembly (GO:0065003)	894	64	31.47	+	2.03	3.49E-08	8.47E-06
defense response to symbiont (GO:0140546)	541	38	19.04	+	2	5.66E-05	9.05E-03
positive regulation of catalytic activity (GO:0043085)	541	36	19.04	+	1.89	2.63E-04	3.47E-02
nucleic acid metabolic process (GO:0090304)	1647	109	57.97	+	1.88	1.04E-11	4.06E-09
defense response to other organism (GO:0098542)	605	39	21.3	+	1.83	2.34E-04	3.25E-02

biosynthetic process (GO:0009058)	2844	183	100.11	+	1.83	1.79E-20	3.47E-17
cellular biosynthetic process (GO:0044249)	2536	163	89.27	+	1.83	2.04E-17	2.77E-14
nucleobase-containing compound metabolic process (GO:0006139)	2052	131	72.23	+	1.81	4.26E-13	2.52E-10
immune response (GO:0006955)	829	52	29.18	+	1.78	4.65E-05	7.80E-03
regulation of catalytic activity (GO:0050790)	879	54	30.94	+	1.75	5.02E-05	8.22E-03
cellular component biogenesis (GO:0044085)	1875	114	66	+	1.73	7.85E-10	2.27E-07
response to other organism (GO:0051707)	822	49	28.93	+	1.69	2.39E-04	3.28E-02
response to external biotic stimulus (GO:0043207)	823	49	28.97	+	1.69	2.43E-04	3.30E-02
protein metabolic process (GO:0019538)	2428	142	85.47	+	1.66	2.85E-11	1.02E-08
protein-containing complex organization (GO:0043933)	1462	85	51.46	+	1.65	1.78E-06	3.62E-04
cellular metabolic process (GO:0044237)	3960	224	139.39	+	1.61	3.82E-19	6.48E-16
macromolecule metabolic process (GO:0043170)	3880	214	136.58	+	1.57	1.89E-16	1.98E-13
organonitrogen compound metabolic process (GO:1901564)	3156	170	111.09	+	1.53	8.07E-11	2.81E-08
cellular component assembly (GO:0022607)	1658	87	58.36	+	1.49	8.35E-05	1.26E-02
metabolic process (GO:0008152)	5305	269	186.74	+	1.44	3.15E-18	4.76E-15
primary metabolic process (GO:0044238)	4762	241	167.62	+	1.44	1.67E-14	1.51E-11
cellular component organization or biogenesis (GO:0071840)	3857	170	135.77	+	1.25	2.49E-04	3.36E-02
cellular process (GO:0009987)	8581	338	302.05	+	1.12	9.04E-07	1.86E-04
biological_process (GO:0008150)	9983	375	351.4	+	1.07	1.55E-08	3.83E-06
lipid metabolic process (GO:0006629)	748	10	26.33	-	0.38	2.79E-04	3.61E-02
animal organ morphogenesis (GO:0009887)	509	3	17.92	-	0.17	1.99E-05	3.61E-03
Unclassified (UNCLASSIFIED)	784	4	27.6	-	0.14	1.55E-08	3.90E-06

Analysis Type:	PANTHER Overrepresentation Test (Released 20240807)
Annotation Version and Release Date:	GO Ontology database DOI: 10.5281/zenodo.12173881 Released 2024-06-17
Analyzed List:	DEG week 3 (Homo sapiens)
Reference List:	Background gene list.txt (Homo sapiens)
Test Type:	FISHER
Correction:	FDR

GO biological process complete	Background gene list.txt	DEG week 3 (420)	DEG week 3 (expected)	DEG week 3 (over/under)	DEG week 3 (fold Enrichment)	DEG week 3 (raw P-value)	DEG week 3 (FDR)
cytoplasmic translation (GO:0002181)	123	47	4.8	+	9.8	8.23E-35	1.12E-30
translation (GO:0006412)	348	58	13.57	+	4.27	1.28E-21	8.69E-18
gene expression (GO:0010467)	1843	147	71.89	+	2.04	1.78E-19	8.06E-16
macromolecule metabolic process (GO:0043170)	3880	239	151.35	+	1.58	9.09E-19	3.09E-15
macromolecule biosynthetic process (GO:0009059)	2119	153	82.66	+	1.85	3.24E-16	8.81E-13
metabolic process (GO:0008152)	5305	287	206.94	+	1.39	1.15E-15	2.61E-12
primary metabolic process (GO:0044238)	4762	264	185.76	+	1.42	6.11E-15	1.19E-11
ribonucleoprotein complex biogenesis (GO:0022613)	400	51	15.6	+	3.27	4.12E-14	7.00E-11
protein metabolic process (GO:0019538)	2428	161	94.71	+	1.7	1.10E-13	1.66E-10
biosynthetic process (GO:0009058)	2844	176	110.94	+	1.59	2.25E-12	2.78E-09
protein-RNA complex organization (GO:0071826)	181	31	7.06	+	4.39	2.53E-12	2.87E-09
organonitrogen compound metabolic process (GO:1901564)	3156	190	123.11	+	1.54	2.24E-12	3.04E-09
protein-RNA complex assembly (GO:0022618)	173	30	6.75	+	4.45	4.16E-12	4.35E-09
cellular biosynthetic process (GO:0044249)	2536	159	98.92	+	1.61	2.15E-11	2.09E-08
organonitrogen compound biosynthetic process (GO:1901566)	970	80	37.84	+	2.11	7.87E-11	7.13E-08
nucleobase-containing compound metabolic process (GO:0006139)	2052	133	80.04	+	1.66	3.35E-10	2.84E-07
cellular metabolic process (GO:0044237)	3960	216	154.47	+	1.4	4.56E-10	3.64E-07
RNA splicing, via transesterification reactions with bulged adenosine as nucleophile (GO:0000377)	215	30	8.39	+	3.58	1.08E-09	7.70E-07
mRNA splicing, via spliceosome (GO:0000398)	215	30	8.39	+	3.58	1.08E-09	8.12E-07
RNA splicing, via transesterification reactions (GO:0000375)	219	30	8.54	+	3.51	1.69E-09	1.09E-06
RNA processing (GO:0006396)	696	61	27.15	+	2.25	1.63E-09	1.11E-06
nucleobase-containing compound biosynthetic process (GO:0034654)	1322	94	51.57	+	1.82	3.29E-09	2.03E-06

ribosome biogenesis (GO:0042254)	272	33	10.61	+	3.11	5.90E-09	3.49E-06
nucleic acid metabolic process (GO:0090304)	1647	109	64.25	+	1.7	9.74E-09	5.52E-06
RNA biosynthetic process (GO:0032774)	1053	78	41.08	+	1.9	1.92E-08	1.04E-05
RNA metabolic process (GO:0016070)	1178	84	45.95	+	1.83	2.54E-08	1.33E-05
nucleic acid biosynthetic process (GO:0141187)	1114	80	43.46	+	1.84	4.59E-08	2.31E-05
RNA splicing (GO:0008380)	319	34	12.44	+	2.73	9.05E-08	4.24E-05
7-methylguanosine cap hypermethylation (GO:0036261)	8	6	0.31	+	19.23	8.91E-08	4.33E-05
purine ribonucleoside triphosphate metabolic process (GO:0009205)	140	21	5.46	+	3.85	1.03E-07	4.65E-05
cellular component biogenesis (GO:0044085)	1875	116	73.14	+	1.59	1.19E-07	5.22E-05
U2-type prespliceosome assembly (GO:1903241)	24	9	0.94	+	9.61	1.49E-07	6.33E-05
ribosomal small subunit biogenesis (GO:0042274)	97	17	3.78	+	4.49	1.74E-07	7.15E-05
ATP metabolic process (GO:0046034)	121	19	4.72	+	4.03	2.01E-07	7.79E-05
purine nucleoside triphosphate metabolic process (GO:0009144)	146	21	5.7	+	3.69	2.14E-07	7.85E-05
positive regulation of proteolysis (GO:0045862)	240	28	9.36	+	2.99	2.00E-07	8.00E-05
ribonucleoside triphosphate metabolic process (GO:0009199)	146	21	5.7	+	3.69	2.14E-07	8.07E-05
regulation of proteolysis (GO:0030162)	413	39	16.11	+	2.42	3.10E-07	1.11E-04
proteolysis involved in protein catabolic process (GO:0051603)	484	43	18.88	+	2.28	3.94E-07	1.37E-04
ribosome assembly (GO:0042255)	53	12	2.07	+	5.8	6.62E-07	2.25E-04
macromolecule catabolic process (GO:0009057)	757	58	29.53	+	1.96	7.21E-07	2.39E-04
protein-containing complex assembly (GO:0065003)	894	65	34.87	+	1.86	7.73E-07	2.50E-04
chaperone-mediated protein folding (GO:0061077)	64	13	2.5	+	5.21	8.61E-07	2.72E-04
nucleoside triphosphate metabolic process (GO:0009141)	159	21	6.2	+	3.39	9.13E-07	2.76E-04
positive regulation of protein metabolic process (GO:0051247)	836	62	32.61	+	1.9	8.95E-07	2.76E-04
proteasomal protein catabolic process (GO:0010498)	321	32	12.52	+	2.56	9.89E-07	2.92E-04
purine ribonucleoside triphosphate biosynthetic process (GO:0009206)	66	13	2.57	+	5.05	1.25E-06	3.61E-04
proteasome-mediated ubiquitin-dependent protein catabolic process (GO:0043161)	248	27	9.67	+	2.79	1.32E-06	3.73E-04
regulation of protein metabolic process (GO:0051246)	1473	93	57.46	+	1.62	1.44E-06	3.99E-04
purine nucleoside triphosphate biosynthetic process (GO:0009145)	67	13	2.61	+	4.97	1.50E-06	4.06E-04
mRNA metabolic process (GO:0016071)	495	42	19.31	+	2.18	1.99E-06	5.29E-04
mRNA processing (GO:0006397)	367	34	14.32	+	2.37	2.76E-06	7.07E-04
ATP biosynthetic process (GO:0006754)	60	12	2.34	+	5.13	2.71E-06	7.09E-04

ribonucleoside triphosphate biosynthetic process (GO:0009201)	71	13	2.77	+	4.69	2.97E-06	7.48E-04
Unclassified (UNCLASSIFIED)	784	9	30.58	-	0.29	3.27E-06	7.79E-04
biological_process (GO:0008150)	9983	411	389.42	+	1.06	3.27E-06	7.92E-04
proton motive force-driven ATP synthesis (GO:0015986)	51	11	1.99	+	5.53	3.25E-06	8.02E-04
regulation of translation (GO:0006417)	310	30	12.09	+	2.48	4.00E-06	9.06E-04
oxidative phosphorylation (GO:0006119)	84	14	3.28	+	4.27	3.97E-06	9.16E-04
protein-containing complex organization (GO:0043933)	1462	91	57.03	+	1.6	3.93E-06	9.22E-04
platelet aggregation (GO:0070527)	34	9	1.33	+	6.79	4.22E-06	9.41E-04
regulation of proteolysis involved in protein catabolic process (GO:1903050)	178	21	6.94	+	3.02	5.74E-06	1.26E-03
protein catabolic process (GO:0030163)	570	45	22.23	+	2.02	5.85E-06	1.26E-03
RNA capping (GO:0036260)	14	6	0.55	+	10.99	7.82E-06	1.56E-03
positive regulation of translation (GO:0045727)	113	16	4.41	+	3.63	7.41E-06	1.57E-03
positive regulation of telomerase RNA localization to Cajal body (GO:1904874)	14	6	0.55	+	10.99	7.82E-06	1.59E-03
nucleoside triphosphate biosynthetic process (GO:0009142)	77	13	3	+	4.33	7.59E-06	1.59E-03
regulation of post-translational protein modification (GO:1901873)	196	22	7.65	+	2.88	7.80E-06	1.61E-03
formation of cytoplasmic translation initiation complex (GO:0001732)	15	6	0.59	+	10.25	1.26E-05	2.41E-03
post-transcriptional regulation of gene expression (GO:0010608)	411	35	16.03	+	2.18	1.22E-05	2.41E-03
rRNA processing (GO:0006364)	187	21	7.29	+	2.88	1.24E-05	2.42E-03
modification-dependent protein catabolic process (GO:0019941)	395	34	15.41	+	2.21	1.39E-05	2.62E-03
regulation of protein stability (GO:0031647)	250	25	9.75	+	2.56	1.47E-05	2.74E-03
modification-dependent macromolecule catabolic process (GO:0043632)	397	34	15.49	+	2.2	1.51E-05	2.77E-03
positive regulation of establishment of protein localization to telomere (GO:1904851)	10	5	0.39	+	12.82	1.89E-05	3.42E-03
regulation of protein modification by small protein conjugation or removal (GO:1903320)	193	21	7.53	+	2.79	2.02E-05	3.61E-03
non-membrane-bounded organelle assembly (GO:0140694)	271	26	10.57	+	2.46	2.07E-05	3.65E-03
homotypic cell-cell adhesion (GO:0034109)	41	9	1.6	+	5.63	2.21E-05	3.86E-03
regulation of ubiquitin-protein transferase activity (GO:0051438)	32	8	1.25	+	6.41	2.31E-05	3.98E-03
negative regulation of RNA splicing (GO:0033119)	24	7	0.94	+	7.48	2.54E-05	4.32E-03
regulation of telomerase RNA localization to Cajal body (GO:1904872)	17	6	0.66	+	9.05	2.92E-05	4.89E-03
positive regulation of establishment of protein localization (GO:1904951)	229	23	8.93	+	2.57	3.01E-05	5.00E-03

regulation of protein localization to Cajal body (GO:1904869)	11	5	0.43	+	11.65	3.35E-05	5.36E-03
positive regulation of protein localization to Cajal body (GO:1904871)	11	5	0.43	+	11.65	3.35E-05	5.43E-03
regulation of establishment of protein localization to telomere (GO:0070203)	11	5	0.43	+	11.65	3.35E-05	5.49E-03
regulation of protein ubiquitination (GO:0031396)	156	18	6.09	+	2.96	3.60E-05	5.70E-03
spliceosomal complex assembly (GO:0000245)	66	11	2.57	+	4.27	4.32E-05	6.67E-03
cellular component assembly (GO:0022607)	1658	96	64.68	+	1.48	4.29E-05	6.70E-03
proteolysis (GO:0006508)	788	54	30.74	+	1.76	4.88E-05	7.45E-03
positive regulation of protein localization to chromosome, telomeric region (GO:1904816)	12	5	0.47	+	10.68	5.57E-05	8.31E-03
regulation of establishment of protein localization to chromosome (GO:0070202)	12	5	0.47	+	10.68	5.57E-05	8.40E-03
ribosomal large subunit assembly (GO:0000027)	19	6	0.74	+	8.1	5.98E-05	8.84E-03
ubiquitin-dependent protein catabolic process (GO:0006511)	385	32	15.02	+	2.13	6.80E-05	9.94E-03
proton motive force-driven mitochondrial ATP synthesis (GO:0042776)	47	9	1.83	+	4.91	6.99E-05	1.01E-02
rRNA metabolic process (GO:0016072)	211	21	8.23	+	2.55	7.60E-05	1.09E-02
regulation of protein localization to chromosome, telomeric region (GO:1904814)	13	5	0.51	+	9.86	8.75E-05	1.24E-02
aerobic respiration (GO:0009060)	124	15	4.84	+	3.1	9.32E-05	1.31E-02
proton transmembrane transport (GO:1902600)	85	12	3.32	+	3.62	1.05E-04	1.46E-02
positive regulation of signal transduction by p53 class mediator (GO:1901798)	21	6	0.82	+	7.32	1.12E-04	1.54E-02
lipid metabolic process (GO:0006629)	748	11	29.18	-	0.38	1.15E-04	1.56E-02
cellular process (GO:0009987)	8581	365	334.73	+	1.09	1.17E-04	1.58E-02
regulation of catalytic activity (GO:0050790)	879	57	34.29	+	1.66	1.19E-04	1.59E-02
positive regulation of establishment of protein localization to mitochondrion (GO:1903749)	30	7	1.17	+	5.98	1.22E-04	1.61E-02
aerobic electron transport chain (GO:0019646)	62	10	2.42	+	4.13	1.28E-04	1.65E-02
translational initiation (GO:0006413)	62	10	2.42	+	4.13	1.28E-04	1.67E-02
regulation of endopeptidase activity (GO:0052548)	172	18	6.71	+	2.68	1.30E-04	1.67E-02
respiratory electron transport chain (GO:0022904)	87	12	3.39	+	3.54	1.33E-04	1.68E-02
catabolic process (GO:0009056)	1408	82	54.92	+	1.49	1.54E-04	1.93E-02
negative regulation of mRNA metabolic process (GO:1903312)	64	10	2.5	+	4.01	1.68E-04	2.08E-02
organonitrogen compound catabolic process (GO:1901565)	829	54	32.34	+	1.67	1.67E-04	2.08E-02

protein folding (GO:0006457)	176	18	6.87	+	2.62	1.74E-04	2.13E-02
lipid biosynthetic process (GO:0008610)	401	3	15.64	-	0.19	1.77E-04	2.15E-02
positive regulation of molecular function (GO:0044093)	752	50	29.33	+	1.7	1.82E-04	2.19E-02
RNA 5'-end processing (GO:0000966)	32	7	1.25	+	5.61	1.89E-04	2.23E-02
cytoplasmic translational initiation (GO:0002183)	32	7	1.25	+	5.61	1.89E-04	2.25E-02
cellular respiration (GO:0045333)	148	16	5.77	+	2.77	2.09E-04	2.45E-02
ATP synthesis coupled electron transport (GO:0042773)	66	10	2.57	+	3.88	2.18E-04	2.51E-02
regulation of establishment of protein localization (GO:0070201)	358	29	13.96	+	2.08	2.21E-04	2.52E-02
mitochondrial ATP synthesis coupled electron transport (GO:0042775)	66	10	2.57	+	3.88	2.18E-04	2.54E-02
regulation of deoxyribonuclease activity (GO:0032070)	9	4	0.35	+	11.39	2.46E-04	2.79E-02
electron transport chain (GO:0022900)	93	12	3.63	+	3.31	2.52E-04	2.83E-02
protein stabilization (GO:0050821)	166	17	6.48	+	2.63	2.58E-04	2.87E-02
regulation of proteasomal protein catabolic process (GO:0061136)	151	16	5.89	+	2.72	2.64E-04	2.92E-02
regulation of molecular function (GO:0065009)	1220	72	47.59	+	1.51	2.83E-04	3.10E-02
cellular lipid metabolic process (GO:0044255)	593	8	23.13	-	0.35	2.85E-04	3.10E-02
regulation of RNA splicing (GO:0043484)	138	15	5.38	+	2.79	3.09E-04	3.34E-02
regulation of mRNA metabolic process (GO:1903311)	227	21	8.85	+	2.37	3.22E-04	3.45E-02
ribosomal small subunit assembly (GO:0000028)	17	5	0.66	+	7.54	3.70E-04	3.87E-02
positive regulation of catalytic activity (GO:0043085)	541	38	21.1	+	1.8	3.73E-04	3.87E-02
regulation of nuclease activity (GO:0032069)	17	5	0.66	+	7.54	3.70E-04	3.90E-02
viral translation (GO:0019081)	17	5	0.66	+	7.54	3.70E-04	3.93E-02
organelle organization (GO:0006996)	2164	114	84.41	+	1.35	3.85E-04	3.96E-02
substantia nigra development (GO:0021762)	36	7	1.4	+	4.98	4.09E-04	4.15E-02
cellular component organization or biogenesis (GO:0071840)	3857	185	150.45	+	1.23	4.07E-04	4.16E-02
regulation of catabolic process (GO:0009894)	758	49	29.57	+	1.66	4.18E-04	4.21E-02
negative regulation of protein-containing complex assembly (GO:0031333)	99	12	3.86	+	3.11	4.53E-04	4.53E-02
regulation of peptidase activity (GO:0052547)	191	18	7.45	+	2.42	4.78E-04	4.74E-02
negative regulation of cellular component organization (GO:0051129)	492	35	19.19	+	1.82	5.00E-04	4.85E-02
positive regulation of proteolysis involved in protein catabolic process (GO:1903052)	100	12	3.9	+	3.08	4.97E-04	4.86E-02
regulation of protein neddylation (GO:2000434)	18	5	0.7	+	7.12	4.96E-04	4.88E-02

Analysis Type:	PANTHER Overrepresentation Test (Released 20240807)						
Annotation Version and Release Date:	Reactome version 86 Released 2023-09-07						
Analyzed List:	DEG week 1 (Homo sapiens)						
Reference List:	Background gene list.txt (Homo sapiens)						
Test Type:	FISHER						
Correction:	FDR		DEG week 1	DEG week	DEG week 1	DEG week 1	DEG week 1
Reactome pathways	Background gene list.txt	DEG week 1 (212)	(expected)	(over/ under)	(fold Enrichment)	(raw P-value)	(FDR)
Formation of ATP by chemiosmotic coupling (R-HSA-163210)	9	5	0.18	+	28.22	3.34E-07	1.50E-05
SARS-CoV-1 modulates host translation machinery (R-HSA-9735869)	35	11	0.69	+	15.96	3.67E-11	2.21E-09
Protein methylation (R-HSA-8876725)	13	4	0.26	+	15.63	9.09E-05	1.60E-03
Formation of the ternary complex, and subsequently, the 43S complex (R-HSA-72695)	50	15	0.98	+	15.24	1.93E-14	1.26E-12
Formation of a pool of free 40S subunits (R-HSA-72689)	99	29	1.95	+	14.88	1.31E-26	1.61E-23
Eukaryotic Translation Elongation (R-HSA-156842)	90	26	1.77	+	14.67	9.20E-24	3.24E-21
Peptide chain elongation (R-HSA-156902)	87	25	1.71	+	14.59	8.10E-23	2.50E-20
Ribosomal scanning and start codon recognition (R-HSA-72702)	56	16	1.1	+	14.51	6.02E-15	4.24E-13
Translation initiation complex formation (R-HSA-72649)	56	16	1.1	+	14.51	6.02E-15	4.12E-13
subsequent binding to 43S (R-HSA-72662)	57	16	1.12	+	14.26	8.22E-15	5.48E-13
Eukaryotic Translation Termination (R-HSA-72764)	90	25	1.77	+	14.11	2.07E-22	5.67E-20
L13a-mediated translational silencing of Ceruloplasmin expression (R-HSA-156827)	108	30	2.13	+	14.11	9.81E-27	2.42E-23
Viral mRNA Translation (R-HSA-192823)	87	24	1.71	+	14.01	1.80E-21	3.70E-19
GTP hydrolysis and joining of the 60S ribosomal subunit (R-HSA-72706)	109	30	2.15	+	13.98	1.33E-26	1.09E-23
Selenocysteine synthesis (R-HSA-2408557)	90	24	1.77	+	13.54	4.39E-21	7.21E-19
(EJC) (R-HSA-975956)	92	24	1.81	+	13.25	7.79E-21	1.13E-18
Cap-dependent Translation Initiation (R-HSA-72737)	116	30	2.28	+	13.13	1.03E-25	6.34E-23
Eukaryotic Translation Initiation (R-HSA-72613)	116	30	2.28	+	13.13	1.03E-25	5.07E-23
Response of EIF2AK4 (GCN2) to amino acid deficiency (R-HSA-9633012)	94	24	1.85	+	12.97	1.36E-20	1.87E-18
Endosomal/Vacuolar pathway (R-HSA-1236977)	12	3	0.24	+	12.7	1.45E-03	1.99E-02
SARS-CoV-2 modulates host translation machinery (R-HSA-9754678)	48	12	0.95	+	12.7	9.30E-11	5.46E-09
HSF1 activation (R-HSA-3371511)	12	3	0.24	+	12.7	1.45E-03	1.98E-02

Cristae formation (R-HSA-8949613)	21	5	0.41	+	12.09	4.44E-05	9.05E-04
SRP-dependent cotranslational protein targeting to membrane (R-HSA-1799339)	110	26	2.17	+	12	2.69E-21	5.09E-19
Selenoamino acid metabolism (R-HSA-2408522)	107	25	2.11	+	11.87	2.22E-20	2.74E-18
Nonsense Mediated Decay (NMD) enhanced by the Exon Junction Complex (EJC) (R-HSA-975957)	112	24	2.21	+	10.88	1.18E-18	1.26E-16
Nonsense-Mediated Decay (NMD) (R-HSA-927802)	112	24	2.21	+	10.88	1.18E-18	1.21E-16
Regulation of expression of SLITs and ROBOs (R-HSA-9010553)	152	32	2.99	+	10.69	2.39E-24	9.84E-22
Cellular response to starvation (R-HSA-9711097)	135	27	2.66	+	10.16	4.90E-20	5.75E-18
Influenza Viral RNA Transcription and Replication (R-HSA-168273)	130	26	2.56	+	10.16	2.51E-19	2.81E-17
p53-Independent G1/S DNA damage checkpoint (R-HSA-69613)	47	9	0.93	+	9.73	2.67E-07	1.29E-05
p53-Independent DNA Damage Response (R-HSA-69610)	47	9	0.93	+	9.73	2.67E-07	1.27E-05
Ubiquitin Mediated Degradation of Phosphorylated Cdc25A (R-HSA-69601)	47	9	0.93	+	9.73	2.67E-07	1.24E-05
AUF1 (hnRNP D0) binds and destabilizes mRNA (R-HSA-450408)	48	9	0.95	+	9.52	3.23E-07	1.48E-05
Cross-presentation of soluble exogenous antigens (endosomes) (R-HSA-1236978)	44	8	0.87	+	9.23	1.91E-06	7.24E-05
Somitogenesis (R-HSA-9824272)	44	8	0.87	+	9.23	1.91E-06	7.13E-05
Stabilization of p53 (R-HSA-69541)	50	9	0.98	+	9.14	4.67E-07	2.02E-05
Regulation of ornithine decarboxylase (ODC) (R-HSA-350562)	45	8	0.89	+	9.03	2.28E-06	8.27E-05
Regulation of activated PAK-2p34 by proteasome mediated degradation (R-HSA-211733)	45	8	0.89	+	9.03	2.28E-06	8.15E-05
ER-Phagosome pathway (R-HSA-1236974)	79	14	1.56	+	9	3.45E-10	1.98E-08
Influenza Infection (R-HSA-168255)	148	26	2.91	+	8.92	7.62E-18	6.96E-16
Signaling by ROBO receptors (R-HSA-376176)	189	33	3.72	+	8.87	2.36E-22	5.82E-20
Autodegradation of the E3 ubiquitin ligase COP1 (R-HSA-349425)	46	8	0.91	+	8.83	2.71E-06	9.56E-05
Vif-mediated degradation of APOBEC3G (R-HSA-180585)	47	8	0.93	+	8.64	3.22E-06	1.10E-04
Vpu mediated degradation of CD4 (R-HSA-180534)	47	8	0.93	+	8.64	3.22E-06	1.09E-04
Ubiquitin-dependent degradation of Cyclin D (R-HSA-75815)	47	8	0.93	+	8.64	3.22E-06	1.07E-04
Regulation of Apoptosis (R-HSA-169911)	48	8	0.95	+	8.46	3.80E-06	1.23E-04
Degradation of AXIN (R-HSA-4641257)	48	8	0.95	+	8.46	3.80E-06	1.22E-04
Negative regulation of NOTCH4 signaling (R-HSA-9604323)	49	8	0.96	+	8.29	4.46E-06	1.41E-04
GSK3B and BTRC:CUL1-mediated-degradation of NFE2L2 (R-HSA-9762114)	49	8	0.96	+	8.29	4.46E-06	1.39E-04
FBXL7 down-regulates AURKA during mitotic entry and in early mitosis (R-HSA-8854050)	50	8	0.98	+	8.13	5.22E-06	1.59E-04

Regulation of RUNX3 expression and activity (R-HSA-8941858)	50	8	0.98	+	8.13	5.22E-06	1.57E-04
Hh mutants are degraded by ERAD (R-HSA-5362768)	50	8	0.98	+	8.13	5.22E-06	1.55E-04
SCF-beta-TrCP mediated degradation of Emi1 (R-HSA-174113)	50	8	0.98	+	8.13	5.22E-06	1.53E-04
Metabolism of polyamines (R-HSA-351202)	50	8	0.98	+	8.13	5.22E-06	1.51E-04
Degradation of DVL (R-HSA-4641258)	50	8	0.98	+	8.13	5.22E-06	1.50E-04
p53-Dependent G1/S DNA damage checkpoint (R-HSA-69580)	57	9	1.12	+	8.02	1.49E-06	6.02E-05
p53-Dependent G1 DNA Damage Response (R-HSA-69563)	57	9	1.12	+	8.02	1.49E-06	5.92E-05
Asymmetric localization of PCP proteins (R-HSA-4608870)	51	8	1	+	7.97	6.09E-06	1.67E-04
SARS-CoV-1-host interactions (R-HSA-9692914)	84	13	1.65	+	7.86	8.46E-09	4.54E-07
Degradation of GLI2 by the proteasome (R-HSA-5610783)	52	8	1.02	+	7.81	7.07E-06	1.92E-04
Degradation of GLI1 by the proteasome (R-HSA-5610780)	52	8	1.02	+	7.81	7.07E-06	1.90E-04
Hh mutants abrogate ligand secretion (R-HSA-5387390)	52	8	1.02	+	7.81	7.07E-06	1.88E-04
G1/S DNA Damage Checkpoints (R-HSA-69615)	59	9	1.16	+	7.75	2.01E-06	7.39E-05
GLI3 is processed to GLI3R by the proteasome (R-HSA-5610785)	53	8	1.04	+	7.67	8.19E-06	2.15E-04
NIK-->noncanonical NF-kB signaling (R-HSA-5676590)	53	8	1.04	+	7.67	8.19E-06	2.13E-04
Antigen processing-Cross presentation (R-HSA-1236975)	94	14	1.85	+	7.56	3.73E-09	2.09E-07
Hedgehog ligand biogenesis (R-HSA-5358346)	54	8	1.06	+	7.52	9.46E-06	2.43E-04
Formation of paraxial mesoderm (R-HSA-9793380)	54	8	1.06	+	7.52	9.46E-06	2.40E-04
Dectin-1 mediated noncanonical NF-kB signaling (R-HSA-5607761)	54	8	1.06	+	7.52	9.46E-06	2.38E-04
Major pathway of rRNA processing in the nucleolus and cytosol (R-HSA-6791226)	169	25	3.33	+	7.51	2.31E-15	1.67E-13
Respiratory electron transport, ATP synthesis by chemiosmotic coupling, and heat production by uncoupling proteins. (R-HSA-163200)	96	14	1.89	+	7.41	4.94E-09	2.71E-07
SCF(Skp2)-mediated degradation of p27/p21 (R-HSA-187577)	55	8	1.08	+	7.39	1.09E-05	2.71E-04
Defective CFTR causes cystic fibrosis (R-HSA-5678895)	55	8	1.08	+	7.39	1.09E-05	2.68E-04
rRNA processing in the nucleus and cytosol (R-HSA-8868773)	179	26	3.52	+	7.38	9.68E-16	7.96E-14
PCP/CE pathway (R-HSA-4086400)	70	10	1.38	+	7.26	1.00E-06	4.19E-05
rRNA processing (R-HSA-72312)	185	26	3.64	+	7.14	2.20E-15	1.64E-13
Translation (R-HSA-72766)	271	38	5.34	+	7.12	3.99E-22	8.95E-20
Regulation of RAS by GAPs (R-HSA-5658442)	58	8	1.14	+	7.01	1.63E-05	3.90E-04
Oxygen-dependent proline hydroxylation of Hypoxia-inducible Factor Alpha (R-HSA-1234176)	59	8	1.16	+	6.89	1.85E-05	4.35E-04
Regulation of RUNX2 expression and activity (R-HSA-8939902)	59	8	1.16	+	6.89	1.85E-05	4.31E-04

Hedgehog 'on' state (R-HSA-5632684)	67	9	1.32	+	6.82	5.97E-06	1.65E-04
Regulation of PTEN stability and activity (R-HSA-8948751)	61	8	1.2	+	6.66	2.38E-05	5.43E-04
Activation of NF-kappaB in B cells (R-HSA-1169091)	61	8	1.2	+	6.66	2.38E-05	5.38E-04
Autodegradation of Cdh1 by Cdh1:APC/C (R-HSA-174084)	61	8	1.2	+	6.66	2.38E-05	5.33E-04
ABC transporter disorders (R-HSA-5619084)	62	8	1.22	+	6.55	2.69E-05	5.91E-04
Regulation of mRNA stability by proteins that bind AU-rich elements (R-HSA-450531)	78	10	1.54	+	6.51	2.77E-06	9.63E-05
APC/C:Cdc20 mediated degradation of Securin (R-HSA-174154)	63	8	1.24	+	6.45	3.02E-05	6.60E-04
Orc1 removal from chromatin (R-HSA-68949)	64	8	1.26	+	6.35	3.40E-05	7.29E-04
Metabolism of amino acids and derivatives (R-HSA-71291)	274	34	5.4	+	6.3	3.88E-18	3.83E-16
Cellular response to hypoxia (R-HSA-1234174)	65	8	1.28	+	6.25	3.81E-05	8.10E-04
The role of GTSE1 in G2/M progression after G2 checkpoint (R-HSA-8852276)	66	8	1.3	+	6.16	4.26E-05	8.91E-04
RUNX1 regulates transcription of genes involved in differentiation of HSCs (R-HSA-8939236)	66	8	1.3	+	6.16	4.26E-05	8.84E-04
CDK-mediated phosphorylation and removal of Cdc6 (R-HSA-69017)	67	8	1.32	+	6.06	4.76E-05	9.55E-04
Cdc20:Phospho-APC/C mediated degradation of Cyclin A (R-HSA-174184)	67	8	1.32	+	6.06	4.76E-05	9.47E-04
MAPK6/MAPK4 signaling (R-HSA-5687128)	76	9	1.5	+	6.01	1.71E-05	4.04E-04
the cell cycle checkpoint (R-HSA-179419)	68	8	1.34	+	5.98	5.31E-05	1.04E-03
FCERI mediated NF-kB activation (R-HSA-2871837)	77	9	1.52	+	5.94	1.90E-05	4.38E-04
TNFR2 non-canonical NF-kB pathway (R-HSA-5668541)	69	8	1.36	+	5.89	5.90E-05	1.13E-03
proteins in late mitosis/early G1 (R-HSA-174178)	69	8	1.36	+	5.89	5.90E-05	1.12E-03
SARS-CoV-1 Infection (R-HSA-9678108)	122	14	2.4	+	5.83	1.13E-07	5.66E-06
APC/C:Cdc20 mediated degradation of mitotic proteins (R-HSA-176409)	70	8	1.38	+	5.8	6.55E-05	1.22E-03
Activation of APC/C and APC/C:Cdc20 mediated degradation of mitotic proteins (R-HSA-176814)	71	8	1.4	+	5.72	7.26E-05	1.34E-03
Nuclear events mediated by NFE2L2 (R-HSA-9759194)	80	9	1.58	+	5.71	2.59E-05	5.76E-04
Signaling by NOTCH4 (R-HSA-9013694)	72	8	1.42	+	5.64	8.03E-05	1.45E-03
Downstream signaling events of B Cell Receptor (BCR) (R-HSA-1168372)	73	8	1.44	+	5.57	8.87E-05	1.57E-03
Degradation of beta-catenin by the destruction complex (R-HSA-195253)	74	8	1.46	+	5.49	9.78E-05	1.70E-03
Gastrulation (R-HSA-9758941)	74	8	1.46	+	5.49	9.78E-05	1.69E-03
Cyclin E associated events during G1/S transition (R-HSA-69202)	75	8	1.48	+	5.42	1.08E-04	1.84E-03
Regulation of APC/C activators between G1/S and early anaphase (R-HSA-176408)	75	8	1.48	+	5.42	1.08E-04	1.83E-03

Respiratory electron transport (R-HSA-611105)	85	9	1.67	+	5.38	4.23E-05	8.92E-04
Complex I biogenesis (R-HSA-6799198)	48	5	0.95	+	5.29	2.43E-03	3.22E-02
Cyclin A:Cdk2-associated events at S phase entry (R-HSA-69656)	77	8	1.52	+	5.28	1.30E-04	2.16E-03
Downstream TCR signaling (R-HSA-202424)	87	9	1.71	+	5.25	5.09E-05	1.00E-03
Apoptosis (R-HSA-109581)	136	14	2.68	+	5.23	4.39E-07	1.93E-05
UCH proteinases (R-HSA-5689603)	78	8	1.54	+	5.21	1.42E-04	2.34E-03
The citric acid (TCA) cycle and respiratory electron transport (R-HSA-1428517)	137	14	2.7	+	5.19	4.80E-07	2.04E-05
mRNA Splicing - Minor Pathway (R-HSA-72165)	49	5	0.96	+	5.18	2.66E-03	3.47E-02
Hedgehog 'off' state (R-HSA-5610787)	90	9	1.77	+	5.08	6.67E-05	1.24E-03
Beta-catenin independent WNT signaling (R-HSA-3858494)	112	11	2.21	+	4.99	1.23E-05	2.96E-04
Transcriptional regulation by RUNX2 (R-HSA-8878166)	92	9	1.81	+	4.97	7.93E-05	1.44E-03
G2/M Checkpoints (R-HSA-69481)	123	12	2.42	+	4.95	5.25E-06	1.49E-04
Regulation of mitotic cell cycle (R-HSA-453276)	82	8	1.61	+	4.95	2.02E-04	3.26E-03
APC/C-mediated degradation of cell cycle proteins (R-HSA-174143)	82	8	1.61	+	4.95	2.02E-04	3.24E-03
PTEN Regulation (R-HSA-6807070)	123	12	2.42	+	4.95	5.25E-06	1.47E-04
Assembly of the pre-replicative complex (R-HSA-68867)	83	8	1.63	+	4.9	2.20E-04	3.50E-03
SARS-CoV-2-host interactions (R-HSA-9705683)	166	16	3.27	+	4.9	1.62E-07	7.97E-06
ABC-family proteins mediated transport (R-HSA-382556)	83	8	1.63	+	4.9	2.20E-04	3.48E-03
KEAP1-NFE2L2 pathway (R-HSA-9755511)	106	10	2.09	+	4.79	4.35E-05	8.94E-04
Switching of origins to a post-replicative state (R-HSA-69052)	85	8	1.67	+	4.78	2.60E-04	4.05E-03
Transcriptional regulation by RUNX3 (R-HSA-8878159)	85	8	1.67	+	4.78	2.60E-04	4.03E-03
Mitochondrial biogenesis (R-HSA-1592230)	75	7	1.48	+	4.74	6.63E-04	9.51E-03
Axon guidance (R-HSA-422475)	401	37	7.9	+	4.69	2.14E-15	1.65E-13
CLEC7A (Dectin-1) signaling (R-HSA-5607764)	87	8	1.71	+	4.67	3.05E-04	4.67E-03
Nervous system development (R-HSA-9675108)	417	38	8.21	+	4.63	1.28E-15	1.01E-13
DNA Replication Pre-Initiation (R-HSA-69002)	99	9	1.95	+	4.62	1.40E-04	2.32E-03
Interleukin-1 signaling (R-HSA-9020702)	100	9	1.97	+	4.57	1.52E-04	2.47E-03
Signaling by Hedgehog (R-HSA-5358351)	115	10	2.26	+	4.42	8.73E-05	1.56E-03
Programmed Cell Death (R-HSA-5357801)	161	14	3.17	+	4.42	3.38E-06	1.11E-04
Cellular responses to stress (R-HSA-2262752)	623	54	12.27	+	4.4	3.53E-21	6.21E-19
Host Interactions of HIV factors (R-HSA-162909)	116	10	2.28	+	4.38	9.39E-05	1.64E-03
Cellular response to chemical stress (R-HSA-9711123)	175	15	3.45	+	4.35	1.79E-06	6.91E-05

Cellular responses to stimuli (R-HSA-8953897)	631	54	12.42	+	4.35	6.41E-21	9.88E-19
mRNA Splicing - Major Pathway (R-HSA-72163)	190	16	3.74	+	4.28	1.02E-06	4.19E-05
TCR signaling (R-HSA-202403)	107	9	2.11	+	4.27	2.54E-04	3.98E-03
Metabolism of RNA (R-HSA-8953854)	646	54	12.72	+	4.25	1.91E-20	2.48E-18
TP53 Regulates Metabolic Genes (R-HSA-5628897)	73	6	1.44	+	4.17	3.09E-03	3.98E-02
mRNA Splicing (R-HSA-72172)	198	16	3.9	+	4.1	1.76E-06	6.90E-05
Signaling by the B Cell Receptor (BCR) (R-HSA-983705)	99	8	1.95	+	4.1	7.30E-04	1.02E-02
Synthesis of DNA (R-HSA-69239)	112	9	2.21	+	4.08	3.57E-04	5.40E-03
Ub-specific processing proteases (R-HSA-5689880)	143	11	2.82	+	3.91	1.18E-04	2.00E-03
Fc epsilon receptor (FCERI) signaling (R-HSA-2454202)	117	9	2.3	+	3.91	4.93E-04	7.29E-03
Viral Infection Pathways (R-HSA-9824446)	653	50	12.86	+	3.89	2.77E-17	2.36E-15
C-type lectin receptors (CLRs) (R-HSA-5621481)	119	9	2.34	+	3.84	5.59E-04	8.15E-03
G1/S Transition (R-HSA-69206)	120	9	2.36	+	3.81	5.94E-04	8.61E-03
Transcriptional regulation by RUNX1 (R-HSA-8878171)	149	11	2.93	+	3.75	1.71E-04	2.77E-03
DNA Replication (R-HSA-69306)	122	9	2.4	+	3.75	6.70E-04	9.54E-03
Interleukin-1 family signaling (R-HSA-446652)	123	9	2.42	+	3.72	7.10E-04	9.95E-03
Separation of Sister Chromatids (R-HSA-2467813)	168	12	3.31	+	3.63	1.19E-04	1.99E-03
SARS-CoV-2 Infection (R-HSA-9694516)	240	17	4.73	+	3.6	5.06E-06	1.56E-04
Infectious disease (R-HSA-5663205)	798	56	15.71	+	3.56	1.17E-17	1.03E-15
HIV Infection (R-HSA-162906)	202	14	3.98	+	3.52	4.49E-05	9.07E-04
Signaling by NOTCH (R-HSA-157118)	147	10	2.89	+	3.45	6.45E-04	9.30E-03
Mitotic G1 phase and G1/S transition (R-HSA-453279)	134	9	2.64	+	3.41	1.31E-03	1.80E-02
S Phase (R-HSA-69242)	150	10	2.95	+	3.39	7.54E-04	1.05E-02
TCF dependent signaling in response to WNT (R-HSA-201681)	124	8	2.44	+	3.28	3.09E-03	3.97E-02
Disorders of transmembrane transporters (R-HSA-5619115)	124	8	2.44	+	3.28	3.09E-03	3.95E-02
Cell Cycle Checkpoints (R-HSA-69620)	233	15	4.59	+	3.27	5.62E-05	1.08E-03
RAF/MAP kinase cascade (R-HSA-5673001)	189	12	3.72	+	3.22	3.56E-04	5.42E-03
Developmental Biology (R-HSA-1266738)	682	43	13.43	+	3.2	5.78E-12	3.56E-10
MAPK1/MAPK3 signaling (R-HSA-5684996)	192	12	3.78	+	3.17	4.11E-04	6.14E-03
Processing of Capped Intron-Containing Pre-mRNA (R-HSA-72203)	260	16	5.12	+	3.13	5.46E-05	1.06E-03
PIP3 activates AKT signaling (R-HSA-1257604)	198	12	3.9	+	3.08	5.42E-04	7.95E-03
Mitotic Anaphase (R-HSA-68882)	203	12	4	+	3	6.77E-04	9.59E-03

Mitotic Metaphase and Anaphase (R-HSA-2555396)	204	12	4.02	+	2.99	7.07E-04	9.96E-03
SARS-CoV Infections (R-HSA-9679506)	340	20	6.69	+	2.99	1.22E-05	2.98E-04
MAPK family signaling cascades (R-HSA-5683057)	223	13	4.39	+	2.96	4.64E-04	6.90E-03
Signaling by WNT (R-HSA-195721)	196	11	3.86	+	2.85	1.71E-03	2.32E-02
Metabolism of proteins (R-HSA-392499)	1448	78	28.51	+	2.74	4.49E-18	4.26E-16
Deubiquitination (R-HSA-5688426)	205	11	4.04	+	2.73	2.44E-03	3.21E-02
Neutrophil degranulation (R-HSA-6798695)	394	21	7.76	+	2.71	3.23E-05	6.98E-04
Class I MHC mediated antigen processing & presentation (R-HSA-983169)	301	16	5.93	+	2.7	2.98E-04	4.60E-03
Intracellular signaling by second messengers (R-HSA-9006925)	230	12	4.53	+	2.65	1.98E-03	2.66E-02
Signaling by Interleukins (R-HSA-449147)	339	17	6.67	+	2.55	3.82E-04	5.75E-03
Disease (R-HSA-1643685)	1328	64	26.15	+	2.45	3.24E-12	2.05E-10
Innate Immune System (R-HSA-168249)	782	32	15.4	+	2.08	7.37E-05	1.35E-03
Metabolism (R-HSA-1430728)	1446	59	28.47	+	2.07	2.02E-08	1.06E-06
Adaptive Immune System (R-HSA-1280218)	587	23	11.56	+	1.99	1.80E-03	2.42E-02
Cytokine Signaling in Immune system (R-HSA-1280215)	564	22	11.11	+	1.98	2.51E-03	3.30E-02
Immune System (R-HSA-168256)	1460	50	28.75	+	1.74	6.46E-05	1.22E-03
RNA Polymerase II Transcription (R-HSA-73857)	954	32	18.78	+	1.7	3.04E-03	3.95E-02
Post-translational protein modification (R-HSA-597592)	986	33	19.41	+	1.7	2.39E-03	3.19E-02
Unclassified (UNCLASSIFIED)	3986	41	78.48	-	0.52	2.21E-08	1.14E-06

Analysis Type:	PANTHER Overrepresentation Test (Released 20240807)						
Annotation Version and Release Date:	Reactome version 86 Released 2023-09-07						
Analyzed List:	DEG week 2 (Homo sapiens)						
Reference List:	Background gene list.txt (Homo sapiens)						
Test Type:	FISHER						
Correction:	FDR		DEG week 2	DEG week 2	DEG week 2	DEG week 2	DEG week 2
Reactome pathways	Background gene list.txt	DEG week 2 (379)	(expected)	(over/ under)	(fold Enrichment)	(raw P-value)	(FDR)
SARS-CoV-1 modulates host translation machinery (R-HSA-9735869)	35	25	1.23	+	20.29	2.81E-29	1.87E-27
Peptide chain elongation (R-HSA-156902)	87	58	3.06	+	18.94	2.50E-64	1.54E-61
Eukaryotic Translation Elongation (R-HSA-156842)	90	59	3.17	+	18.62	9.08E-65	1.12E-61
Viral mRNA Translation (R-HSA-192823)	87	57	3.06	+	18.61	1.55E-62	4.79E-60
Eukaryotic Translation Termination (R-HSA-72764)	90	58	3.17	+	18.31	5.41E-63	2.22E-60
Selenocysteine synthesis (R-HSA-2408557)	90	57	3.17	+	17.99	3.06E-61	5.80E-59
(EJC) (R-HSA-975956)	92	58	3.24	+	17.91	3.80E-62	9.37E-60
SARS-CoV-2 modulates host translation machinery (R-HSA-9754678)	48	30	1.69	+	17.76	3.28E-32	2.31E-30
Formation of a pool of free 40S subunits (R-HSA-72689)	99	61	3.48	+	17.5	1.78E-64	1.46E-61
Response of EIF2AK4 (GCN2) to amino acid deficiency (R-HSA-9633012)	94	57	3.31	+	17.23	1.25E-59	2.06E-57
Modulation by Mtb of host immune system (R-HSA-9637628)	5	3	0.18	+	17.05	4.10E-04	4.91E-03
L13a-mediated translational silencing of Ceruloplasmin expression (R-HSA-156827)	108	62	3.8	+	16.31	9.10E-63	3.21E-60
GTP hydrolysis and joining of the 60S ribosomal subunit (R-HSA-72706)	109	62	3.84	+	16.16	2.05E-62	5.62E-60
Formation of the ternary complex, and subsequently, the 43S complex (R-HSA-72695)	50	28	1.76	+	15.91	3.28E-28	2.08E-26
Formation of ATP by chemiosmotic coupling (R-HSA-163210)	9	5	0.32	+	15.78	5.90E-06	8.04E-05
SRP-dependent cotranslational protein targeting to membrane (R-HSA-1799339)	110	61	3.87	+	15.75	1.88E-60	3.31E-58
Selenoamino acid metabolism (R-HSA-2408522)	107	59	3.77	+	15.66	2.98E-58	4.33E-56
Cap-dependent Translation Initiation (R-HSA-72737)	116	63	4.08	+	15.43	1.15E-61	2.58E-59
Eukaryotic Translation Initiation (R-HSA-72613)	116	63	4.08	+	15.43	1.15E-61	2.37E-59

Nonsense Mediated Decay (NMD) enhanced by the Exon Junction Complex (EJC) (R-HSA-975957)	112	59	3.94	+	14.97	1.20E-56	1.65E-54
Nonsense-Mediated Decay (NMD) (R-HSA-927802)	112	59	3.94	+	14.97	1.20E-56	1.56E-54
Translation initiation complex formation (R-HSA-72649)	56	29	1.97	+	14.71	7.61E-28	4.69E-26
subsequent binding to 43S (R-HSA-72662)	57	29	2.01	+	14.45	1.50E-27	9.03E-26
SLBP independent Processing of Histone Pre-mRNAs (R-HSA-111367)	8	4	0.28	+	14.2	9.45E-05	1.21E-03
Ribosomal scanning and start codon recognition (R-HSA-72702)	56	28	1.97	+	14.2	2.33E-26	1.34E-24
Regulation of expression of SLITs and ROBOs (R-HSA-9010553)	152	76	5.35	+	14.2	5.92E-71	1.46E-67
Cellular response to starvation (R-HSA-9711097)	135	61	4.75	+	12.84	2.78E-53	3.42E-51
Influenza Viral RNA Transcription and Replication (R-HSA-168273)	130	58	4.58	+	12.67	3.04E-50	3.57E-48
SLBP Dependent Processing of Replication-Dependent Histone Pre-mRNAs (R-HSA-77588)	9	4	0.32	+	12.63	1.65E-04	2.05E-03
Signaling by ROBO receptors (R-HSA-376176)	189	77	6.65	+	11.57	2.92E-63	1.44E-60
AUF1 (hnRNP D0) binds and destabilizes mRNA (R-HSA-450408)	48	19	1.69	+	11.25	7.03E-16	3.33E-14
SARS-CoV-1-host interactions (R-HSA-9692914)	84	33	2.96	+	11.16	1.32E-26	7.74E-25
Influenza Infection (R-HSA-168255)	148	58	5.21	+	11.13	2.88E-46	2.63E-44
Vif-mediated degradation of APOBEC3G (R-HSA-180585)	47	18	1.65	+	10.88	8.29E-15	3.86E-13
Regulation of activated PAK-2p34 by proteasome mediated degradation (R-HSA-211733)	45	17	1.58	+	10.73	6.14E-14	2.66E-12
Autodegradation of the E3 ubiquitin ligase COP1 (R-HSA-349425)	46	17	1.62	+	10.5	9.43E-14	4.01E-12
Major pathway of rRNA processing in the nucleolus and cytosol (R-HSA-6791226)	169	62	5.95	+	10.42	2.14E-47	2.20E-45
Somitogenesis (R-HSA-9824272)	44	16	1.55	+	10.33	6.88E-13	2.12E-11
Vpu mediated degradation of CD4 (R-HSA-180534)	47	17	1.65	+	10.28	1.43E-13	5.98E-12
Ubiquitin-dependent degradation of Cyclin D (R-HSA-75815)	47	17	1.65	+	10.28	1.43E-13	5.88E-12
p53-Independent G1/S DNA damage checkpoint (R-HSA-69613)	47	17	1.65	+	10.28	1.43E-13	5.78E-12
p53-Independent DNA Damage Response (R-HSA-69610)	47	17	1.65	+	10.28	1.43E-13	5.69E-12
Ubiquitin Mediated Degradation of Phosphorylated Cdc25A (R-HSA-69601)	47	17	1.65	+	10.28	1.43E-13	5.60E-12
rRNA processing in the nucleus and cytosol (R-HSA-8868773)	179	64	6.3	+	10.16	4.09E-48	4.38E-46
Regulation of ornithine decarboxylase (ODC) (R-HSA-350562)	45	16	1.58	+	10.1	1.03E-12	2.96E-11
Regulation of Apoptosis (R-HSA-169911)	48	17	1.69	+	10.06	2.14E-13	8.14E-12
Degradation of AXIN (R-HSA-4641257)	48	17	1.69	+	10.06	2.14E-13	8.01E-12
Negative regulation of NOTCH4 signaling (R-HSA-9604323)	49	17	1.72	+	9.86	3.18E-13	1.14E-11
GSK3B and BTRC:CUL1-mediated-degradation of NFE2L2 (R-HSA-9762114)	49	17	1.72	+	9.86	3.18E-13	1.12E-11

rRNA processing (R-HSA-72312)	185	64	6.51	+	9.83	4.56E-47	4.50E-45
Cross-presentation of soluble exogenous antigens (endosomes) (R-HSA-1236978)	44	15	1.55	+	9.68	1.09E-11	2.48E-10
FBXL7 down-regulates AURKA during mitotic entry and in early mitosis (R-HSA-8854050)	50	17	1.76	+	9.66	4.66E-13	1.62E-11
Regulation of RUNX3 expression and activity (R-HSA-8941858)	50	17	1.76	+	9.66	4.66E-13	1.60E-11
Hh mutants are degraded by ERAD (R-HSA-5362768)	50	17	1.76	+	9.66	4.66E-13	1.58E-11
SCF-beta-TrCP mediated degradation of Emi1 (R-HSA-174113)	50	17	1.76	+	9.66	4.66E-13	1.55E-11
Stabilization of p53 (R-HSA-69541)	50	17	1.76	+	9.66	4.66E-13	1.53E-11
Metabolism of polyamines (R-HSA-351202)	50	17	1.76	+	9.66	4.66E-13	1.51E-11
Degradation of DVL (R-HSA-4641258)	50	17	1.76	+	9.66	4.66E-13	1.49E-11
Folding of actin by CCT/TriC (R-HSA-390450)	9	3	0.32	+	9.47	3.10E-03	3.48E-02
Integration of provirus (R-HSA-162592)	9	3	0.32	+	9.47	3.10E-03	3.46E-02
Asymmetric localization of PCP proteins (R-HSA-4608870)	51	17	1.8	+	9.47	6.77E-13	2.11E-11
Degradation of GLI2 by the proteasome (R-HSA-5610783)	52	17	1.83	+	9.29	9.75E-13	2.90E-11
Degradation of GLI1 by the proteasome (R-HSA-5610780)	52	17	1.83	+	9.29	9.75E-13	2.86E-11
Hh mutants abrogate ligand secretion (R-HSA-5387390)	52	17	1.83	+	9.29	9.75E-13	2.83E-11
GLI3 is processed to GLI3R by the proteasome (R-HSA-5610785)	53	17	1.87	+	9.11	1.39E-12	3.94E-11
NIK-->noncanonical NF-kB signaling (R-HSA-5676590)	53	17	1.87	+	9.11	1.39E-12	3.89E-11
Hedgehog ligand biogenesis (R-HSA-5358346)	54	17	1.9	+	8.94	1.96E-12	5.26E-11
Dectin-1 mediated noncanonical NF-kB signaling (R-HSA-5607761)	54	17	1.9	+	8.94	1.96E-12	5.21E-11
SCF(Skp2)-mediated degradation of p27/p21 (R-HSA-187577)	55	17	1.94	+	8.78	2.75E-12	6.99E-11
Defective CFTR causes cystic fibrosis (R-HSA-5678895)	55	17	1.94	+	8.78	2.75E-12	6.92E-11
Protein methylation (R-HSA-8876725)	13	4	0.46	+	8.74	8.39E-04	9.90E-03
Oxygen-dependent proline hydroxylation of Hypoxia-inducible Factor Alpha (R-HSA-1234176)	59	18	2.08	+	8.67	7.97E-13	2.43E-11
Regulation of RUNX2 expression and activity (R-HSA-8939902)	59	18	2.08	+	8.67	7.97E-13	2.40E-11
ER-Phagosome pathway (R-HSA-1236974)	79	24	2.78	+	8.63	1.23E-16	6.19E-15
APC/C:Cdc20 mediated degradation of Securin (R-HSA-174154)	63	19	2.22	+	8.57	2.30E-13	8.46E-12
p53-Dependent G1/S DNA damage checkpoint (R-HSA-69580)	57	17	2.01	+	8.47	5.28E-12	1.29E-10
p53-Dependent G1 DNA Damage Response (R-HSA-69563)	57	17	2.01	+	8.47	5.28E-12	1.28E-10
Formation of paraxial mesoderm (R-HSA-9793380)	54	16	1.9	+	8.42	2.52E-11	5.45E-10
Autodegradation of Cdh1 by Cdh1:APC/C (R-HSA-174084)	61	18	2.15	+	8.38	1.51E-12	4.15E-11

Regulation of RAS by GAPs (R-HSA-5658442)	58	17	2.04	+	8.33	7.23E-12	1.73E-10
G1/S DNA Damage Checkpoints (R-HSA-69615)	59	17	2.08	+	8.19	9.83E-12	2.27E-10
RUNX1 regulates transcription of genes involved in differentiation of HSCs (R-HSA-8939236)	66	19	2.32	+	8.18	5.89E-13	1.86E-11
SARS-CoV-1 Infection (R-HSA-9678108)	122	35	4.29	+	8.15	8.06E-23	4.52E-21
Cristae formation (R-HSA-8949613)	21	6	0.74	+	8.12	6.33E-05	8.26E-04
Regulation of mRNA stability by proteins that bind AU-rich elements (R-HSA-450531)	78	22	2.75	+	8.01	1.35E-14	6.03E-13
Metabolism of amino acids and derivatives (R-HSA-71291)	274	77	9.64	+	7.98	3.63E-49	4.06E-47
Regulation of PTEN stability and activity (R-HSA-8948751)	61	17	2.15	+	7.92	1.78E-11	4.00E-10
Activation of NF-kappaB in B cells (R-HSA-1169091)	61	17	2.15	+	7.92	1.78E-11	3.96E-10
Cellular response to hypoxia (R-HSA-1234174)	65	18	2.29	+	7.87	5.05E-12	1.25E-10
proteins in late mitosis/early G1 (R-HSA-174178)	69	19	2.43	+	7.82	1.43E-12	3.96E-11
ABC transporter disorders (R-HSA-5619084)	62	17	2.18	+	7.79	2.38E-11	5.19E-10
Translation (R-HSA-72766)	271	74	9.54	+	7.76	3.23E-46	2.85E-44
APC/C:Cdc20 mediated degradation of mitotic proteins (R-HSA-176409)	70	19	2.46	+	7.71	1.90E-12	5.14E-11
CDK-mediated phosphorylation and removal of Cdc6 (R-HSA-69017)	67	18	2.36	+	7.63	8.90E-12	2.09E-10
Cdc20:Phospho-APC/C mediated degradation of Cyclin A (R-HSA-174184)	67	18	2.36	+	7.63	8.90E-12	2.07E-10
Activation of APC/C and APC/C:Cdc20 mediated degradation of mitotic proteins (R-HSA-176814)	71	19	2.5	+	7.6	2.51E-12	6.51E-11
Orc1 removal from chromatin (R-HSA-68949)	64	17	2.25	+	7.55	4.16E-11	8.69E-10
cell cycle checkpoint (R-HSA-179419)	68	18	2.39	+	7.52	1.17E-11	2.65E-10
Chaperone Mediated Autophagy (R-HSA-9613829)	19	5	0.67	+	7.48	4.07E-04	4.89E-03
Downstream signaling events of B Cell Receptor (BCR) (R-HSA-1168372)	73	19	2.57	+	7.39	4.32E-12	1.08E-10
The role of GTSE1 in G2/M progression after G2 checkpoint (R-HSA-8852276)	66	17	2.32	+	7.32	7.11E-11	1.44E-09
PCP/CE pathway (R-HSA-4086400)	70	18	2.46	+	7.31	2.00E-11	4.41E-10
Antigen processing-Cross presentation (R-HSA-1236975)	94	24	3.31	+	7.25	9.76E-15	4.46E-13
Hedgehog 'on' state (R-HSA-5632684)	67	17	2.36	+	7.21	9.22E-11	1.82E-09
Nuclear events mediated by NFE2L2 (R-HSA-9759194)	80	20	2.82	+	7.1	2.65E-12	6.80E-11
Signaling by NOTCH4 (R-HSA-9013694)	72	18	2.53	+	7.1	3.35E-11	7.18E-10
Infection with Mycobacterium tuberculosis (R-HSA-9635486)	24	6	0.84	+	7.1	1.44E-04	1.80E-03
TNFR2 non-canonical NF-kB pathway (R-HSA-5668541)	69	17	2.43	+	7	1.53E-10	2.92E-09
Cyclin E associated events during G1/S transition (R-HSA-69202)	75	18	2.64	+	6.82	7.02E-11	1.44E-09

Regulation of APC/C activators between G1/S and early anaphase (R-HSA-176408)	75	18	2.64	+	6.82	7.02E-11	1.43E-09
heat production by uncoupling proteins. (R-HSA-163200)	96	23	3.38	+	6.81	1.56E-13	6.03E-12
MAPK6/MAPK4 signaling (R-HSA-5687128)	76	18	2.68	+	6.73	8.90E-11	1.77E-09
RHO GTPases Activate ROCKs (R-HSA-5627117)	17	4	0.6	+	6.68	2.50E-03	2.88E-02
Cyclin A:Cdk2-associated events at S phase entry (R-HSA-69656)	77	18	2.71	+	6.64	1.13E-10	2.20E-09
FCERI mediated NF-kB activation (R-HSA-2871837)	77	18	2.71	+	6.64	1.13E-10	2.18E-09
Regulation of mitotic cell cycle (R-HSA-453276)	82	19	2.89	+	6.58	4.00E-11	8.50E-10
APC/C-mediated degradation of cell cycle proteins (R-HSA-174143)	82	19	2.89	+	6.58	4.00E-11	8.43E-10
UCH proteinases (R-HSA-5689603)	78	18	2.75	+	6.56	1.42E-10	2.73E-09
Degradation of beta-catenin by the destruction complex (R-HSA-195253)	74	17	2.6	+	6.53	5.01E-10	9.22E-09
Assembly of the pre-replicative complex (R-HSA-68867)	83	19	2.92	+	6.5	5.02E-11	1.04E-09
Response of Mtb to phagocytosis (R-HSA-9637690)	22	5	0.77	+	6.46	8.44E-04	9.92E-03
mRNA Splicing - Minor Pathway (R-HSA-72165)	49	11	1.72	+	6.38	7.77E-07	1.11E-05
SARS-CoV-2-host interactions (R-HSA-9705683)	166	37	5.84	+	6.33	6.38E-20	3.42E-18
Axon guidance (R-HSA-422475)	401	88	14.12	+	6.23	1.09E-46	1.04E-44
Gastrulation (R-HSA-9758941)	74	16	2.6	+	6.14	4.22E-09	7.13E-08
Respiratory electron transport (R-HSA-611105)	85	18	2.99	+	6.02	6.41E-10	1.16E-08
Switching of origins to a post-replicative state (R-HSA-69052)	85	18	2.99	+	6.02	6.41E-10	1.15E-08
Transcriptional regulation by RUNX3 (R-HSA-8878159)	85	18	2.99	+	6.02	6.41E-10	1.15E-08
Nervous system development (R-HSA-9675108)	417	88	14.68	+	6	3.35E-45	2.85E-43
CLEC7A (Dectin-1) signaling (R-HSA-5607764)	87	18	3.06	+	5.88	9.58E-10	1.70E-08
Downstream TCR signaling (R-HSA-202424)	87	18	3.06	+	5.88	9.58E-10	1.69E-08
Transcriptional regulation by RUNX2 (R-HSA-8878166)	92	19	3.24	+	5.87	3.36E-10	6.32E-09
SARS-CoV-1 activates/modulates innate immune responses (R-HSA-9692916)	34	7	1.2	+	5.85	1.50E-04	1.86E-03
ABC-family proteins mediated transport (R-HSA-382556)	83	17	2.92	+	5.82	3.33E-09	5.66E-08
DNA Replication Pre-Initiation (R-HSA-69002)	99	20	3.48	+	5.74	1.74E-10	3.29E-09
stimulation (R-HSA-8950505)	35	7	1.23	+	5.68	1.81E-04	2.23E-03
Hedgehog 'off' state (R-HSA-5610787)	90	18	3.17	+	5.68	1.71E-09	2.95E-08
Signaling by the B Cell Receptor (BCR) (R-HSA-983705)	99	19	3.48	+	5.45	1.26E-09	2.20E-08
Interleukin-1 signaling (R-HSA-9020702)	100	19	3.52	+	5.4	1.50E-09	2.61E-08
Host Interactions of HIV factors (R-HSA-162909)	116	22	4.08	+	5.39	7.71E-11	1.55E-09

Metabolism of RNA (R-HSA-8953854)	646	122	22.74	+	5.37	2.13E-58	3.29E-56
KEAP1-NFE2L2 pathway (R-HSA-9755511)	106	20	3.73	+	5.36	6.28E-10	1.15E-08
Complex I biogenesis (R-HSA-6799198)	48	9	1.69	+	5.33	3.75E-05	4.91E-04
mRNA Splicing - Major Pathway (R-HSA-72163)	190	35	6.69	+	5.23	3.97E-16	1.92E-14
Apoptosis (R-HSA-109581)	136	25	4.79	+	5.22	7.86E-12	1.86E-10
mRNA Splicing (R-HSA-72172)	198	36	6.97	+	5.17	2.28E-16	1.12E-14
RNA Polymerase II Transcription Termination (R-HSA-73856)	61	11	2.15	+	5.12	7.63E-06	1.03E-04
Beta-catenin independent WNT signaling (R-HSA-3858494)	112	20	3.94	+	5.07	1.74E-09	2.97E-08
B-WICH complex positively regulates rRNA expression (R-HSA-5250924)	28	5	0.99	+	5.07	2.65E-03	3.04E-02
Detoxification of Reactive Oxygen Species (R-HSA-3299685)	28	5	0.99	+	5.07	2.65E-03	3.03E-02
TCR signaling (R-HSA-202403)	107	19	3.77	+	5.04	4.92E-09	8.25E-08
Smooth Muscle Contraction (R-HSA-445355)	34	6	1.2	+	5.01	1.07E-03	1.25E-02
SARS-CoV-2 Infection (R-HSA-9694516)	240	41	8.45	+	4.85	1.72E-17	8.84E-16
Interleukin-12 signaling (R-HSA-9020591)	41	7	1.44	+	4.85	5.06E-04	6.00E-03
Interleukin-12 family signaling (R-HSA-447115)	47	8	1.65	+	4.84	2.07E-04	2.52E-03
Synthesis of DNA (R-HSA-69239)	112	19	3.94	+	4.82	1.08E-08	1.76E-07
The citric acid (TCA) cycle and respiratory electron transport (R-HSA-1428517)	137	23	4.82	+	4.77	3.69E-10	6.84E-09
Cellular responses to stress (R-HSA-2262752)	623	104	21.93	+	4.74	8.89E-44	7.31E-42
Cellular response to chemical stress (R-HSA-9711123)	175	29	6.16	+	4.71	2.43E-12	6.38E-11
Cellular responses to stimuli (R-HSA-8953897)	631	104	22.21	+	4.68	3.05E-43	2.35E-41
DNA Replication (R-HSA-69306)	122	20	4.29	+	4.66	8.13E-09	1.35E-07
G2/M Checkpoints (R-HSA-69481)	123	20	4.33	+	4.62	9.40E-09	1.55E-07
Fc epsilon receptor (FCERI) signaling (R-HSA-2454202)	117	19	4.12	+	4.61	2.27E-08	3.63E-07
Viral Infection Pathways (R-HSA-9824446)	653	106	22.99	+	4.61	1.71E-43	1.36E-41
E3 ubiquitin ligases ubiquitinate target proteins (R-HSA-8866654)	37	6	1.3	+	4.61	1.69E-03	1.95E-02
TCF dependent signaling in response to WNT (R-HSA-201681)	124	20	4.36	+	4.58	1.08E-08	1.76E-07
rRNA modification in the nucleus and cytosol (R-HSA-6790901)	56	9	1.97	+	4.57	1.32E-04	1.67E-03
C-type lectin receptors (CLRs) (R-HSA-5621481)	119	19	4.19	+	4.54	3.01E-08	4.76E-07
G1/S Transition (R-HSA-69206)	120	19	4.22	+	4.5	3.47E-08	5.44E-07
Signaling by Hedgehog (R-HSA-5358351)	115	18	4.05	+	4.45	9.58E-08	1.43E-06
Programmed Cell Death (R-HSA-5357801)	161	25	5.67	+	4.41	3.50E-10	6.54E-09
PTEN Regulation (R-HSA-6807070)	123	19	4.33	+	4.39	5.23E-08	8.01E-07

Interleukin-1 family signaling (R-HSA-446652)	123	19	4.33	+	4.39	5.23E-08	7.96E-07
Mitotic G1 phase and G1/S transition (R-HSA-453279)	134	20	4.72	+	4.24	4.21E-08	6.52E-07
Iron uptake and transport (R-HSA-917937)	41	6	1.44	+	4.16	2.90E-03	3.26E-02
Infectious disease (R-HSA-5663205)	798	116	28.09	+	4.13	6.38E-43	4.77E-41
Disorders of transmembrane transporters (R-HSA-5619115)	124	18	4.36	+	4.12	3.10E-07	4.49E-06
SARS-CoV Infections (R-HSA-9679506)	340	49	11.97	+	4.09	1.52E-17	7.97E-16
Signaling by NOTCH (R-HSA-157118)	147	21	5.17	+	4.06	4.22E-08	6.50E-07
Processing of Capped Intron-Containing Pre-mRNA (R-HSA-72203)	260	37	9.15	+	4.04	2.70E-13	9.78E-12
Transcriptional regulation by RUNX1 (R-HSA-8878171)	149	21	5.24	+	4	5.36E-08	8.11E-07
S Phase (R-HSA-69242)	150	21	5.28	+	3.98	6.04E-08	9.08E-07
Developmental Biology (R-HSA-1266738)	682	95	24.01	+	3.96	5.23E-33	3.79E-31
TP53 Regulates Metabolic Genes (R-HSA-5628897)	73	10	2.57	+	3.89	2.23E-04	2.70E-03
Separation of Sister Chromatids (R-HSA-2467813)	168	23	5.91	+	3.89	2.16E-08	3.47E-07
Ub-specific processing proteases (R-HSA-5689880)	143	19	5.03	+	3.77	5.95E-07	8.53E-06
mRNA 3'-end processing (R-HSA-72187)	54	7	1.9	+	3.68	2.69E-03	3.06E-02
Bacterial Infection Pathways (R-HSA-9824439)	54	7	1.9	+	3.68	2.69E-03	3.05E-02
HIV Infection (R-HSA-162906)	202	25	7.11	+	3.52	4.09E-08	6.39E-07
RAF/MAP kinase cascade (R-HSA-5673001)	189	23	6.65	+	3.46	1.97E-07	2.91E-06
G2/M Transition (R-HSA-69275)	175	21	6.16	+	3.41	8.52E-07	1.20E-05
Mitochondrial biogenesis (R-HSA-1592230)	75	9	2.64	+	3.41	1.22E-03	1.41E-02
MAPK1/MAPK3 signaling (R-HSA-5684996)	192	23	6.76	+	3.4	2.63E-07	3.83E-06
Mitotic G2-G2/M phases (R-HSA-453274)	176	21	6.2	+	3.39	9.37E-07	1.31E-05
Mitotic Anaphase (R-HSA-68882)	203	24	7.15	+	3.36	1.84E-07	2.74E-06
Mitotic Metaphase and Anaphase (R-HSA-2555396)	204	24	7.18	+	3.34	2.02E-07	2.97E-06
Signaling by WNT (R-HSA-195721)	196	23	6.9	+	3.33	3.82E-07	5.51E-06
Neddylation (R-HSA-8951664)	175	20	6.16	+	3.25	3.35E-06	4.59E-05
EPH-Ephrin signaling (R-HSA-2682334)	73	8	2.57	+	3.11	3.99E-03	4.43E-02
MAPK family signaling cascades (R-HSA-5683057)	223	24	7.85	+	3.06	1.05E-06	1.46E-05
Signaling by Interleukins (R-HSA-449147)	339	35	11.93	+	2.93	9.64E-09	1.58E-07
Cell Cycle Checkpoints (R-HSA-69620)	233	24	8.2	+	2.93	2.30E-06	3.19E-05
Disease (R-HSA-1643685)	1328	130	46.75	+	2.78	7.23E-30	4.95E-28
Deubiquitination (R-HSA-5688426)	205	20	7.22	+	2.77	3.57E-05	4.71E-04

PIP3 activates AKT signaling (R-HSA-1257604)	198	19	6.97	+	2.73	7.00E-05	9.09E-04
Metabolism of proteins (R-HSA-392499)	1448	135	50.97	+	2.65	5.52E-29	3.58E-27
Class I MHC mediated antigen processing & presentation (R-HSA-983169)	301	28	10.6	+	2.64	2.53E-06	3.49E-05
M Phase (R-HSA-68886)	320	28	11.26	+	2.49	8.23E-06	1.11E-04
Intracellular signaling by second messengers (R-HSA-9006925)	230	20	8.1	+	2.47	1.78E-04	2.19E-03
messengers (R-HSA-5663202)	348	30	12.25	+	2.45	8.36E-06	1.12E-04
Antigen processing: Ubiquitination & Proteasome degradation (R-HSA-983168)	235	20	8.27	+	2.42	2.37E-04	2.86E-03
Metabolism (R-HSA-1430728)	1446	120	50.9	+	2.36	8.42E-21	4.61E-19
Neutrophil degranulation (R-HSA-6798695)	394	32	13.87	+	2.31	9.36E-06	1.24E-04
Innate Immune System (R-HSA-168249)	782	59	27.53	+	2.14	2.46E-08	3.92E-07
Cytokine Signaling in Immune system (R-HSA-1280215)	564	41	19.85	+	2.07	9.35E-06	1.25E-04
Cell Cycle, Mitotic (R-HSA-69278)	445	32	15.66	+	2.04	1.10E-04	1.41E-03
Cell Cycle (R-HSA-1640170)	553	38	19.47	+	1.95	7.15E-05	9.24E-04
Adaptive Immune System (R-HSA-1280218)	587	39	20.66	+	1.89	1.18E-04	1.50E-03
Immune System (R-HSA-168256)	1460	86	51.39	+	1.67	8.11E-07	1.15E-05
RNA Polymerase II Transcription (R-HSA-73857)	954	56	33.58	+	1.67	1.39E-04	1.75E-03
Gene expression (Transcription) (R-HSA-74160)	1093	60	38.47	+	1.56	4.87E-04	5.80E-03
Unclassified (UNCLASSIFIED)	3986	73	140.31	-	0.52	2.64E-14	1.16E-12
Metabolism of lipids (R-HSA-556833)	514	7	18.09	-	0.39	4.37E-03	4.83E-02

Analysis Type:	PANTHER Overrepresentation Test (Released 20240807)						
Annotation Version and Release Date:	Reactome version 86 Released 2023-09-07						
Analyzed List:	DEG week 3 (Homo sapiens)						
Reference List:	Background gene list.txt (Homo sapiens)						
Test Type:	FISHER						
Correction:	FDR		DEG week 3	DEG week 3	DEG week 3	DEG week 3	DEG week 3
Reactome pathways	Background gene list.txt	DEG week 3 (420)	(expected)	(over/ under)	(fold Enrichment)	(raw P-value)	(FDR)
Manipulation of host energy metabolism (R-HSA-9636667)	2	2	0.08	+	25.64	1.52E-03	1.63E-02
Folding of actin by CCT/TriC (R-HSA-390450)	9	6	0.35	+	17.09	2.59E-07	3.41E-06
SARS-CoV-1 modulates host translation machinery (R-HSA-9735869)	35	19	1.37	+	13.92	2.61E-18	1.50E-16
SLBP independent Processing of Histone Pre-mRNAs (R-HSA-111367)	8	4	0.31	+	12.82	1.41E-04	1.70E-03
SARS-CoV-2 modulates host translation machinery (R-HSA-9754678)	48	24	1.87	+	12.82	1.10E-21	7.32E-20
Peptide chain elongation (R-HSA-156902)	87	41	3.39	+	12.08	5.63E-35	1.16E-32
Formation of the ternary complex, and subsequently, the 43S complex (R-HSA-72695)	50	23	1.95	+	11.79	8.93E-20	5.80E-18
Viral mRNA Translation (R-HSA-192823)	87	40	3.39	+	11.79	1.33E-33	2.06E-31
Eukaryotic Translation Termination (R-HSA-72764)	90	41	3.51	+	11.68	3.23E-34	6.12E-32
Eukaryotic Translation Elongation (R-HSA-156842)	90	41	3.51	+	11.68	3.23E-34	5.69E-32
Formation of a pool of free 40S subunits (R-HSA-72689)	99	45	3.86	+	11.65	1.95E-37	1.20E-34
Nonsense Mediated Decay (NMD) independent of the Exon Junction Complex (EJC) (R-HSA-975956)	92	41	3.59	+	11.42	9.88E-34	1.62E-31
SLBP Dependent Processing of Replication-Dependent Histone Pre-mRNAs (R-HSA-77588)	9	4	0.35	+	11.39	2.46E-04	2.90E-03
Selenocysteine synthesis (R-HSA-2408557)	90	40	3.51	+	11.39	7.19E-33	9.85E-31
Formation of ATP by chemiosmotic coupling (R-HSA-163210)	9	4	0.35	+	11.39	2.46E-04	2.89E-03
Somitogenesis (R-HSA-9824272)	44	19	1.72	+	11.07	6.55E-16	3.44E-14
Ribosomal scanning and start codon recognition (R-HSA-72702)	56	24	2.18	+	10.99	1.11E-19	7.03E-18
Translation initiation complex formation (R-HSA-72649)	56	24	2.18	+	10.99	1.11E-19	6.85E-18
Apoptosis induced DNA fragmentation (R-HSA-140342)	7	3	0.27	+	10.99	1.83E-03	1.95E-02
L13a-mediated translational silencing of Ceruloplasmin expression (R-HSA-156827)	108	46	4.21	+	10.92	1.17E-36	5.79E-34
Response of EIF2AK4 (GCN2) to amino acid deficiency (R-HSA-9633012)	94	40	3.67	+	10.91	6.02E-32	6.45E-30
GTP hydrolysis and joining of the 60S ribosomal subunit (R-HSA-72706)	109	46	4.25	+	10.82	1.96E-36	6.91E-34

Activation of the mRNA upon binding of the cap-binding complex and eIFs, and subsequent binding to 43S (R-HSA-72662)	57	24	2.22	+	10.79	1.85E-19	1.11E-17
SRP-dependent cotranslational protein targeting to membrane (R-HSA-1799339)	110	46	4.29	+	10.72	3.26E-36	8.03E-34
AUF1 (hnRNP D0) binds and destabilizes mRNA (R-HSA-450408)	48	20	1.87	+	10.68	2.61E-16	1.40E-14
HSF1 activation (R-HSA-3371511)	12	5	0.47	+	10.68	5.57E-05	6.86E-04
Regulation of expression of SLITs and ROBOs (R-HSA-9010553)	152	62	5.93	+	10.46	6.37E-48	1.57E-44
Cap-dependent Translation Initiation (R-HSA-72737)	116	47	4.52	+	10.39	3.23E-36	9.97E-34
Eukaryotic Translation Initiation (R-HSA-72613)	116	47	4.52	+	10.39	3.23E-36	8.86E-34
Vif-mediated degradation of APOBEC3G (R-HSA-180585)	47	19	1.83	+	10.36	2.91E-15	1.50E-13
Vpu mediated degradation of CD4 (R-HSA-180534)	47	19	1.83	+	10.36	2.91E-15	1.46E-13
Regulation of activated PAK-2p34 by proteasome mediated degradation (R-HSA-211733)	45	18	1.76	+	10.25	1.99E-14	8.30E-13
Selenoamino acid metabolism (R-HSA-2408522)	107	42	4.17	+	10.06	9.45E-32	9.71E-30
Autodegradation of the E3 ubiquitin ligase COP1 (R-HSA-349425)	46	18	1.79	+	10.03	3.15E-14	1.21E-12
Negative regulation of NOTCH4 signaling (R-HSA-9604323)	49	19	1.91	+	9.94	7.32E-15	3.54E-13
GSK3B and BTRC:CUL1-mediated-degradation of NFE2L2 (R-HSA-9762114)	49	19	1.91	+	9.94	7.32E-15	3.47E-13
Cross-presentation of soluble exogenous antigens (endosomes) (R-HSA-1236978)	44	17	1.72	+	9.9	2.12E-13	5.88E-12
Nonsense Mediated Decay (NMD) enhanced by the Exon Junction Complex (EJC) (R-HSA-975957)	112	43	4.37	+	9.84	5.25E-32	6.47E-30
Nonsense-Mediated Decay (NMD) (R-HSA-927802)	112	43	4.37	+	9.84	5.25E-32	6.16E-30
Ubiquitin-dependent degradation of Cyclin D (R-HSA-75815)	47	18	1.83	+	9.82	4.92E-14	1.76E-12
p53-Independent G1/S DNA damage checkpoint (R-HSA-69613)	47	18	1.83	+	9.82	4.92E-14	1.73E-12
p53-Independent DNA Damage Response (R-HSA-69610)	47	18	1.83	+	9.82	4.92E-14	1.71E-12
Ubiquitin Mediated Degradation of Phosphorylated Cdc25A (R-HSA-69601)	47	18	1.83	+	9.82	4.92E-14	1.69E-12
FBXL7 down-regulates AURKA during mitotic entry and in early mitosis (R-HSA-8854050)	50	19	1.95	+	9.74	1.14E-14	5.20E-13
SCF-beta-TrCP mediated degradation of Emi1 (R-HSA-174113)	50	19	1.95	+	9.74	1.14E-14	5.11E-13
Metabolism of polyamines (R-HSA-351202)	50	19	1.95	+	9.74	1.14E-14	5.02E-13
Regulation of ornithine decarboxylase (ODC) (R-HSA-350562)	45	17	1.76	+	9.68	3.29E-13	8.63E-12
Regulation of Apoptosis (R-HSA-169911)	48	18	1.87	+	9.61	7.60E-14	2.50E-12
Degradation of AXIN (R-HSA-4641257)	48	18	1.87	+	9.61	7.60E-14	2.46E-12
Degradation of GLI2 by the proteasome (R-HSA-5610783)	52	19	2.03	+	9.37	2.66E-14	1.09E-12
Degradation of GLI1 by the proteasome (R-HSA-5610780)	52	19	2.03	+	9.37	2.66E-14	1.08E-12
Regulation of RUNX3 expression and activity (R-HSA-8941858)	50	18	1.95	+	9.23	1.75E-13	5.12E-12
Hh mutants are degraded by ERAD (R-HSA-5362768)	50	18	1.95	+	9.23	1.75E-13	5.06E-12

Stabilization of p53 (R-HSA-69541)	50	18	1.95	+	9.23	1.75E-13	5.00E-12
Degradation of DVL (R-HSA-4641258)	50	18	1.95	+	9.23	1.75E-13	4.95E-12
GLI3 is processed to GLI3R by the proteasome (R-HSA-5610785)	53	19	2.07	+	9.19	4.00E-14	1.47E-12
NIK-->noncanonical NF-kB signaling (R-HSA-5676590)	53	19	2.07	+	9.19	4.00E-14	1.45E-12
Asymmetric localization of PCP proteins (R-HSA-4608870)	51	18	1.99	+	9.05	2.60E-13	6.97E-12
Formation of paraxial mesoderm (R-HSA-9793380)	54	19	2.11	+	9.02	5.96E-14	2.01E-12
Dectin-1 mediated noncanonical NF-kB signaling (R-HSA-5607761)	54	19	2.11	+	9.02	5.96E-14	1.98E-12
Prefoldin mediated transfer of substrate to CCT/TriC (R-HSA-389957)	23	8	0.9	+	8.92	1.47E-06	1.90E-05
Hh mutants abrogate ligand secretion (R-HSA-5387390)	52	18	2.03	+	8.87	3.84E-13	9.66E-12
SCF(Skp2)-mediated degradation of p27/p21 (R-HSA-187577)	55	19	2.15	+	8.86	8.78E-14	2.81E-12
Signaling by ROBO receptors (R-HSA-376176)	189	65	7.37	+	8.82	1.13E-44	1.40E-41
Gene and protein expression by JAK-STAT signaling after Interleukin-12 stimulation (R-HSA-8950505)	35	12	1.37	+	8.79	3.92E-09	5.72E-08
Cellular response to starvation (R-HSA-9711097)	135	46	5.27	+	8.74	1.77E-31	1.68E-29
Oxygen-dependent proline hydroxylation of Hypoxia-inducible Factor Alpha (R-HSA-1234176)	59	20	2.3	+	8.69	2.93E-14	1.15E-12
Hedgehog ligand biogenesis (R-HSA-5358346)	54	18	2.11	+	8.55	8.11E-13	1.89E-11
Integration of provirus (R-HSA-162592)	9	3	0.35	+	8.55	4.15E-03	4.16E-02
Defective CFTR causes cystic fibrosis (R-HSA-5678895)	55	18	2.15	+	8.39	1.16E-12	2.58E-11
Influenza Viral RNA Transcription and Replication (R-HSA-168273)	130	42	5.07	+	8.28	9.86E-28	8.10E-26
Regulation of RUNX2 expression and activity (R-HSA-8939902)	59	19	2.3	+	8.26	3.78E-13	9.62E-12
RUNX1 regulates transcription of genes involved in differentiation of HSCs (R-HSA-8939236)	66	21	2.57	+	8.16	2.79E-14	1.11E-12
APC/C:Cdc20 mediated degradation of Securin (R-HSA-174154)	63	20	2.46	+	8.14	1.22E-13	3.68E-12
ER-Phagosome pathway (R-HSA-1236974)	79	25	3.08	+	8.11	1.06E-16	5.84E-15
p53-Dependent G1/S DNA damage checkpoint (R-HSA-69580)	57	18	2.22	+	8.1	2.33E-12	5.04E-11
p53-Dependent G1 DNA Damage Response (R-HSA-69563)	57	18	2.22	+	8.1	2.33E-12	5.00E-11
Chaperone Mediated Autophagy (R-HSA-9613829)	19	6	0.74	+	8.1	5.98E-05	7.34E-04
Regulation of PTEN stability and activity (R-HSA-8948751)	61	19	2.38	+	7.98	7.48E-13	1.79E-11
Activation of NF-kappaB in B cells (R-HSA-1169091)	61	19	2.38	+	7.98	7.48E-13	1.77E-11
Autodegradation of Cdh1 by Cdh1:APC/C (R-HSA-174084)	61	19	2.38	+	7.98	7.48E-13	1.76E-11
Regulation of RAS by GAPs (R-HSA-5658442)	58	18	2.26	+	7.96	3.26E-12	6.81E-11
SARS-CoV-1-host interactions (R-HSA-9692914)	84	26	3.28	+	7.93	4.75E-17	2.66E-15

Cellular response to hypoxia (R-HSA-1234174)	65	20	2.54	+	7.89	2.39E-13	6.55E-12
Cooperation of Prefoldin and TriC/CCT in actin and tubulin folding (R-HSA-389958)	26	8	1.01	+	7.89	4.22E-06	5.36E-05
G1/S DNA Damage Checkpoints (R-HSA-69615)	59	18	2.3	+	7.82	4.53E-12	9.15E-11
APC/C:Cdc20 mediated degradation of mitotic proteins (R-HSA-176409)	70	21	2.73	+	7.69	1.05E-13	3.23E-12
Cdc20:Phospho-APC/C mediated degradation of Cyclin A (R-HSA-174184)	67	20	2.61	+	7.65	4.55E-13	1.12E-11
Influenza Infection (R-HSA-168255)	148	44	5.77	+	7.62	2.65E-27	2.11E-25
Orc1 removal from chromatin (R-HSA-68949)	64	19	2.5	+	7.61	1.97E-12	4.30E-11
Activation of APC/C and APC/C:Cdc20 mediated degradation of mitotic proteins (R-HSA-176814)	71	21	2.77	+	7.58	1.43E-13	4.26E-12
checkpoint (R-HSA-179419)	68	20	2.65	+	7.54	6.21E-13	1.50E-11
Interleukin-12 signaling (R-HSA-9020591)	41	12	1.6	+	7.5	3.00E-08	4.07E-07
Signaling by NOTCH4 (R-HSA-9013694)	72	21	2.81	+	7.48	1.95E-13	5.47E-12
ABC transporter disorders (R-HSA-5619084)	62	18	2.42	+	7.44	1.16E-11	2.13E-10
late mitosis/early G1 (R-HSA-174178)	69	20	2.69	+	7.43	8.44E-13	1.93E-11
The role of GTSE1 in G2/M progression after G2 checkpoint (R-HSA-8852276)	66	19	2.57	+	7.38	3.64E-12	7.48E-11
Nuclear events mediated by NFE2L2 (R-HSA-9759194)	80	23	3.12	+	7.37	1.91E-14	8.14E-13
PCP/CE pathway (R-HSA-4086400)	70	20	2.73	+	7.32	1.14E-12	2.55E-11
Hedgehog 'on' state (R-HSA-5632684)	67	19	2.61	+	7.27	4.90E-12	9.74E-11
CDK-mediated phosphorylation and removal of Cdc6 (R-HSA-69017)	67	19	2.61	+	7.27	4.90E-12	9.67E-11
Regulation of mRNA stability by proteins that bind AU-rich elements (R-HSA-450531)	78	22	3.04	+	7.23	1.11E-13	3.38E-12
Regulation of APC/C activators between G1/S and early anaphase (R-HSA-176408)	75	21	2.93	+	7.18	4.77E-13	1.16E-11
TNFR2 non-canonical NF-kB pathway (R-HSA-5668541)	69	19	2.69	+	7.06	8.73E-12	1.67E-10
Downstream signaling events of B Cell Receptor (BCR) (R-HSA-1168372)	73	20	2.85	+	7.02	2.71E-12	5.77E-11
FCERI mediated NF-kB activation (R-HSA-2871837)	77	21	3	+	6.99	8.43E-13	1.94E-11
Degradation of beta-catenin by the destruction complex (R-HSA-195253)	74	20	2.89	+	6.93	3.59E-12	7.44E-11
Regulation of mitotic cell cycle (R-HSA-453276)	82	22	3.2	+	6.88	3.45E-13	8.95E-12
APC/C-mediated degradation of cell cycle proteins (R-HSA-174143)	82	22	3.2	+	6.88	3.45E-13	8.85E-12
Cyclin E associated events during G1/S transition (R-HSA-69202)	75	20	2.93	+	6.84	4.72E-12	9.46E-11
Antigen processing-Cross presentation (R-HSA-1236975)	94	25	3.67	+	6.82	1.02E-14	4.73E-13
Formation of tubulin folding intermediates by CCT/TriC (R-HSA-389960)	19	5	0.74	+	6.75	6.51E-04	7.44E-03
Major pathway of rRNA processing in the nucleolus and cytosol (R-HSA-6791226)	169	44	6.59	+	6.67	1.16E-24	7.93E-23
Cyclin A:Cdk2-associated events at S phase entry (R-HSA-69656)	77	20	3	+	6.66	8.05E-12	1.55E-10

rRNA processing in the nucleus and cytosol (R-HSA-8868773)	179	46	6.98	+	6.59	1.75E-25	1.27E-23
Gastrulation (R-HSA-9758941)	74	19	2.89	+	6.58	3.38E-11	5.95E-10
Interleukin-12 family signaling (R-HSA-447115)	47	12	1.83	+	6.55	1.60E-07	2.13E-06
Assembly of the pre-replicative complex (R-HSA-68867)	83	21	3.24	+	6.49	4.17E-12	8.49E-11
Condensation of Prophase Chromosomes (R-HSA-2299718)	16	4	0.62	+	6.41	2.86E-03	2.95E-02
Infection with Mycobacterium tuberculosis (R-HSA-9635486)	24	6	0.94	+	6.41	2.51E-04	2.94E-03
MAPK6/MAPK4 signaling (R-HSA-5687128)	76	19	2.96	+	6.41	5.61E-11	9.74E-10
rRNA processing (R-HSA-72312)	185	46	7.22	+	6.37	8.08E-25	5.69E-23
mRNA Splicing - Minor Pathway (R-HSA-72165)	49	12	1.91	+	6.28	2.64E-07	3.46E-06
UCH proteinases (R-HSA-5689603)	78	19	3.04	+	6.24	9.16E-11	1.54E-09
RHOBTB GTPase Cycle (R-HSA-9706574)	33	8	1.29	+	6.21	2.95E-05	3.67E-04
CLEC7A (Dectin-1) signaling (R-HSA-5607764)	87	21	3.39	+	6.19	1.11E-11	2.10E-10
Downstream TCR signaling (R-HSA-202424)	87	21	3.39	+	6.19	1.11E-11	2.08E-10
Cristae formation (R-HSA-8949613)	21	5	0.82	+	6.1	1.07E-03	1.18E-02
Smooth Muscle Contraction (R-HSA-445355)	34	8	1.33	+	6.03	3.72E-05	4.62E-04
Switching of origins to a post-replicative state (R-HSA-69052)	85	20	3.32	+	6.03	5.75E-11	9.92E-10
Transcriptional regulation by RUNX3 (R-HSA-8878159)	85	20	3.32	+	6.03	5.75E-11	9.85E-10
Translation (R-HSA-72766)	271	63	10.57	+	5.96	5.36E-32	6.00E-30
Metabolism of amino acids and derivatives (R-HSA-71291)	274	63	10.69	+	5.89	1.06E-31	1.05E-29
SARS-CoV-1 Infection (R-HSA-9678108)	122	28	4.76	+	5.88	1.49E-14	6.46E-13
Response of Mtb to phagocytosis (R-HSA-9637690)	22	5	0.86	+	5.83	1.34E-03	1.47E-02
RHOBTB1 GTPase cycle (R-HSA-9013422)	22	5	0.86	+	5.83	1.34E-03	1.46E-02
RHOBTB2 GTPase cycle (R-HSA-9013418)	22	5	0.86	+	5.83	1.34E-03	1.46E-02
KEAP1-NFE2L2 pathway (R-HSA-9755511)	106	24	4.13	+	5.8	1.63E-12	3.60E-11
Host Interactions of HIV factors (R-HSA-162909)	116	26	4.52	+	5.75	2.43E-13	6.58E-12
Hedgehog 'off' state (R-HSA-5610787)	90	20	3.51	+	5.7	1.74E-10	2.89E-09
Transferrin endocytosis and recycling (R-HSA-917977)	18	4	0.7	+	5.7	4.52E-03	4.49E-02
DNA Replication Pre-Initiation (R-HSA-69002)	99	22	3.86	+	5.7	2.10E-11	3.81E-10
Signaling by the B Cell Receptor (BCR) (R-HSA-983705)	99	22	3.86	+	5.7	2.10E-11	3.78E-10
Interleukin-1 signaling (R-HSA-9020702)	100	22	3.9	+	5.64	2.60E-11	4.61E-10
ABC-family proteins mediated transport (R-HSA-382556)	83	18	3.24	+	5.56	2.20E-09	3.31E-08
E3 ubiquitin ligases ubiquitinate target proteins (R-HSA-8866654)	37	8	1.44	+	5.54	7.15E-05	8.72E-04

Gluconeogenesis (R-HSA-70263)	28	6	1.09	+	5.49	6.16E-04	7.07E-03
Apoptosis (R-HSA-109581)	136	29	5.31	+	5.47	3.88E-14	1.45E-12
Transcriptional regulation by RUNX2 (R-HSA-8878166)	92	19	3.59	+	5.29	1.88E-09	2.87E-08
Late endosomal microautophagy (R-HSA-9615710)	25	5	0.98	+	5.13	2.45E-03	2.56E-02
MAP2K and MAPK activation (R-HSA-5674135)	30	6	1.17	+	5.13	9.09E-04	1.01E-02
DNA Replication (R-HSA-69306)	122	24	4.76	+	5.04	3.98E-11	6.97E-10
Synthesis of DNA (R-HSA-69239)	112	22	4.37	+	5.04	2.71E-10	4.43E-09
TCR signaling (R-HSA-202403)	107	21	4.17	+	5.03	7.08E-10	1.12E-08
SARS-CoV-2-host interactions (R-HSA-9705683)	166	32	6.48	+	4.94	3.65E-14	1.38E-12
Programmed Cell Death (R-HSA-5357801)	161	31	6.28	+	4.94	9.60E-14	3.03E-12
Cooperation of PDCL (PhLP1) and TRiC/CCT in G-protein beta folding (R-HSA-6814122)	26	5	1.01	+	4.93	2.94E-03	3.02E-02
Axon guidance (R-HSA-422475)	401	77	15.64	+	4.92	5.04E-33	7.31E-31
Fc epsilon receptor (FCERI) signaling (R-HSA-2454202)	117	22	4.56	+	4.82	6.55E-10	1.04E-08
Beta-catenin independent WNT signaling (R-HSA-3858494)	112	21	4.37	+	4.81	1.70E-09	2.63E-08
Nervous system development (R-HSA-9675108)	417	78	16.27	+	4.8	1.24E-32	1.61E-30
Interleukin-1 family signaling (R-HSA-446652)	123	23	4.8	+	4.79	2.99E-10	4.84E-09
Signaling by high-kinase activity BRAF mutants (R-HSA-6802948)	27	5	1.05	+	4.75	3.50E-03	3.58E-02
C-type lectin receptors (CLRs) (R-HSA-5621481)	119	22	4.64	+	4.74	9.19E-10	1.43E-08
G1/S Transition (R-HSA-69206)	120	22	4.68	+	4.7	1.09E-09	1.68E-08
Signaling by Hedgehog (R-HSA-5358351)	115	21	4.49	+	4.68	2.82E-09	4.16E-08
PTEN Regulation (R-HSA-6807070)	123	22	4.8	+	4.59	1.77E-09	2.71E-08
B-WICH complex positively regulates rRNA expression (R-HSA-5250924)	28	5	1.09	+	4.58	4.12E-03	4.15E-02
TCF dependent signaling in response to WNT (R-HSA-201681)	124	22	4.84	+	4.55	2.07E-09	3.14E-08
Signaling by NOTCH (R-HSA-157118)	147	26	5.73	+	4.53	7.41E-11	1.25E-09
Apoptotic execution phase (R-HSA-75153)	34	6	1.33	+	4.52	1.81E-03	1.93E-02
mRNA Splicing - Major Pathway (R-HSA-72163)	190	33	7.41	+	4.45	3.22E-13	8.53E-12
G2/M Checkpoints (R-HSA-69481)	123	21	4.8	+	4.38	9.92E-09	1.42E-07
Iron uptake and transport (R-HSA-917937)	41	7	1.6	+	4.38	9.31E-04	1.03E-02
S Phase (R-HSA-69242)	150	25	5.85	+	4.27	6.43E-10	1.03E-08
EPHB-mediated forward signaling (R-HSA-3928662)	36	6	1.4	+	4.27	2.45E-03	2.57E-02
mRNA Splicing (R-HSA-72172)	198	33	7.72	+	4.27	1.07E-12	2.42E-11
Separation of Sister Chromatids (R-HSA-2467813)	168	28	6.55	+	4.27	5.88E-11	1.00E-09

Mitotic G1 phase and G1/S transition (R-HSA-453279)	134	22	5.23	+	4.21	9.32E-09	1.34E-07
Metabolism of RNA (R-HSA-8953854)	646	105	25.2	+	4.17	1.28E-38	1.05E-35
Ub-specific processing proteases (R-HSA-5689880)	143	23	5.58	+	4.12	6.42E-09	9.31E-08
Protein ubiquitination (R-HSA-8852135)	56	9	2.18	+	4.12	2.85E-04	3.31E-03
rRNA modification in the nucleus and cytosol (R-HSA-6790901)	56	9	2.18	+	4.12	2.85E-04	3.30E-03
Cellular response to chemical stress (R-HSA-9711123)	175	28	6.83	+	4.1	1.58E-10	2.64E-09
HIV Infection (R-HSA-162906)	202	32	7.88	+	4.06	9.76E-12	1.85E-10
SARS-CoV-2 Infection (R-HSA-9694516)	240	38	9.36	+	4.06	1.02E-13	3.20E-12
Mitotic Anaphase (R-HSA-68882)	203	32	7.92	+	4.04	1.12E-11	2.07E-10
Mitotic Metaphase and Anaphase (R-HSA-2555396)	204	32	7.96	+	4.02	1.28E-11	2.34E-10
Viral Infection Pathways (R-HSA-9824446)	653	102	25.47	+	4	7.11E-36	1.59E-33
Cellular responses to stress (R-HSA-2262752)	623	93	24.3	+	3.83	6.09E-31	5.56E-29
Transcriptional regulation by RUNX1 (R-HSA-8878171)	149	22	5.81	+	3.79	6.79E-08	9.15E-07
RNA Polymerase II Transcription Termination (R-HSA-73856)	61	9	2.38	+	3.78	5.49E-04	6.33E-03
Cellular responses to stimuli (R-HSA-8953897)	631	93	24.61	+	3.78	1.70E-30	1.49E-28
Amino acids regulate mTORC1 (R-HSA-9639288)	41	6	1.6	+	3.75	4.80E-03	4.73E-02
production by uncoupling proteins. (R-HSA-163200)	96	14	3.74	+	3.74	1.96E-05	2.45E-04
Disorders of transmembrane transporters (R-HSA-5619115)	124	18	4.84	+	3.72	1.38E-06	1.80E-05
Infectious disease (R-HSA-5663205)	798	114	31.13	+	3.66	1.48E-36	6.10E-34
Neddylation (R-HSA-8951664)	175	25	6.83	+	3.66	1.69E-08	2.37E-07
G2/M Transition (R-HSA-69275)	175	25	6.83	+	3.66	1.69E-08	2.36E-07
Mitotic G2-G2/M phases (R-HSA-453274)	176	25	6.87	+	3.64	1.90E-08	2.63E-07
SARS-CoV Infections (R-HSA-9679506)	340	48	13.26	+	3.62	4.75E-15	2.34E-13
Platelet degranulation (R-HSA-114608)	87	12	3.39	+	3.54	1.33E-04	1.60E-03
Signaling by WNT (R-HSA-195721)	196	27	7.65	+	3.53	9.97E-09	1.42E-07
RAF/MAP kinase cascade (R-HSA-5673001)	189	26	7.37	+	3.53	1.94E-08	2.67E-07
MAPK1/MAPK3 signaling (R-HSA-5684996)	192	26	7.49	+	3.47	2.70E-08	3.67E-07
Signaling by BRAF and RAF1 fusions (R-HSA-6802952)	52	7	2.03	+	3.45	3.83E-03	3.88E-02
Developmental Biology (R-HSA-1266738)	682	91	26.6	+	3.42	1.71E-26	1.28E-24
Class I MHC mediated antigen processing & presentation (R-HSA-983169)	301	40	11.74	+	3.41	7.51E-12	1.46E-10
Antigen processing: Ubiquitination & Proteasome degradation (R-HSA-983168)	235	31	9.17	+	3.38	2.29E-09	3.42E-08
Response to elevated platelet cytosolic Ca2+ (R-HSA-76005)	91	12	3.55	+	3.38	2.05E-04	2.45E-03

Processing of Capped Intron-Containing Pre-mRNA (R-HSA-72203)	260	34	10.14	+	3.35	4.70E-10	7.57E-09
Bacterial Infection Pathways (R-HSA-9824439)	54	7	2.11	+	3.32	4.75E-03	4.70E-02
Signaling by Interleukins (R-HSA-449147)	339	42	13.22	+	3.18	2.22E-11	3.97E-10
Global Genome Nucleotide Excision Repair (GG-NER) (R-HSA-5696399)	81	10	3.16	+	3.16	1.16E-03	1.27E-02
TP53 Regulates Metabolic Genes (R-HSA-5628897)	73	9	2.85	+	3.16	2.04E-03	2.16E-02
EPH-Ephrin signaling (R-HSA-2682334)	73	9	2.85	+	3.16	2.04E-03	2.15E-02
M Phase (R-HSA-68886)	320	39	12.48	+	3.12	1.98E-10	3.26E-09
MAPK family signaling cascades (R-HSA-5683057)	223	27	8.7	+	3.1	1.55E-07	2.07E-06
Cell Cycle Checkpoints (R-HSA-69620)	233	28	9.09	+	3.08	1.07E-07	1.43E-06
Respiratory electron transport (R-HSA-611105)	85	10	3.32	+	3.02	1.68E-03	1.80E-02
Deubiquitination (R-HSA-5688426)	205	24	8	+	3	1.42E-06	1.84E-05
Nucleotide Excision Repair (R-HSA-5696398)	103	12	4.02	+	2.99	6.52E-04	7.41E-03
PIP3 activates AKT signaling (R-HSA-1257604)	198	23	7.72	+	2.98	2.70E-06	3.45E-05
Cellular response to heat stress (R-HSA-3371556)	80	9	3.12	+	2.88	3.83E-03	3.89E-02
Diseases of signal transduction by growth factor receptors and second messengers (R-HSA-5663202)	348	39	13.57	+	2.87	2.34E-09	3.47E-08
Mitochondrial protein degradation (R-HSA-9837999)	81	9	3.16	+	2.85	4.17E-03	4.16E-02
Neutrophil degranulation (R-HSA-6798695)	394	43	15.37	+	2.8	7.70E-10	1.21E-08
Intracellular signaling by second messengers (R-HSA-9006925)	230	24	8.97	+	2.68	1.07E-05	1.35E-04
The citric acid (TCA) cycle and respiratory electron transport (R-HSA-1428517)	137	14	5.34	+	2.62	9.05E-04	1.01E-02
Disease (R-HSA-1643685)	1328	133	51.8	+	2.57	1.17E-26	9.05E-25
Metabolism of proteins (R-HSA-392499)	1448	145	56.48	+	2.57	1.94E-29	1.65E-27
Platelet activation, signaling and aggregation (R-HSA-76002)	180	18	7.02	+	2.56	2.31E-04	2.74E-03
Cell Cycle, Mitotic (R-HSA-69278)	445	44	17.36	+	2.53	1.05E-08	1.49E-07
Autophagy (R-HSA-9612973)	122	12	4.76	+	2.52	2.85E-03	2.95E-02
Clathrin-mediated endocytosis (R-HSA-8856828)	114	11	4.45	+	2.47	4.86E-03	4.78E-02
Protein localization (R-HSA-9609507)	126	12	4.92	+	2.44	3.72E-03	3.79E-02
Innate Immune System (R-HSA-168249)	782	74	30.5	+	2.43	4.25E-13	1.06E-11
Adaptive Immune System (R-HSA-1280218)	587	54	22.9	+	2.36	3.59E-09	5.26E-08
Cytokine Signaling in Immune system (R-HSA-1280215)	564	51	22	+	2.32	1.60E-08	2.25E-07
Cell Cycle (R-HSA-1640170)	553	50	21.57	+	2.32	2.29E-08	3.14E-07
RHO GTPase Effectors (R-HSA-195258)	226	19	8.82	+	2.16	1.47E-03	1.59E-02

Metabolism (R-HSA-1430728)	1446	109	56.41	+	1.93	2.73E-12	5.75E-11
Immune System (R-HSA-168256)	1460	109	56.95	+	1.91	5.70E-12	1.12E-10
Hemostasis (R-HSA-109582)	412	29	16.07	+	1.8	2.47E-03	2.57E-02
Signaling by Rho GTPases (R-HSA-194315)	532	37	20.75	+	1.78	7.43E-04	8.41E-03
Signaling by Rho GTPases, Miro GTPases and RHOBTB3 (R-HSA-9716542)	545	37	21.26	+	1.74	9.03E-04	1.02E-02
Post-translational protein modification (R-HSA-597592)	986	66	38.46	+	1.72	1.29E-05	1.63E-04
Transport of small molecules (R-HSA-382551)	436	29	17.01	+	1.71	5.00E-03	4.89E-02
RNA Polymerase II Transcription (R-HSA-73857)	954	60	37.21	+	1.61	2.08E-04	2.48E-03
Gene expression (Transcription) (R-HSA-74160)	1093	68	42.64	+	1.59	9.50E-05	1.15E-03
Signal Transduction (R-HSA-162582)	1626	100	63.43	+	1.58	1.89E-06	2.43E-05
Unclassified (UNCLASSIFIED)	3986	73	155.49	-	0.47	3.58E-19	2.10E-17
