# Supplementary figures and images for: RAG recombinase expression discriminates the development of natural killer cells
Source: Front Immunol. 2025 Jul 25;16:1607664. doi: 10.3389/fimmu.2025.1607664 (PMC12331628; doi:10.3389/fimmu.2025.1607664)

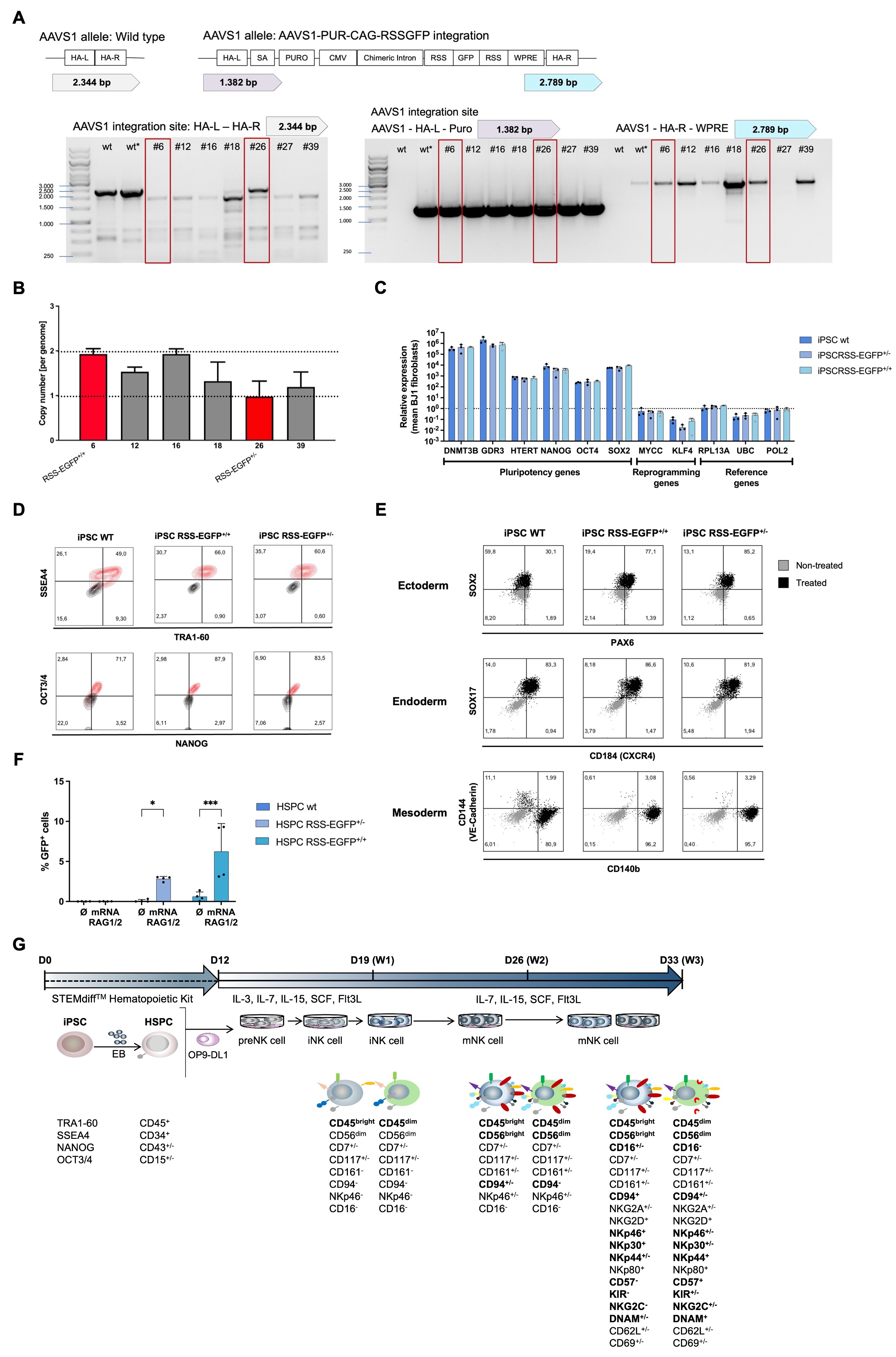

Supplement: Supplementary file 3 [file Image1.jpeg]

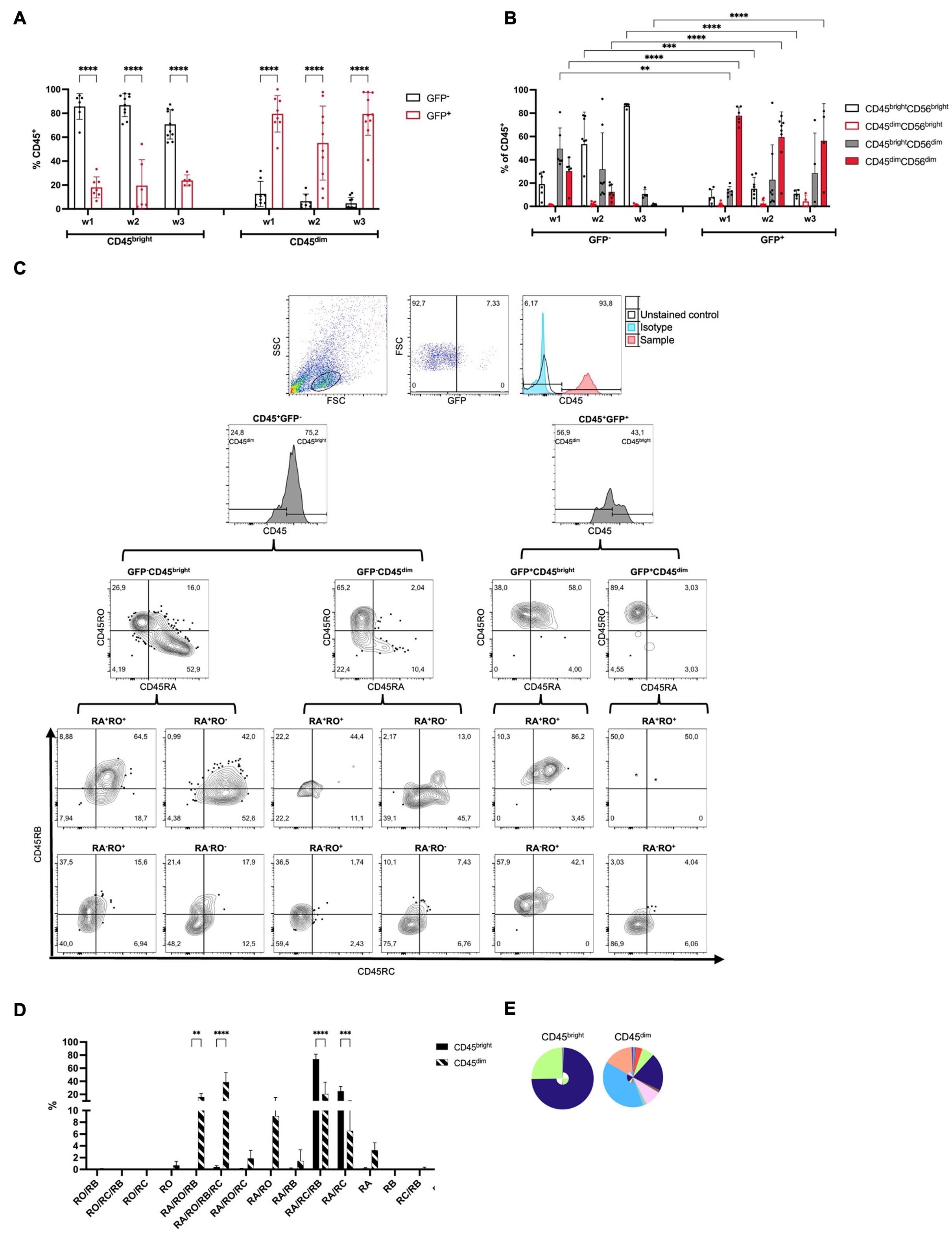

Supplement: Supplementary file 4 [file Image2.jpeg]

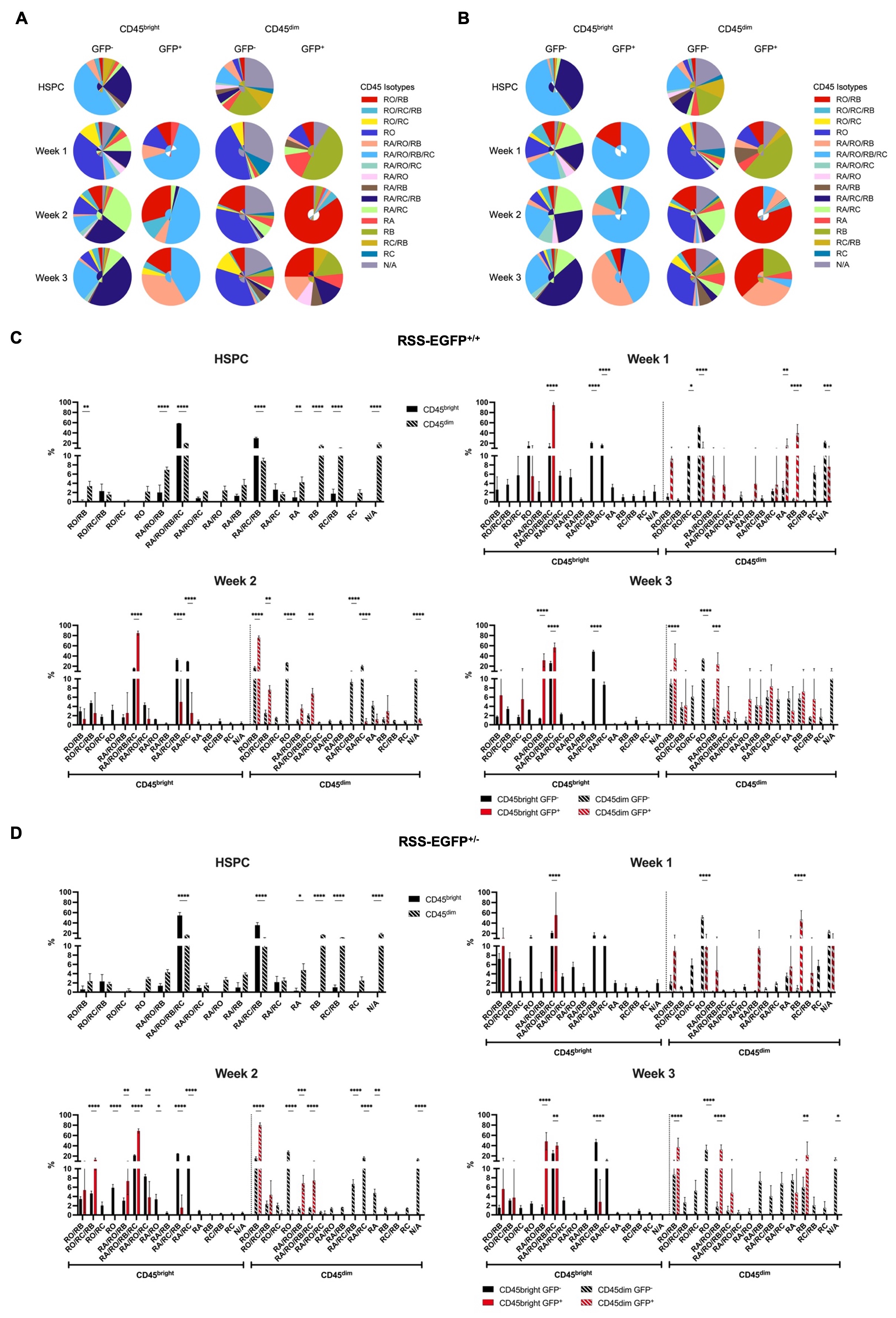

Supplement: Supplementary file 5 [file Image3.jpeg]

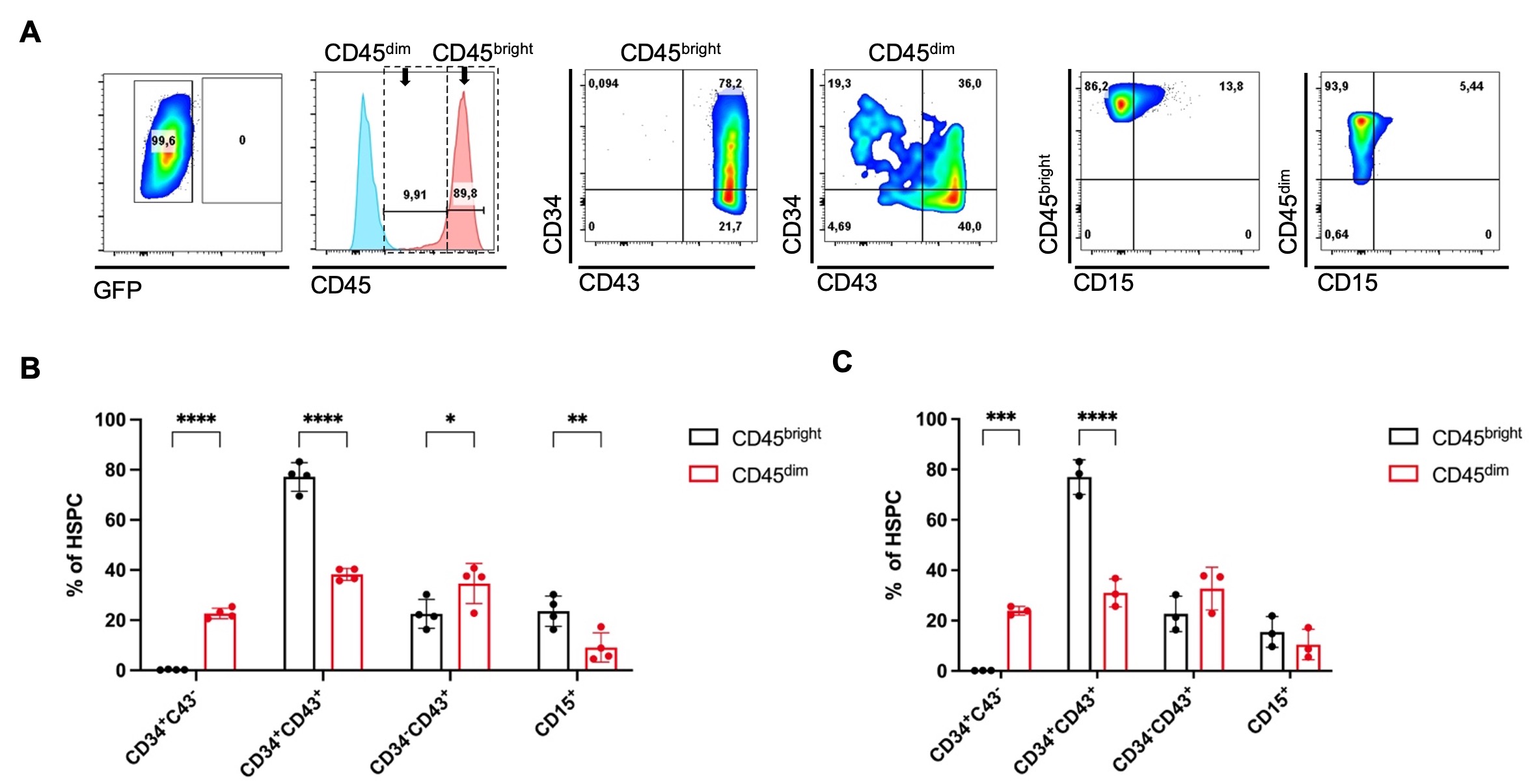

Supplement: Supplementary file 6 [file Image4.jpeg]

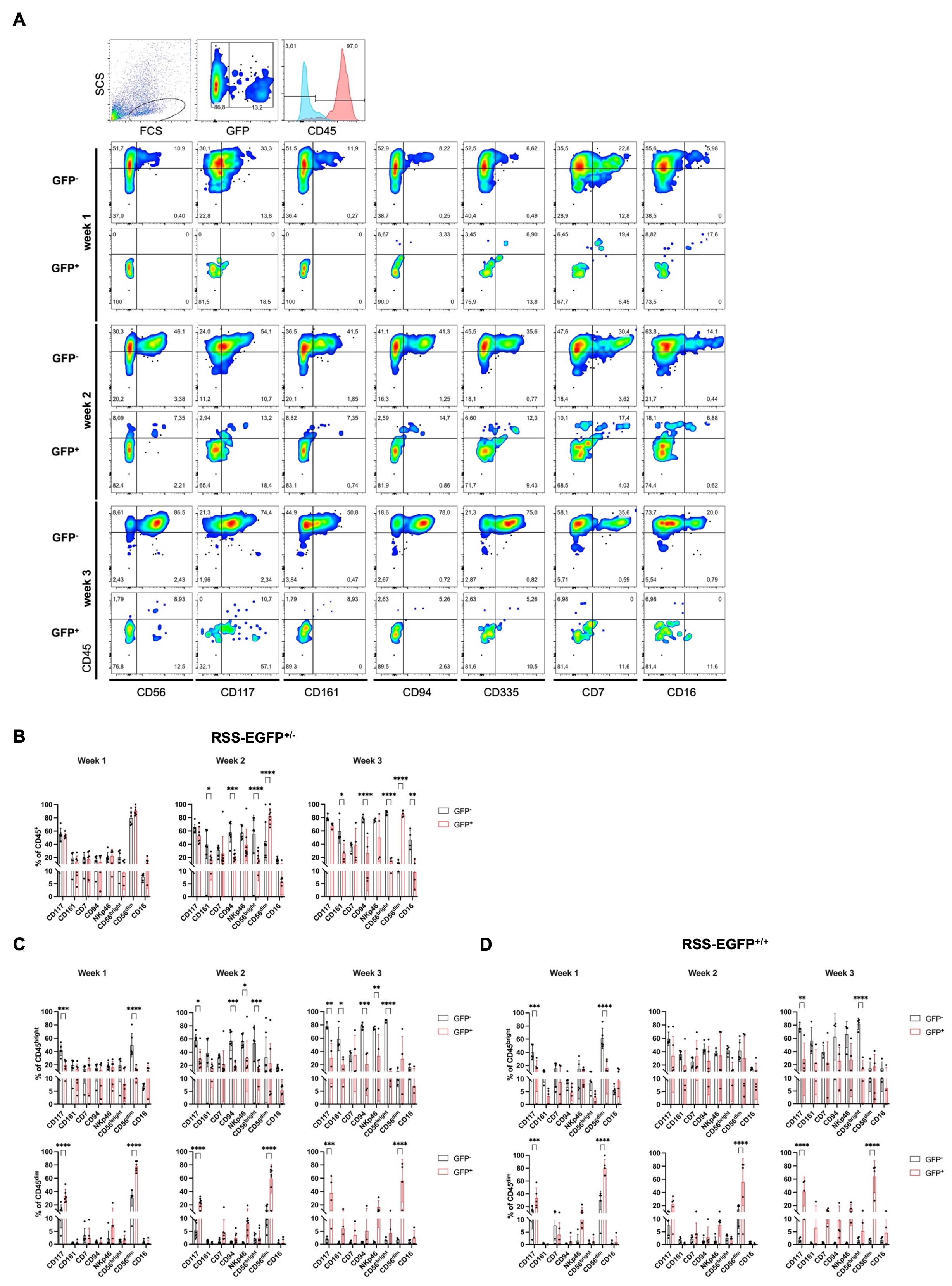

Supplement: Supplementary file 7 [file Image5.jpeg]

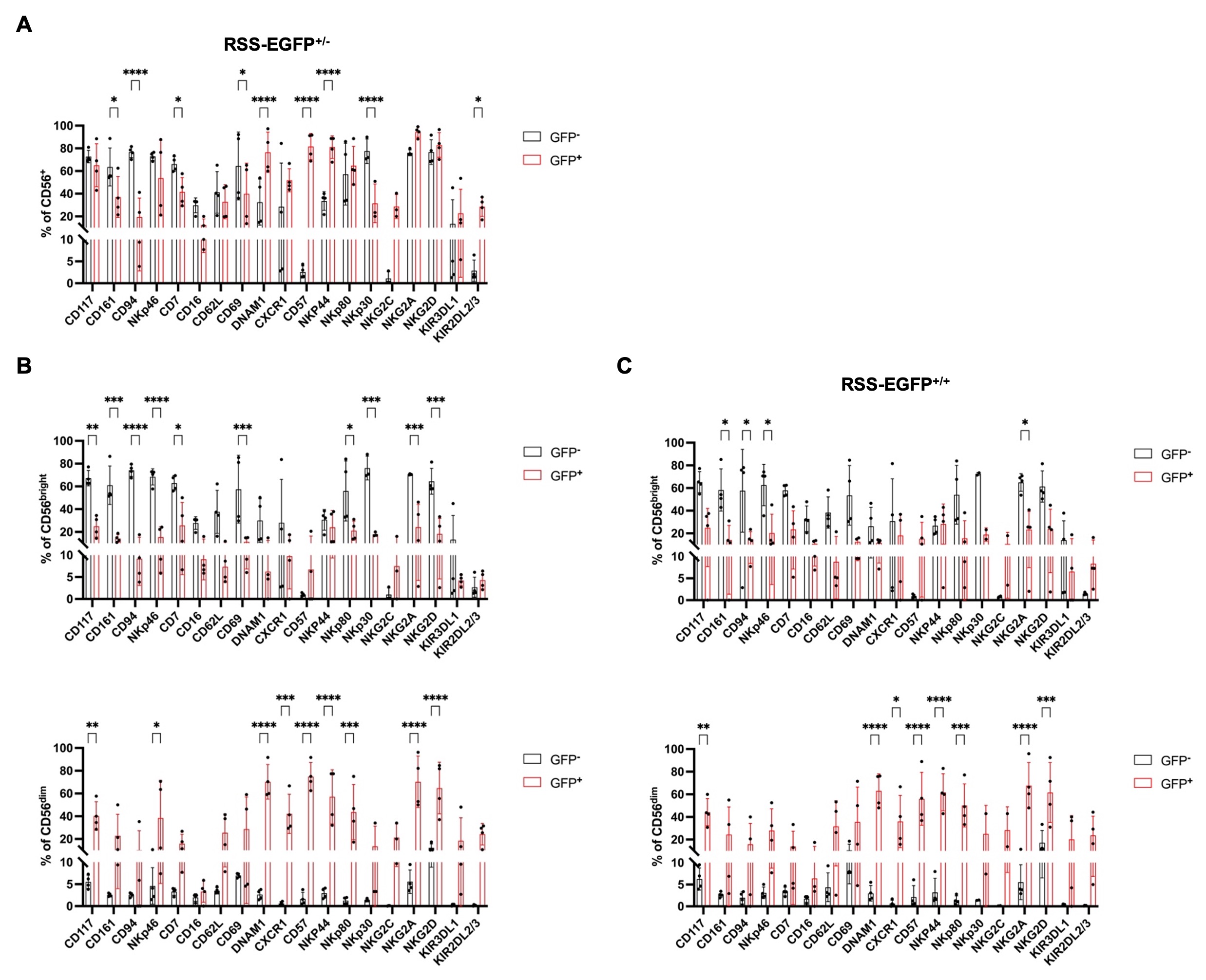

Supplement: Supplementary file 8 [file Image6.jpeg]

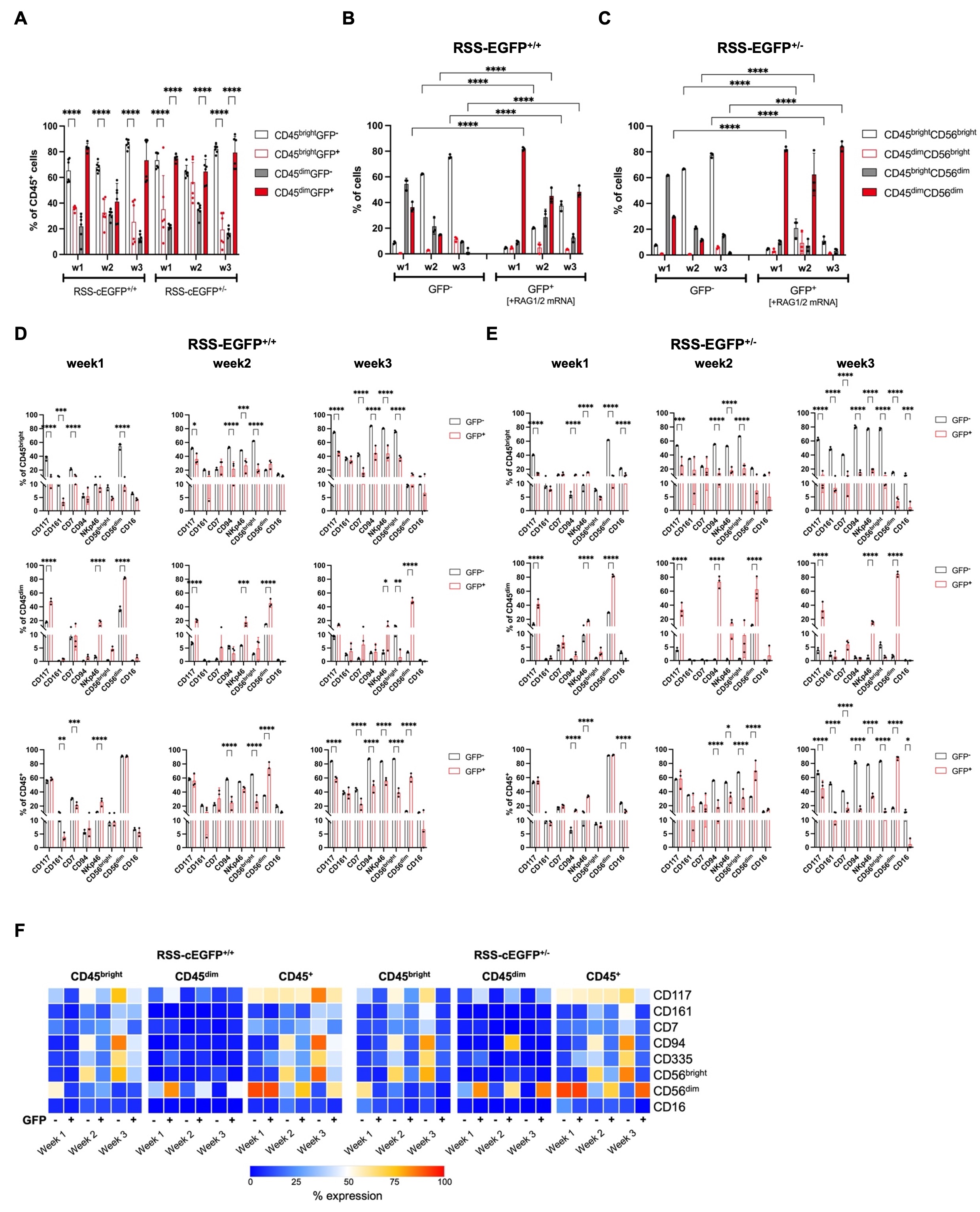

Supplement: Supplementary file 9 [file Image7.jpeg]

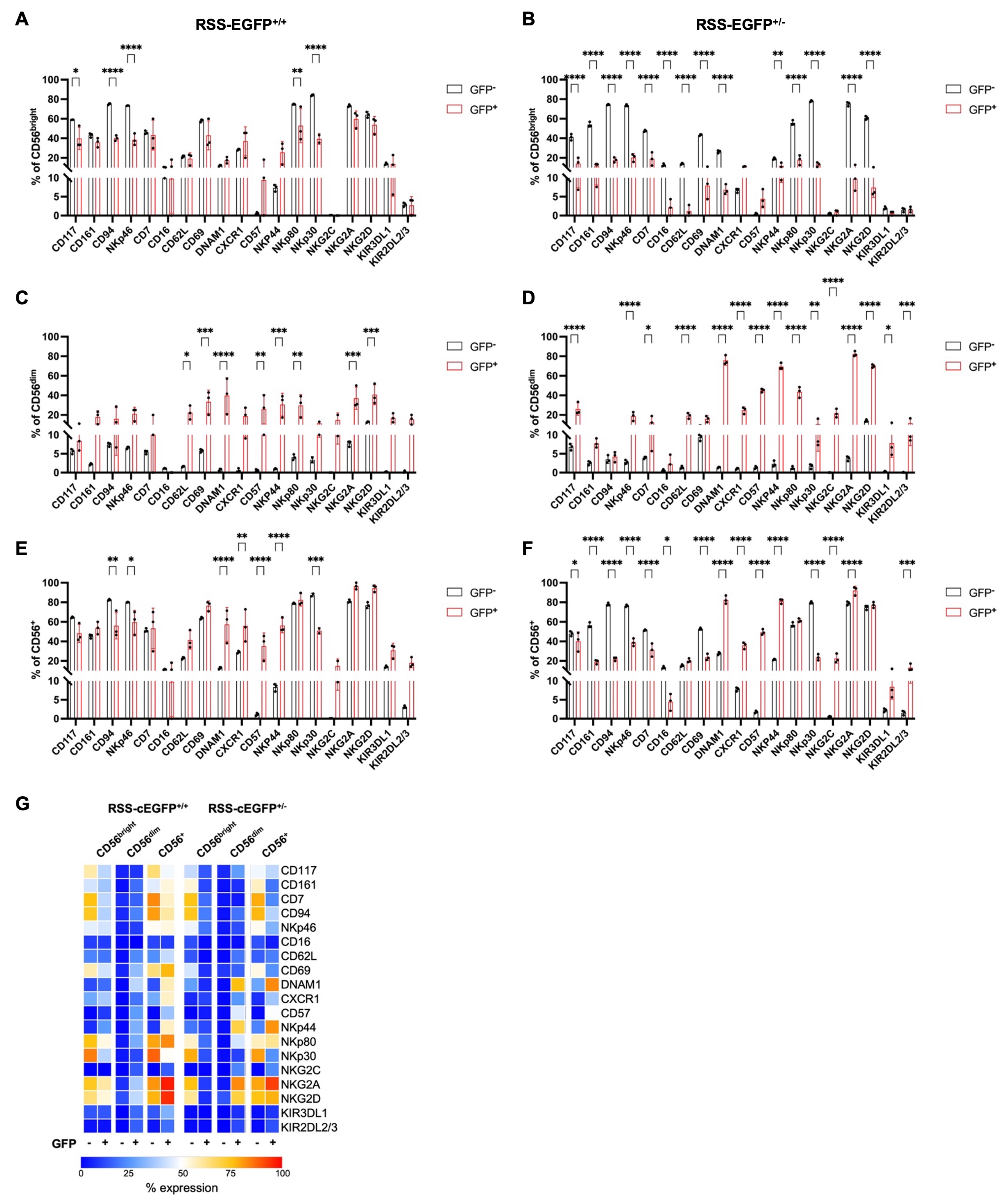

Supplement: Supplementary file 10 [file Image8.jpeg]

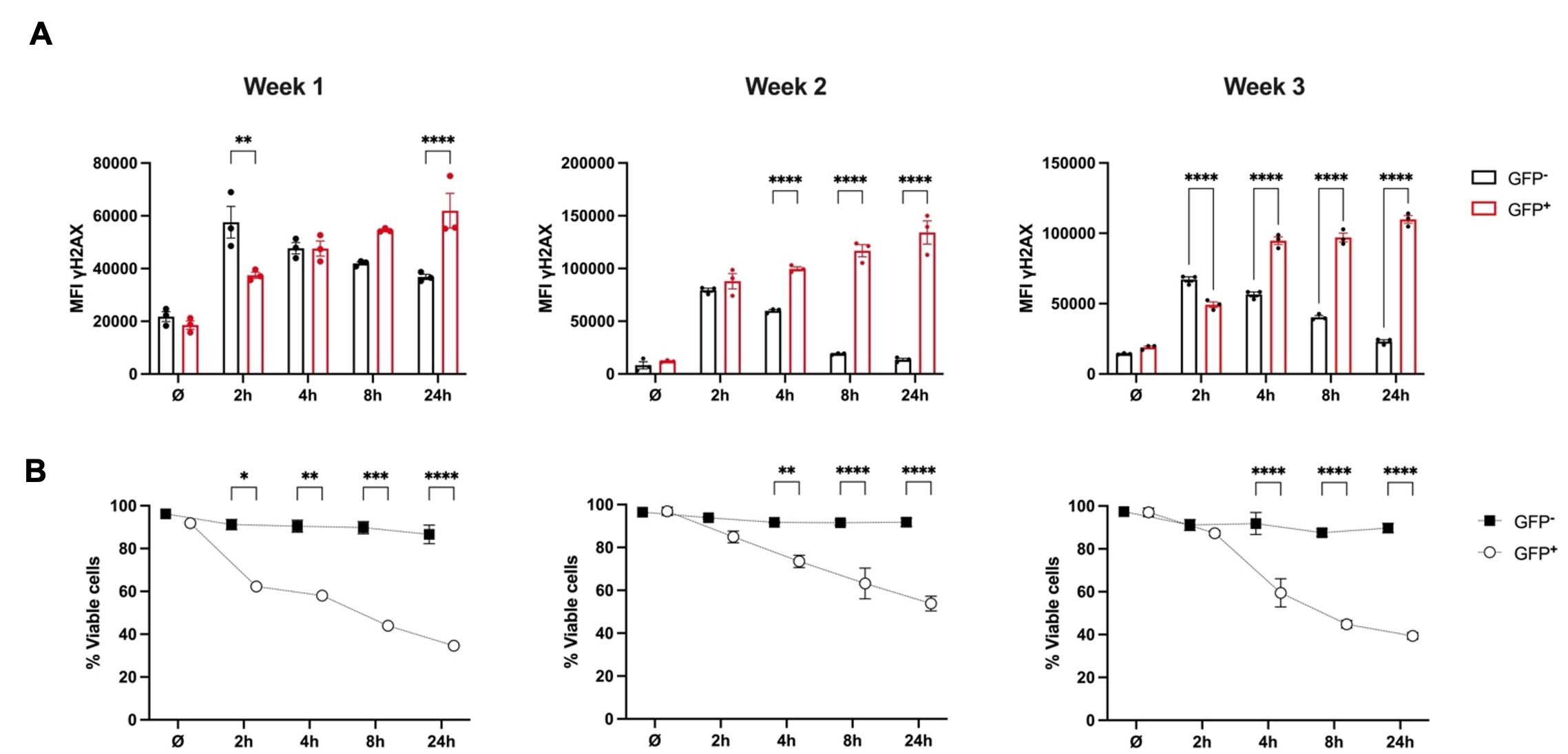

Supplement: Supplementary file 11 [file Image9.jpeg]

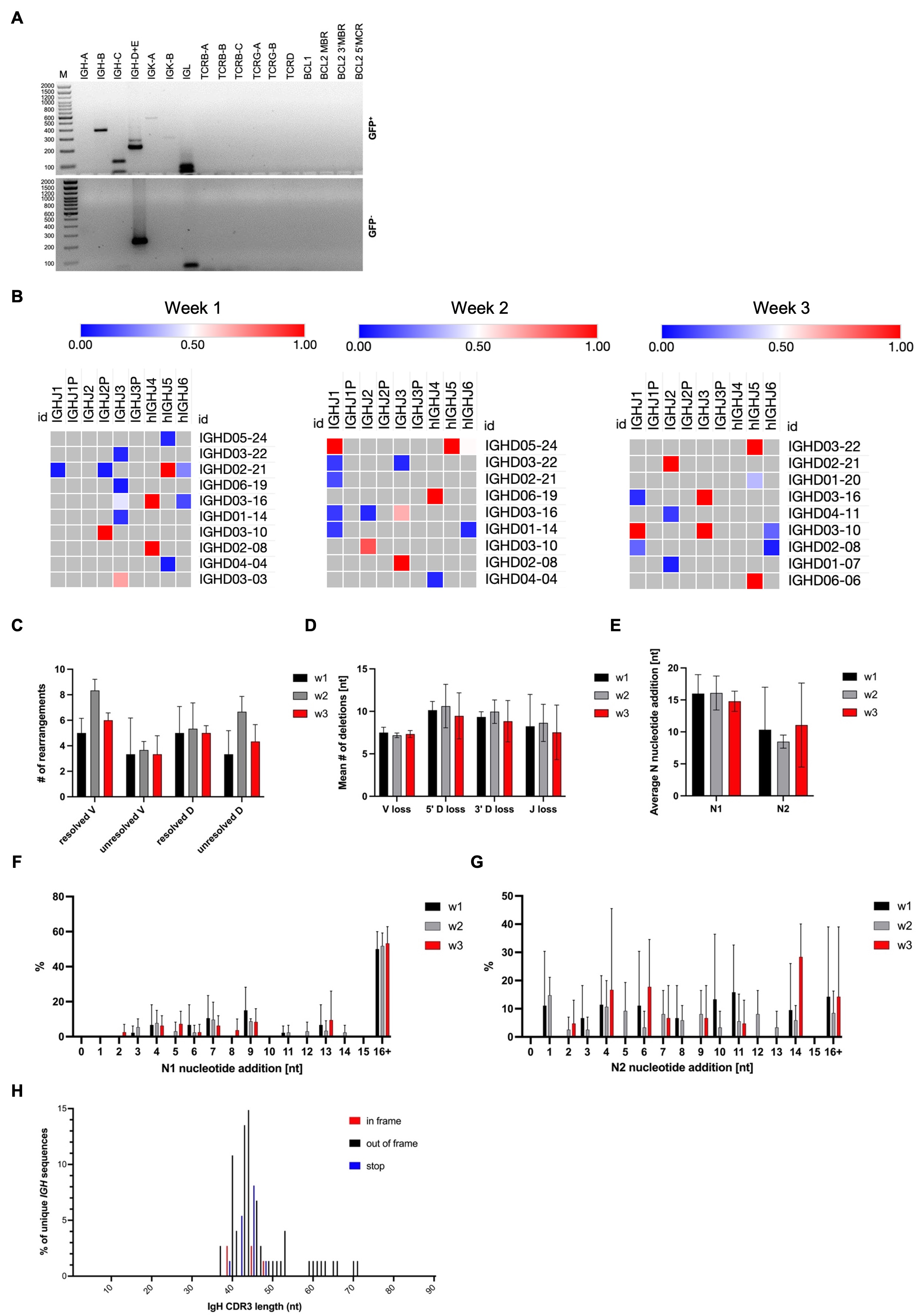

Supplement: Supplementary file 12 [file Image10.jpeg]

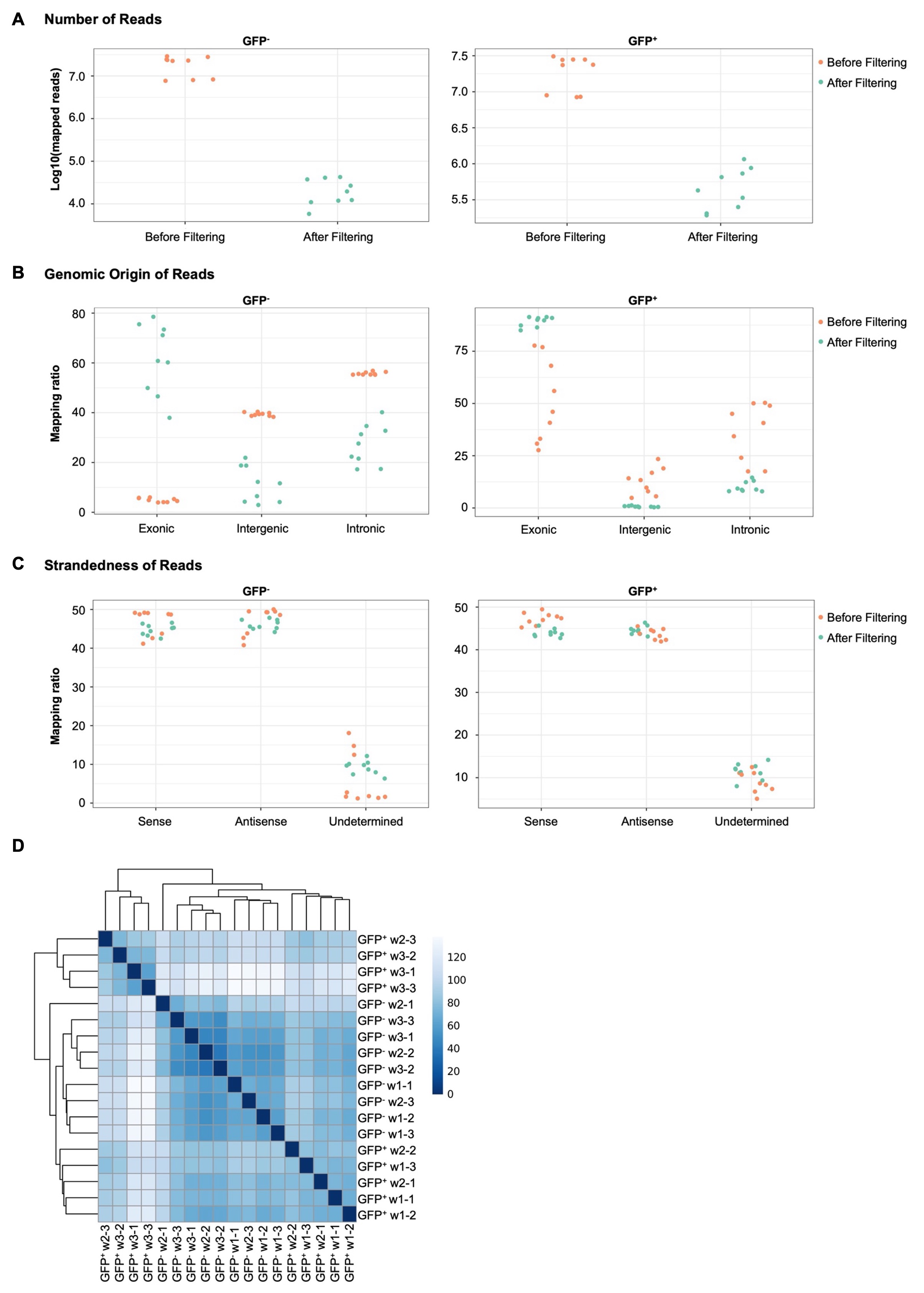

Supplement: Supplementary file 13 [file Image11.jpeg]

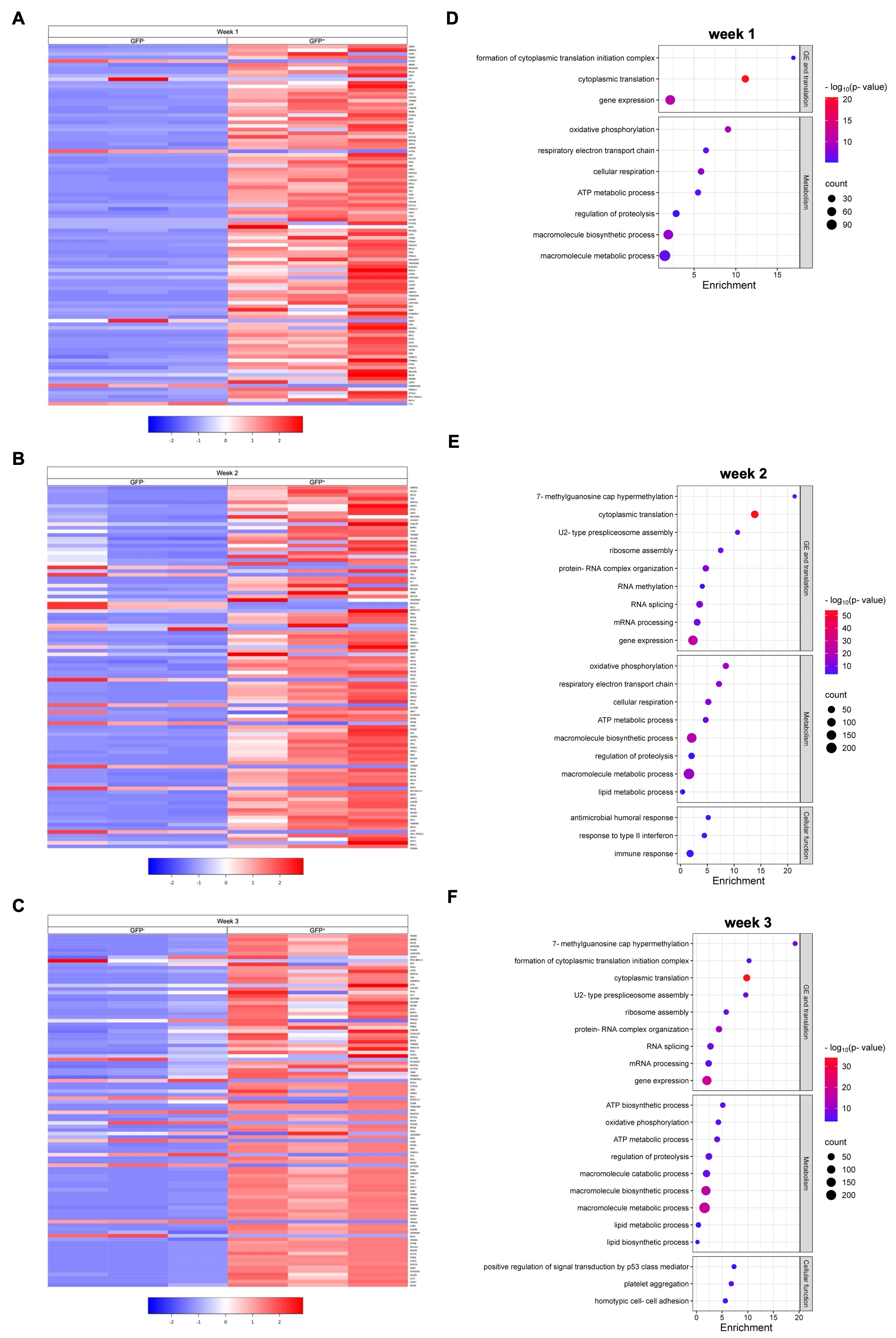

Supplement: Supplementary file 14 [file Image12.jpeg]

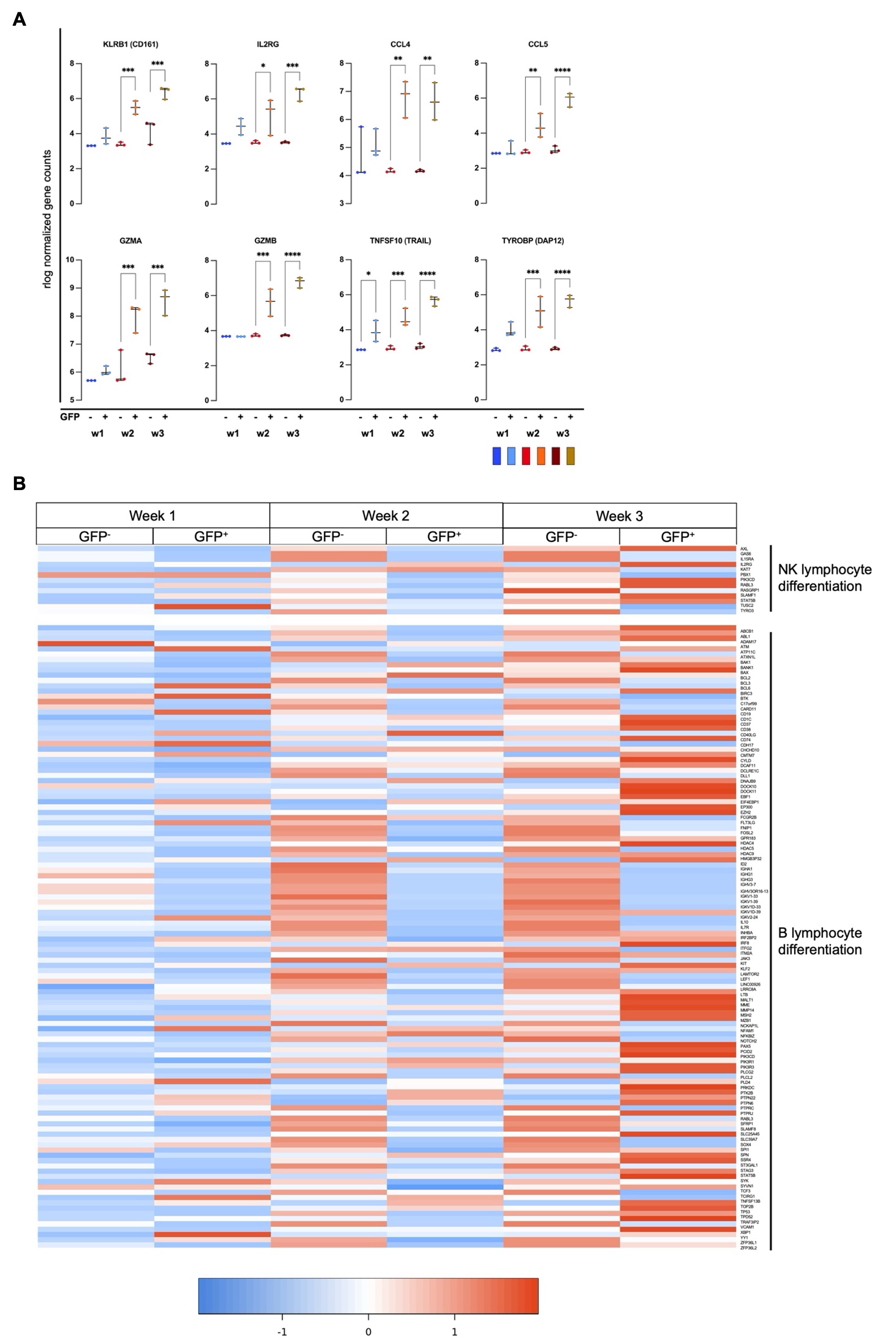

Supplement: Supplementary file 15 [file Image13.jpeg]

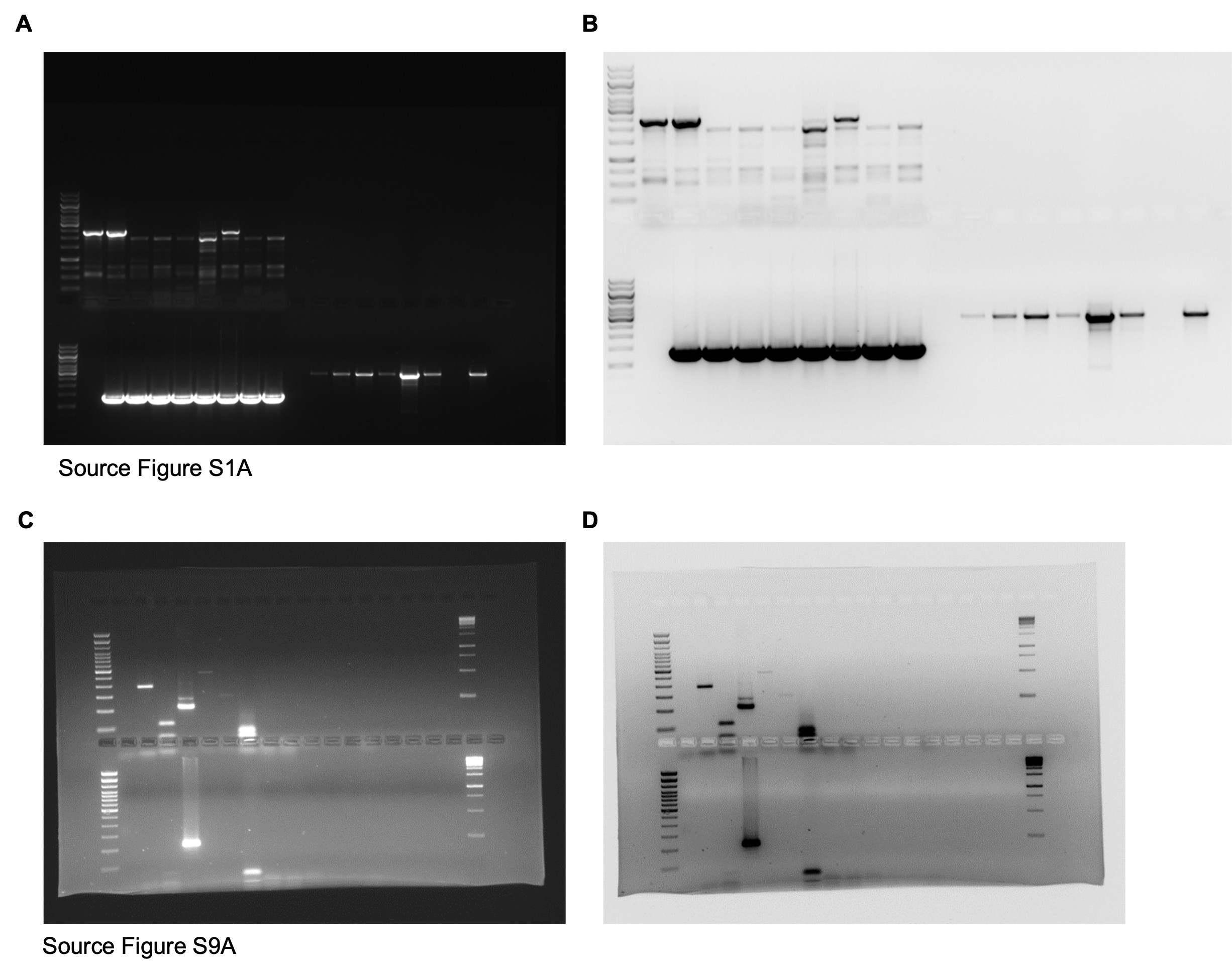

Supplement: Supplementary file 16 [file Image14.jpeg]
